# Single Cell DNA Methylation and 3D Genome Architecture in the Human Brain

**DOI:** 10.1126/science.adf5357

**Published:** 2023-10-13

**Authors:** Wei Tian, Jingtian Zhou, Anna Bartlett, Qiurui Zeng, Hanqing Liu, Rosa G. Castanon, Mia Kenworthy, Jordan Altshul, Cynthia Valadon, Andrew Aldridge, Joseph R. Nery, Huaming Chen, Jiaying Xu, Nicholas D. Johnson, Jacinta Lucero, Julia K. Osteen, Nora Emerson, Jon Rink, Jasper Lee, Yang Li, Kimberly Siletti, Michelle Liem, Naomi Claffey, Caz O’Connor, Anna Marie Yanny, Julie Nyhus, Nick Dee, Tamara Casper, Nadiya Shapovalova, Daniel Hirschstein, Song-Lin Ding, Rebecca Hodge, Boaz P. Levi, C. Dirk Keene, Sten Linnarsson, Ed Lein, Bing Ren, M. Margarita Behrens, Joseph R. Ecker

**Affiliations:** 1 Genomic Analysis Laboratory, The Salk Institute for Biological Studies, La Jolla, CA 92037, USA; 2 Bioinformatics and Systems Biology Program, University of California, San Diego, La Jolla, CA 92037, USA; 3 Division of Biological Sciences, University of California, San Diego, La Jolla, CA 92037, USA; 4 Computational Neurobiology Laboratory, The Salk Institute for Biological Studies, La Jolla, CA 92037, USA; 5 Ludwig Institute for Cancer Research, La Jolla, CA 92037, USA; 6 Department of Medical Biochemistry and Biophysics, Karolinska Institutet; 171 77 Stockholm, Sweden; 7 Flow Cytometry Core Facility, The Salk Institute for Biological Studies, La Jolla, CA 92037, USA; 8 Allen Institute for Brain Science; Seattle, WA 98109, USA; 9 Department of Laboratory Medicine and Pathology, University of Washington, Seattle, WA 98195, USA; 10 Center for Epigenomics, University of California, San Diego School of Medicine, La Jolla, CA 92037, USA; 11 Department of Cellular and Molecular Medicine, University of California, San Diego School of Medicine, La Jolla, CA 92037, USA; 12 Institute of Genomic Medicine, University of California, San Diego School of Medicine, La Jolla, CA 92037, USA; 13 Moores Cancer Center, University of California, San Diego School of Medicine, La Jolla, CA 92037, USA; 14 Howard Hughes Medical Institute, The Salk Institute for Biological Studies, La Jolla, CA 92037, USA

## Abstract

Delineating the gene regulatory programs underlying complex cell types is fundamental for understanding brain functions in health and disease. Here, we comprehensively examine human brain cell epigenomes by probing DNA methylation and chromatin conformation at single-cell resolution in 517k cells (399k neurons and 118k non-neurons) from 46 regions of three adult male brains. We identified 188 cell types and characterized their molecular signatures. Integrative analyses revealed concordant changes in DNA methylation, chromatin accessibility, chromatin organization, and gene expression across cell types, cortical areas, and basal ganglia structures. We further developed scMCodes that reliably predict brain cell types using methylation status of select genomic sites. This multimodal epigenomic brain cell atlas provides new insights into the complexity of cell type-specific gene regulation in adult human brains.

High-throughput epigenomic profiling has been used to elucidate the gene regulatory programs underlying tremendous cellular complexity in brains (1–3). 5’-methylcytosines (5mCs) are the most common modified bases in mammalian genomes. Most 5mCs in vertebrate genomes occur at cytosine-guanine dinucleotides (CpGs). CG differentially methylated regions (DMRs) are often considered indicative of cis-regulatory elements (CREs) (4, 5). In vertebrate neuronal systems, however, 5mCs are also abundantly detected in non-CG (or CH, H=A, C, or T) contexts (6). Both CG- and CH-methylation (mCG and mCH) are highly dynamic during brain development and show cell-type specificity (1, 4, 7). They are also essential for gene regulation and brain functions (8). In addition, gene regulation also requires proper 3D conformation of chromatin folding, which is organized into active (A) or repressive (B) compartments, topologically associating domains (TADs), and chromatin loops (9). These 3D structures facilitate the interaction between gene promoters and their regulatory elements, providing additional but yet critical layers of regulatory mechanisms. DNA methylation and chromatin conformation interplay and coordinate in regulating gene expression and these processes are highly correlated (3). Surveys on these epigenomic features of brain cells can deepen our understanding of gene regulation underlying the complexity of human brains. Here, we comprehensively profiled both DNA methylation and chromatin conformation in adult human brain cells from cortical and subcortical regions using single-nucleus epigenomic sequencing technologies.

## Epigenome-based brain cell type taxonomies

**Figure 1.**
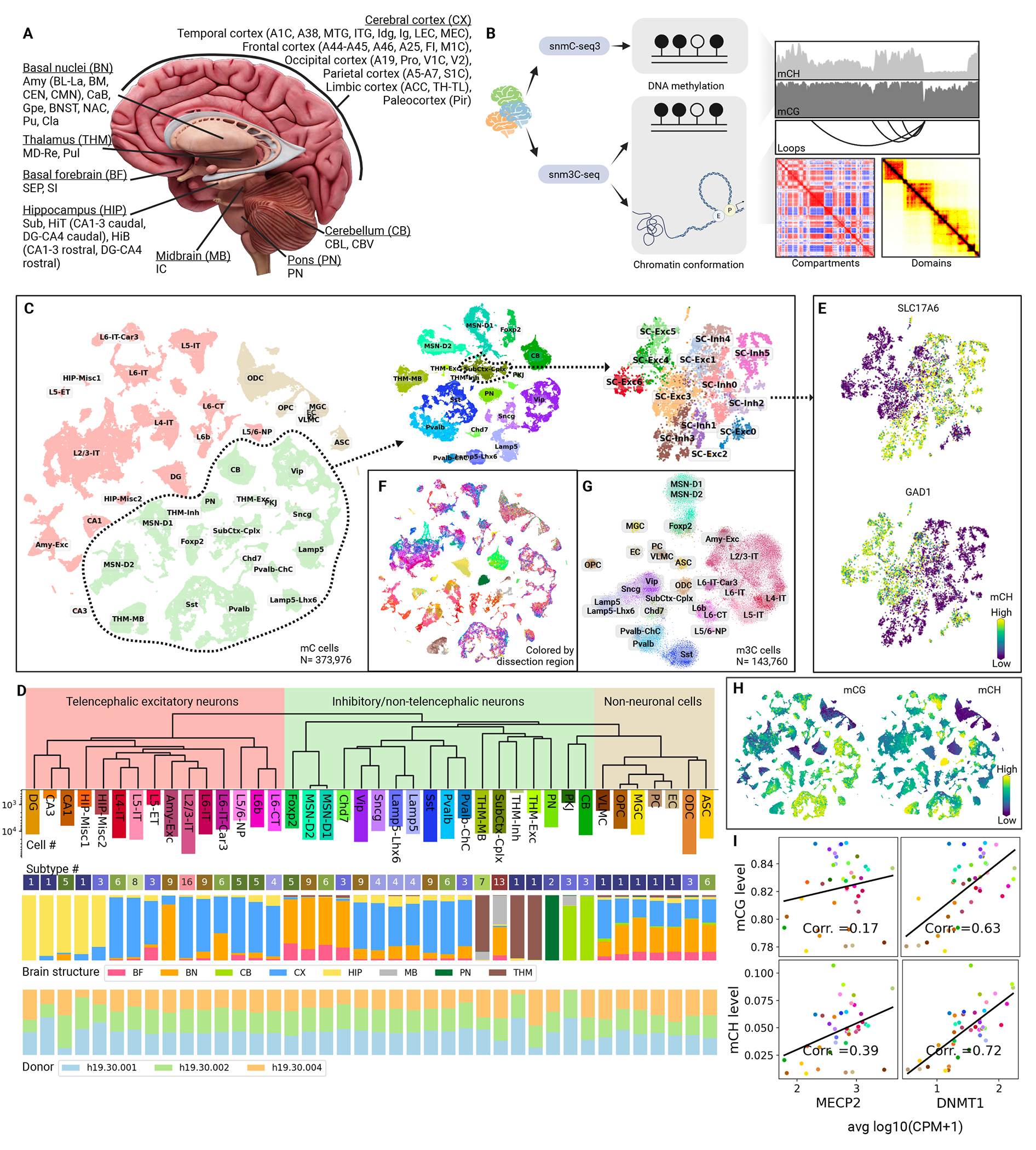
Epigenomic profiling of human brain cells with snmC-seq3 and snm3C-seq. (A) Human brain structures and regions covered. (B) Schematics of profiling modalities of snmC-seq3 and snm3C-seq. (C) Iterative clustering and annotation of human brain nuclei. Cells from the whole mC dataset, from the inhibitory/non-telencephalic neuron cell class, and from the SubCtx-Cplx major type are visualized successively using t-distributed stochastic neighbor embedding (t-SNE), colored by the cell groups annotated in the corresponding iterations. (D) The robust dendrogram of the major types and the meta info of subtype numbers, brain structure, and donor origins. The color palettes are shared across this study. (E) CH-methylation of excitatory and inhibitory markers (SLC17A1 and GAD1) of the major type SubCtx-Cplx. (F) Human brain cells are colored by the dissection regions. (G) 2D visualization of brain nuclei profiled by snm3C-seq. (H) Variation of global CG- and CH-methylation across brain cell types. (I) Correlations between global DNA methylation and gene expressions of MECP2 and DNMT1 across major types.

We dissected 46 brain regions encompassing brain structures of the cerebral cortex (CX, 22 regions), basal forebrain (BF, 2), basal nuclei (BN, 11), hippocampus (HIP, 5), thalamus (THM, 2), midbrain (MB, 1), pons (PN, 1) and cerebellum (CB, 2) (Fig. 1A, fig. S1A, and tables S1 and S2). Most regions had three biological replicates from the three adult male donors (table S3) except two amygdala regions (BM and CEN; two replicates each) (fig. S1A and table S1). Fluorescence-activated nuclei sorting (FANS) was used to isolate 90% NeuN-positive and 10% NeuN-negative cells in each sample (fig. S1A). We then employed snmC-seq3 (“mC”)(10) to profile DNA methylation (DNAm) across all 46 brain regions at the single-cell level. Additionally, we utilized snm3C-seq(“m3C”)(3) to simultaneously examine single-cell DNA methylation and chromatin conformation from 17 brain regions spanning CX, BF, and BN (See Fig 1B, and fig. S1A). Following rigorous quality control, 378,940 mC and 145,070 m3C nuclei were confirmed suitable for further analysis (fig. S1B). Each mC cell produced an average of 0.94 million filtered reads, and each m3C cell produced around 2.20 million reads with 406k chromatin contacts. This data quality allowed us to reliably measure DNAm across genomic features (fig. S1C), identify variable methylation regions, and pinpoint TADs and chromatin loops across different brain cell types.

Through iterative clustering of the mC dataset (Methods), nuclei were first divided into three classes: telencephalic excitatory neurons, inhibitory/non-telencephalic neurons, and non-neuronal cells (Fig. 1, C and D). These were further divided into 40 major types and 188 subtypes (Fig. 1D, fig. S2, A to C, and tables S4 and S5). The cell types were annotated based on CH-hypomethylated gene markers for neuronal cells and CG-hypomethylated markers for non-neuronal cells (Methods). All major types and subtypes were conserved across donors, though there were minor variations in the proportion of certain cell types (Fig. 1D and fig. S2C). The robust dendrograms demonstrated similarities between major types and subtypes (Fig. 1D and fig. S2C; Methods). Telencephalic excitatory and inhibitory/non-telencephalic neurons are well-separated from non-neuronal cells, each type forming a specific clade except CB and PKJ, which were grouped with the non-neuronal cell types, likely owing to their similar global CG- and CH-methylation fractions (Fig. 1H and fig. S4A).

Non-neuronal major types distribute evenly across brain structures, whereas neuronal ones exhibit considerable spatial specificity (Fig. 1, D and F). Most telencephalic excitatory neurons were grouped by location (Fig. 1D). Hippocampal excitatory neurons were grouped based on their sub-structures (CA1, CA3, & DG). Cortical excitatory neurons were clustered by their cortical layers (like L2/3; L=layer) and projection types (like IT) (table S4). Basal nuclei excitatory neurons, predominantly from the amygdala, form the Amy-Exc group. Telencephalic inhibitory neurons manifest as eleven major types, primarily from cortical areas (Pvalb, Pvalb-ChC, Sst, Lamp5, Lamp5-Lhx6, Sncg, and Vip) and basal nuclei or basal forebrain (MSN-D1, D2, Foxp2, and Chd7). In the thalamus, one excitatory and two inhibitory major types were identified. One inhibitory major type, THM-MB, shares similar DNA methylation profiles with a small population of midbrain cells. The other inhibitory major type, THM-Inh, is very rare (361 cells or 0.07% of the entire dataset), possibly originating from the habenular nuclei of the thalamus due to dissection contamination (fig. S2D). Pontine nucleus neurons constitute a unique major type (PN). The cerebellum contained two distinct major types: the rare cell type Purkinje cells (PKJ, 867 cells or 0.17%), and cerebellar granule cells (CB). Lastly, the SubCtx-Cplx major type, found in the basal nuclei and midbrain, was notable for its heterogeneity: its subtypes consisted of both excitatory and inhibitory cells (Fig. 1E) and featured highly variable DNAm of the genes of neurotransmitter receptors, transporters, and neuropeptides (fig. S2E).

The cell types determined from single-nucleus DNAm profiles were corroborated with single-nucleus transcriptome (snRNA-seq) and single-nucleus chromatin accessibility (snATAC-seq) data from the same human brains (Methods; companion manuscripts Siletti et al. (11) and Li et al. (12)). Integrative analysis revealed the strong correspondence between cell types determined using different molecular modalities (fig. S3A). All epigenome-based cell subtypes correspond well with transcriptome-based clusters (fig. S3B), though the transcriptome-based clusters were derived from ~10 times more cells and from ~2 times more brain regions.

Global methylation varied among major types: 77.7%-85.5% for mCG and 0.8%-10.7% for mCH. Non-neuronal and granule cell (DG and CB) major types had the lowest global fractions in both mCG and mCH (Fig. 1H and fig. S4A), consistent with the previous study in mice (1). Cortical inhibitory neurons have the highest mCG, whereas certain non-telencephalic neurons from the thalamus, midbrain, and pons exhibited the highest mCH (Fig. 1H and fig. S4A). Cell-type global methylation corresponded with the gene expression of DNAm readers and modifiers (Fig. 1I and fig. S4B). The expression of MECP2 and DNMT3A, the major mCH reader and writer, were positively correlated with global mCH (Pearson Correlation Coefficient, PCC=0.39 and 0.35) and weakly with mCG (PCC=0.17 and 0.08; Fig. 1I and fig. S4B). The DNA methyltransferase DNMT1 had a high positive correlation (PCC=0.63) between its expression and mCG across cell types (Fig. 1I), matching its role as the major mCG maintainer in mature neurons (13). Intriguingly, we observed an even higher correlation between DNMT1 expression and mCH (PCC=0.72, Fig. 1I), though it is thought to have little effect on mCH (14). This implied an unknown relationship between DNMT1 and mCH or some yet-to-be-discovered factor influencing both DNMT1 expression and mCH.

Using the improved scHiCluster(15) for m3C cells, we were able to separate all major types except MSN-D1 and D2 solely through chromatin contacts (Fig. 1G). This also highlighted the diversity of chromatin conformation across brain regions (fig. S2F). To ensure the consistency of annotations between the two datasets, we co-clustered mC and m3C cells iteratively and then transferred cell type annotations from mC to m3C cells (table S5; Methods).

## Differences in contact distance between neurons and non-neurons

**Figure 2.**
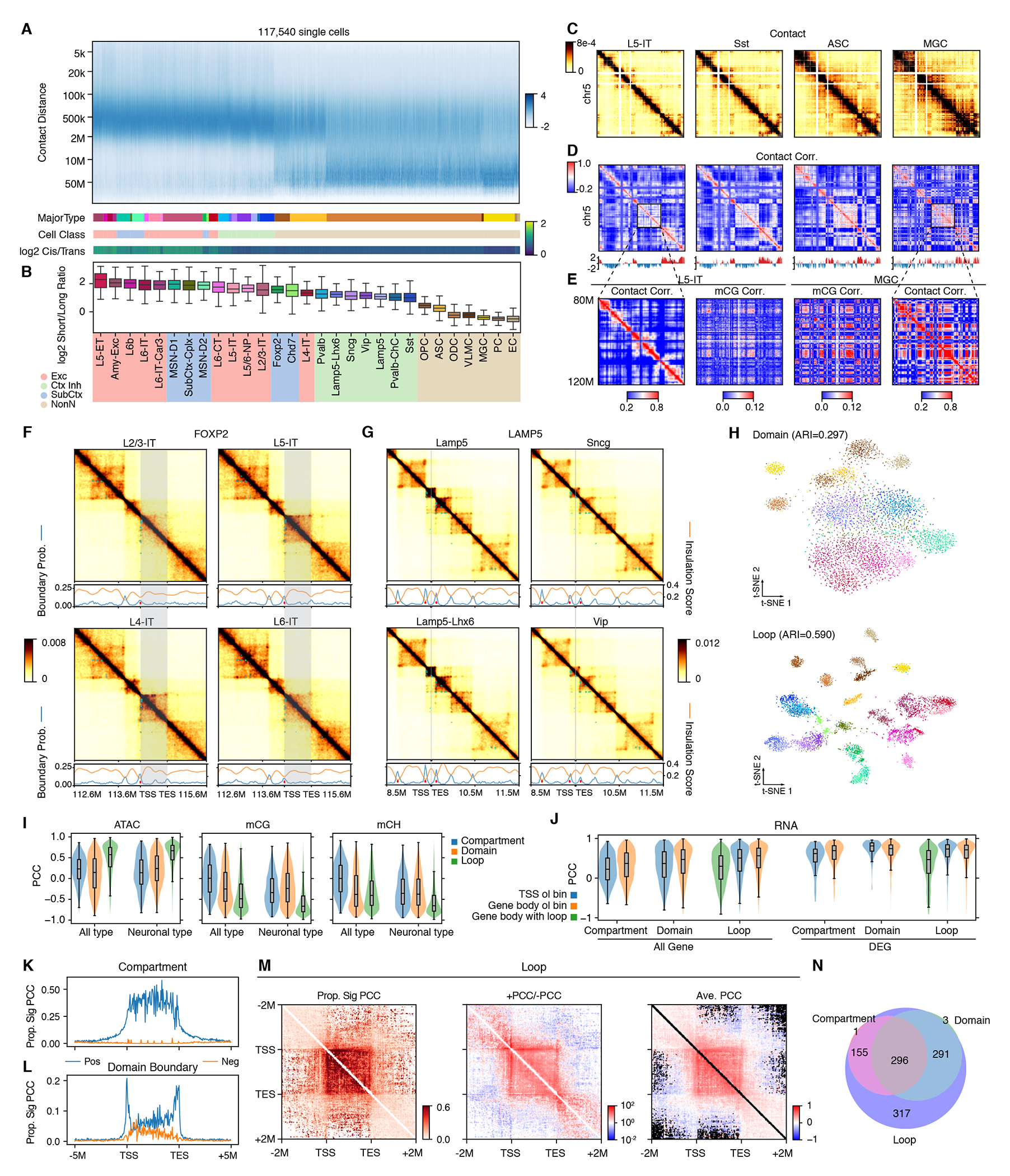
Diversity of 3D genome structures across major types. (A) Frequency of contacts against genomic distance in each single cell, Z-score normalized within each cell (column). The cells are grouped by major type and then ordered by the median log2 short/long ratio over cells. The y-axis is binned at log2 scale. (B) log2 short/long ratio of major types, ordered the same as in (A).(C) Imputed contact maps of four major types. (D) Heatmaps show the correlation matrices of distance normalized contact maps in (C), and line plots show the first principal component of the correlation matrices. (E) Zoom in view of two matrices in (D) and the corresponding correlation matrices of mCG across cells. (F and G) Imputed contact matrices (heatmap), boundary probabilities (blue lines), insulation scores (orange lines), differential boundaries (red dots in line plots), and differential loops (cyan dots in heatmaps) of excitatory IT neurons at FOXP2 locus (a marker of cell type L4-IT; F) or CGE-derived inhibitory neurons at LAMP5 locus (a marker of Lamp5 and Lamp5-Lhx6; G). Grey shade represents the gene body (TSS to TES). (H) t-SNE plot of cells (n=5,707) using domains (top) or loops (bottom) as features, colored by major types. (I) PCC between compartment score, boundary probability, or loop interaction strength and ATAC signals, mCG and mCH fractions of the bin(s) across all major types for all genes (left) or top DEGs only (right). (J) PCC between compartment scores, boundary probabilities, or loop interaction strength and gene expression across all major types for different categories of overlap (x-axis) using all genes (left) or top DEGs (right). (K and L) Proportion of significantly positively or negatively correlated compartment (K) or domain boundary (L) out of all the bins located at different positions relative to a gene, average across the top neuronal DEGs. (M) Proportion of significantly correlated loop pixels out of all the loop pixels (left), ratio between positively and negatively correlated loop pixels (middle), or average PCC of significantly correlated loop pixels (right) located at different positions relative to a gene, average across the top neuronal DEGs. (N) The number of genes, out of the top neuronal DEGs, having significantly positively correlated compartments, domain boundaries overlap the gene body, or loop pixels within the gene body or with at least one anchor overlaps the TSS or TES of the gene. 35 genes were not included in any of the three circles.

To investigate cell-type-specific genome folding at different scales, we first examined the proportion of contacts per cell at genome distances. Neurons displayed enrichment of interactions at a shorter distance (200kb-2Mb), whereas mature oligodendrocytes and non-neural cells were enriched for longer-range contacts (20Mb-50Mb). Astrocyte and oligodendrocyte progenitor cells exhibited enrichment in both ranges (Fig. 2, A to C, and fig. S5, A and B). Within neuronal cells, cortical excitatory and subcortical neurons had more shorter-range interactions than cortical inhibitory cells (p-value<1e-300, Wilcoxon rank-sum test; Fig. 2, A and B). We observed similar patterns in previous datasets from the mouse (1) and from a different technique (Dip-C (16); fig. S5C), signifying the conservation of these patterns. The enrichment of shorter-range contacts in neurons was observed across the whole genome, including both neuronal and non-neuronal gene loci (fig. S5D). The ratio between shorter and longer interactions highly correlated with global gene expression activity of cells (PCC=0.87, fig. S5E), and aligned with the sizes of nuclei (L5-ET > other cortical excitatory neurons > cortical inhibitory neurons > non-neurons (17)). These results demonstrated that the contact distance spectrum, traditionally associated with cell-cycle phases (18), can also vary based on cell type in non-dividing cells.

We next investigated the relationship between enriched longer-range or shorter-range chromatin interactions and chromatin compartments or domains. We identified chromosome compartments within each major type at 100 kb resolution (Fig. 2D) and domains at 25 kb resolution. Enriched longer-range interactions in non-neurons were predominantly intra-compartment, especially between B compartment regions. Shorter-range interactions in neurons were also enriched within the same compartments (fig. S5, F and G). In total, we observed an enrichment of intra-compartment interactions and a depletion of inter-compartment interactions in non-neurons (fig. S5, H to K; Methods), indicating a stronger compartment strength. In contrast, the enrichment of short-range interactions in neurons was found to be both intra- and inter-domain (fig. S5, L and M).

## Compartments, domains, and loops in brain cell types

We postulated that the methylation status of two genome loci would co-vary if they were physically proximate. The co-methylation coefficient matrices, depicting the correlation of methylation between genomic bins across single cells, displayed plaid patterns echoing the compartment structures of chromatin contacts (Fig. 2E and fig. S6A). This suggested the genome was segregated into local co-methylation domains, which constituted two sets with opposite methylation diversities. A similar coregulation structure was also observed for chromatin accessibility in single-cell ATAC-seq data (19), reinforcing evidence for genome compartmentalization. Exploring the linking between DNA methylation and 3D genome architecture, we observed that correlations between the strengths of chromatin interactions and the average methylation fractions of their anchors were also associated with chromosome compartments (fig. S6B), where negative correlations occurred more frequently in the active compartment (p-value<1e-300; fig. S6C).

We then determined domains at 25 kb resolution in single cells and found that neurons had more domains (median 4,813) than non-neurons (median 4,308, p-value<1e-300) but with smaller average size, resulting in a similar domain-covered genome proportion (fig. S6, D and E). The number and size of domains were highly correlated with global gene expression activity (fig. S6F). The boundary probability of a genomic bin was defined as the frequency it was identified as a domain boundary across cells, which mirrored the insulation scores from the cell-type pseudo-bulk contact maps (Fig. 2, F and G).

Chromatin loops were delineated at 10 kb resolution in each of the 29 major types (and 119 cell subtypes) with >=100 m3C cells. We detected a median of 524,935 (541,551) loop pixels with 45,140 (59,905) loop summits among major types (subtypes) (fig. S6G). Of these, 24.3% were interactions between distal DMRs (see later section for systematic description of DMRs) and gene promoters (TSS±2kbp), 38.1% between distal DMRs, and 5.8% between promoters (fig. S6H).

## Cell type specificity of 3D genome features

Using either compartment scores, domain boundary probabilities, or loop strengths, we were able to distinguish between cell types and determine the hierarchy of their similarities (Methods; Fig. 2H and fig. S7, A to C), indicating cell type specificities of these 3D structures. Particularly, chromosome compartments could distinguish non-neurons, excitatory, inhibitory, and MSN neurons, but had difficulty for finer major types within the excitatory or inhibitory cell classes (fig. S7, B and C). In contrast, both chromatin domains and loops distinguished better for finer excitatory and inhibitory major types, and loops performed the best (Fig. 2H and fig. S7, B and C). This underscores the varying roles of different scales of 3D features in gene regulation across cell type granularities, highlighting that loops could be more specific than domains. Note that the primary goal of these analyses was to contrast compartments, domains, and loops in cell type specificity, but not for cell type clustering. The state-of-the-art of cell clustering on chromatin contacts is still based on the genomic bin-pairs, as adopted by us (15) or other groups (20, 21) (fig. S7, B and C; more discussion in Methods).

Systematic examination on specific 3D structures across all (or neuronal) major types determined 1,188 (1,024) differential compartments (DCs), 2,050 (1,720) differential domain boundaries (DBs), and 173,806 (148,395) differential loops (DLs) (fig. S8A and table S6). Chromatin domains were considered conserved across cell types in general (22–25), whereas they could display certain dynamics across cell types and development (3, 26–28). Our data further showed that chromatin domains could vary even between closely-related cell types (Fig. 2, F and G). DMR-DMR loops showed higher cell-type specificity than promoter-DMR or promoter-promoter loops (fig. S8B). Evaluating transcription factors (TFs) in differential chromatin looping, we found the motifs of cell type-specific TFs (like NFIX and NHLH1) were more enriched at anchors of DLs, whereas CTCF, a TF pivotal for chromosome structure, was highly enriched at housekeeping loops (fig. S8C). This implied CTCF’s role is more in structural loops than in cell type-specific promoter-enhancer interactions. Many neuronal TFs (like NEUROG1 and NEUROG2) were enriched at the pan-neuronal loops but not pan-brain-cell loops (fig. S8C), concordant with their neuron-specific roles.

## Relationship between genome organization and other molecular modalities

We examined the link between different 3D structural features and other epigenomic modalities (mCG, mCH, and open chromatin). Across neuronal cell types, both mCG and mCH were anti-correlated with compartment scores, domain boundary probabilities, and loop strength (Fig. 2I, figs. S9, B and D, and S10, C and D). In contrast, open chromatin signals exhibited positive correlations with these structural features with similar or slightly weaker (absolute) correlations (Fig. 2I, figs. S9, B and D, and S10, C and D). These (anti-)correlations suggest orchestration among active compartments, strong domains and loop interactions, as well as open chromatin and methylation depletion corresponding to active chromatin states. Between the differential structural features (DCs, DBs, and DLs) across cell types, DLs had stronger (anti-)correlations with mCG, mCH, and open chromatin compared to DCs and DBs (fig. S10C), particularly at the loops with high variability across cell types (fig. S10D). Correlations across all cell types were generally weaker than in neurons alone (Fig. 2I, and figs. S9 and 10). The anticorrelation observed between DNAm and 3D genome structures could have resulted from the effect of DNAm on the binding of factors driving genome folding (like CTCF)(29), the recruitment or exclusion of methylation writers or erasers (such as DNMTs and TETs) through high-order structural formation, or shared regulators of both methylation and genome organization (for example, Neurog2 in mouse cortex (30)). Further developmental or mechanistic studies are needed to resolve the causality relationship (29, 31).

Gene expression was correlated with the 3D genome structures as well, particularly for the cell-type-specific genes (Fig. 2J). We identified 1,099 (1,358) top differentially expressed genes (DEGs) pairwisely across neuronal (all) major types. They exhibited strong positive correlations with all three structural features that overlapped with their gene bodies or promoters (Fig. 2J and figs. S11 to 13). For loops, the interaction strengths were more correlated with anchor-overlapped DEGs (on gene bodies or promoters) compared to the anchor-encompassed DEGs (p-value<1e-300; Fig. 2J). We also noticed that increasing variability of gene expression and/or structural signals of bins was linked to higher positive correlations between them, which corroborates the overlap between differential structural signatures and differential gene expression (figs. S11, E and F, and S12, E and F).

We further examined the relative location between 1,099 neuronal DEGs and their correlated chromatin structures (FDR<0.01, Methods) at surrounding regions (TSS-5Mb to TES+5Mb). The correlated compartments were mostly within the gene body (Fig. 2K), and the correlated domain boundaries were highly enriched at TSS and TES (Fig. 2L), suggesting the dynamics of gene body compartments and domains associated with gene expression diversity. The loops with positive correlations were enriched within gene bodies, as well as between the TSS/TES and the gene body ± 1 Mb regions (Fig. 2M). Specifically, 48% of the loops within the gene body were correlated with gene expression, among which 98% are positively correlated. In comparison, a much smaller proportion of loops outside the gene body were correlated with expression. A higher proportion of positively correlated loops were observed within the upstream and downstream regions of the DEGs, and between the upstream or downstream and the gene body regions, indicating the regulatory domain of a gene structure.

Among the 1,099 DEGs, 453 (41.2%) had gene bodies overlapped by one or more genomic bins with positively correlated compartment scores, and 591 (53.8%) overlapped by one or more correlated domain boundaries. 1,037 (94.4%) DEGs had TSS- or TES-anchored correlated loops, and 898 (81.8%) had correlated loops within gene bodies. These dynamics of chromatin architecture at different scales in total covered 96.8% of the DEGs (Fig. 2N), again suggesting a strong association between genome structures and gene expression diversity. Collectively, these analyses revealed the cell-type specificity of chromatin architecture and its relationship with other epigenomic and transcriptomic signatures at an unprecedented cell-type resolution in the human brain.

## Cell-type specific DNA methylation patterns and associated gene regulatory landscapes

**Figure 3.**
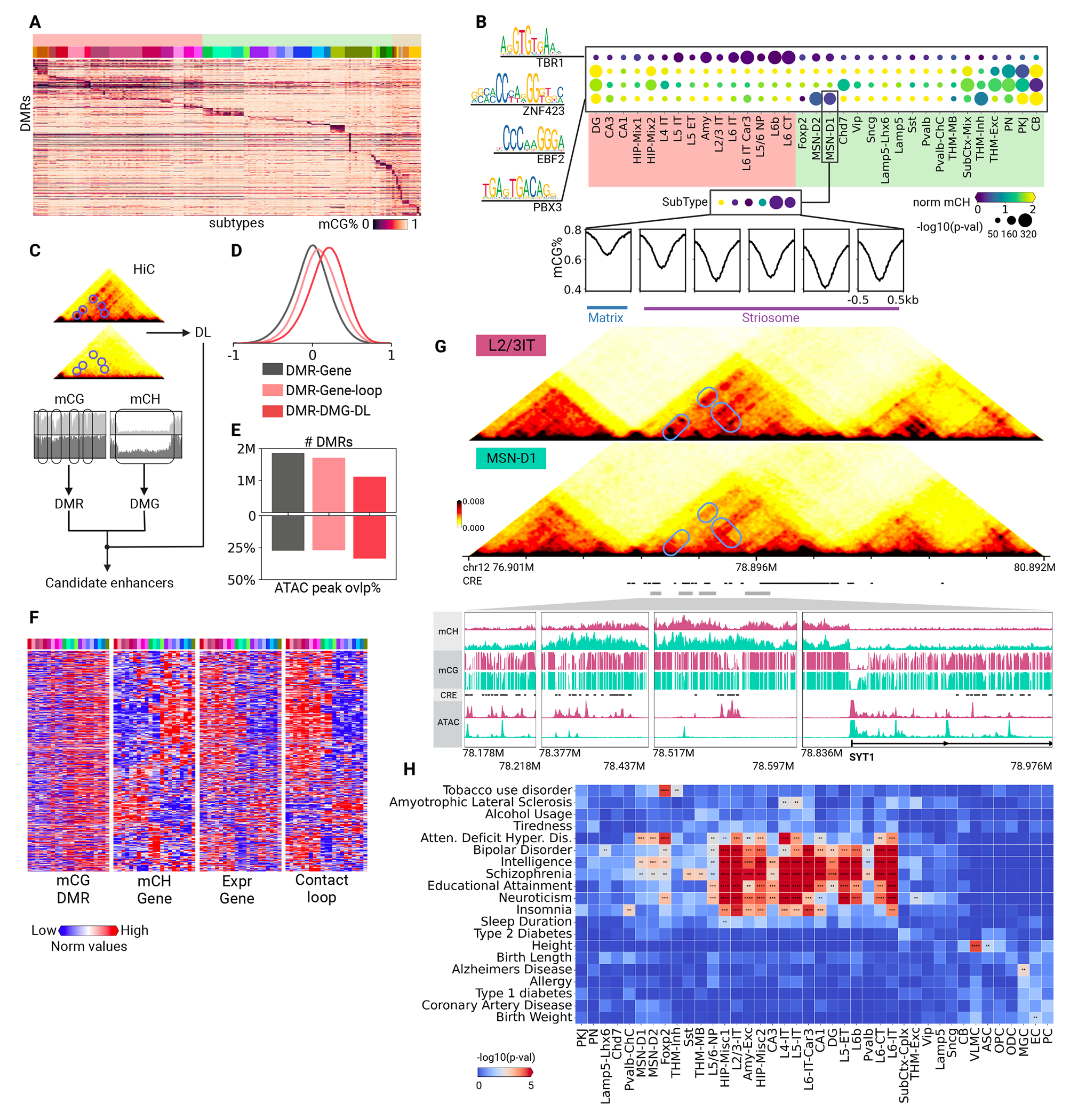
Gene regulation in brain cells. (A) mCG of cell type-specific DMRs across 188 cell subtypes. (B) CH-hypomethylated transcription factors and the enrichment of their motifs in CG hypo-DMRs. The lower panel showed average methylation fractions of transcription factor PBX3 in its potential binding sites across the whole genome. (C) Workflow of determining putative CREs. (D) Distribution of correlation between methylation of putative CREs and the corresponding genes from different filtering. (E) Numbers of putative CREs and overlapping proportions with open chromatin regions for different filtering. (F) Heatmaps showing mCG of putative CREs, mCH and expression of the target genes, and contact strength of the corresponding loops. (G) The gene body mCH, DMR mCG, and 3D chromatin organization around the gene SYT1 in the major types L2/3-IT and MSN-D1. (H) Heatmap showing the results of LDSC analysis of the variants associated with the indicated traits or diseases in DMRs identified from major human cell types. The asterisks indicate the magnitude of p-values (*=−1, **=−2, ***=−3, and ****=−4).

To delineate the cell type-specific methylation profiles, we identified 24,455 CH- and 13,096 CG-differentially methylated genes (DMGs; fig. S14A; Methods) and 2,059,466 CG-DMRs (Fig. 3A; Methods) across 188 brain cell subtypes. In addition to depicting distinct epigenetic signatures for brain cell identities, these methylation patterns provide critical insight into understanding gene regulatory programs in brain cells, with gene body methylation negatively correlating with gene expression (5, 7, 32), DMRs marking putative cis-regulatory elements (CREs) (4, 5)), and transcription factor (TF) motifs implicating candidate cell-type-specific regulators (32).

We assigned TFs to specific cell types if they were hypomethylated DMGs (fig. S14B; Methods) and their motifs were enriched at the hypomethylated DMRs (hypo-DMRs) in the same cell types (Methods). In total, 612 TFs were assigned to major neuronal types and subtypes, where they potentially play important roles in shaping and maintaining cell identities. For example, TBR1 was assigned to deep-layer excitatory neurons, particularly L6-CT and L6b (Fig. 3B), and it was noted to play a fate-determining role in the development of corticofugal projection neurons (33). ZNF423 and EBF2 were both assigned to the cerebellar cell types (Fig. 3B). Both of them are crucial for cerebellum development, whereas EBF2 particularly directs the migration of Purkinje cells (34–36).

Analyzing subtypes further highlighted variations in TF utilization. For instance, the TF PBX3, assigned to the MSN-D1 major type prevalent in the striatum, was only hypomethylated in the subtypes from the striosome compartment but not the matrix compartment of the striatum (Fig. 3B and fig. S14C). This indicates a preference for PBX3 expression in the striosome, corroborating previous observations (37, 38). Further examination of potential binding sites of PBX3 (hypo-DMRs with PBX3 motifs) showed lower average methylation fractions in striosome subtypes (Fig. 3B), suggesting a compartment-specific regulatory role of this TF in the striatum.

We integrated DMGs, DMRs, and differential loops to pinpoint putative CREs for each cell type (Fig. 3C). A gene was associated with a DMR if its TSS was within 5 Mb of the DMR. Further refinement retains only DMR-DMG pairs overlapping with both anchors of a loop or DL. Pearson correlations between mCG fractions of DMRs and mCH fractions of gene bodies across cell subtypes were calculated to assess the association (fig. S14D). Enhanced associations were observed particularly for DL-filtered DMRs (Fig. 3D and fig. S14D), which showed an increased overlap with open chromatin regions as well (Fig. 3E). We identified 3.2M potential regulatory DMR/gene pairs between 1,122,919 DMRs and 12,327 genes (table S7). The methylation fractions of these DMRs, DMGs, and the strengths of their interactions (loops) were (anti-)correlated (Fig. 3F), which could collectively orchestrate specific gene regulatory programs. For instance, the gene SYT1, encoding Synaptotagmin-1—a critical synaptic vesicle protein—exhibited lower methylation fractions of both the distal DMRs and the SYT1 gene body in L2/3-IT neurons and stronger interactions between the DMRs and the promoter compared to MSN-D1 neurons (Fig. 3G), leading to a higher expression of SYT1 in L2/3-IT than MSN-D1 (Fig. 3G and fig. S14E). Overall, the integration of CG- and CH-methylation with chromatin conformation reveals distinct cell-type regulatory dynamics.

Numerous non-coding loci linked to brain diseases have been pinpointed by GWAS, with many in enhancer regions (39). DMRs and loops help localize these genetic variants to specific cell-type regulatory elements. Using linkage disequilibrium score regression (LDSC) (40), we detected associations between 20 brain diseases or traits and DMRs or loop-overlapping DMRs in human brain cells (Fig. 3H and fig. S15A; Methods). Schizophrenia, bipolar disorder, and neuroticism risk variants were prominently enriched in hypo-DMRs of excitatory neurons in the cortices and hippocampus, whereas Alzheimer’s disease (AD) aligned with microglia (MGC; Fig. 3H; (41)). Tobacco usage disorder variants associated with the Foxp2 cell type from the basal ganglia (Fig. 3H), an area linked to tobacco addiction (42). Further exploration into disease risk variants revealed diverse impacts on gene regulations. Although many cell types are related to the same diseases, the risk variants to which they are implicated could be diverse. For example, the schizophrenia risk variants rs2789588 was implicated in both L2/3-IT and L6-CT neurons with similar epigenetic features, whereas rs17194490 was only implicated in L2/3-IT with specific DNA hypomethylation, stronger long-range interaction with the corresponding gene, and higher gene expression compared to L6-CT (fig S15B).

## Regional heterogeneity in cortices and basal ganglia

**Figure 4.**
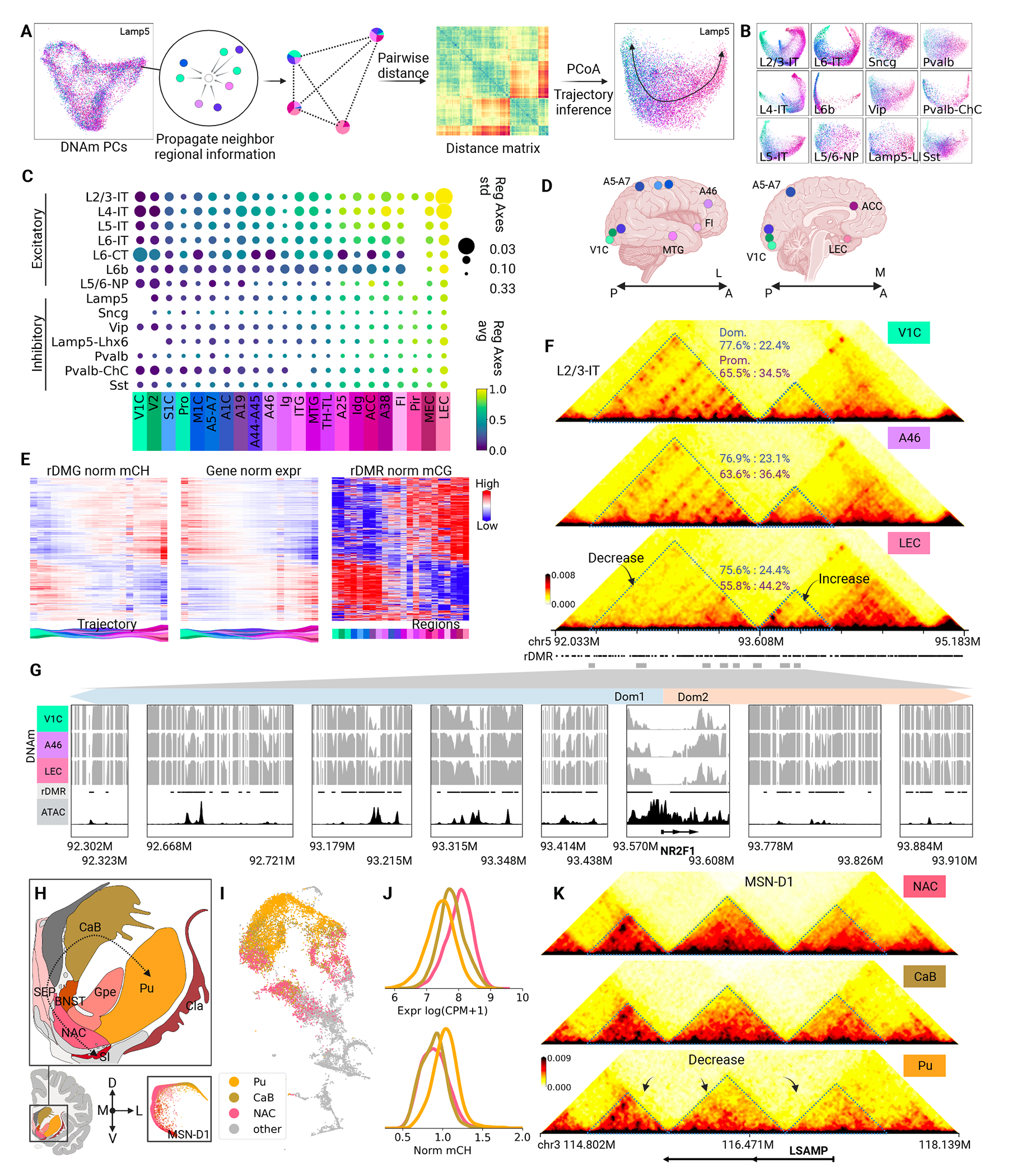
Regional axes of cortical and subcortical cells. (A) Workflow of determining regional axis from single-nucleus DNAm. (B) 2D visualization of cortical neurons in regional spaces, colored by dissection locations. (C) The common regional axis among cortical neurons. The scatter plot showed how regional indices vary in each cortical region. (D) Schematic of example cortical dissection locations. (E) Regional gradients in mCG of rDMRs, and mCH and expression of rDMGs in L2/3-IT cells. (F) Regional difference in chromatin conformation around NR2F1. The blue and purple numbers showed respectively the relative domain strength and promoter strength of each domain. (G) Zoom-in view of example differential-loop-overlapping rDMRs marked in F. In the decreasing domain (left), the methylation fractions increase from V1C to A46 to LEC, while the methylation fractions decrease in the increasing domain (right). (H) Inhibitory neurons in basal ganglia showed an L–D–V axis in DNA methylation (I) 2D t-SNE visualization of MSN-D1. Cells from NAC, CaB and Pu were highlighted. (J) Regional differences of gene body mCH-methylation and expression of LSAMP in MSN-D1. (K) Regional difference in chromatin conformation around LSAMP in MSN-D1.

Beyond cell type diversity, heterogeneity within shared cell types across regions has been noted in the neocortex in both gene expression (43–45) and DNA methylation (1). Our extensive epigenomic dataset further explores gene regulation heterogeneity across broader cortical regions and subcortical regions. To discern regional diversity from other cell-type heterogeneities, we devised a workflow to unveil the regional landscape within single-nuclei DNA methylation profiles (Fig. 4A). Integrating these profiles with brain region data, we mapped the cells to a “regional methylation space” (Fig. 4A; Methods), where cells closer together have methylation neighbors from similar brain regions. In this “regional methylation space” (Fig. 4A; Methods), trajectories depict regional transitions alongside associated methylome shifts, thereby enhancing our grasp of regional DNA methylation effects.

Cortical excitatory neurons exhibited remarkable regional diversity in methylation, particularly the intratelencephalic-projecting neurons (LX-IT; Fig. 4B). The regional diversity of cortical inhibitory neurons (46) was less studied due to their inconspicuous regional patterns in transcriptome and epigenome (1, 47, 48). Our analysis reveals, though less pronounced, regional distinctions among cortical inhibitory neurons (Fig. 4B). Regional axes of each cortical neuronal cell type were constructed through single-cell trajectory analysis (49). We observed a shared ordering of brain regions on the axes among cortical neurons, from the posterior regions of the brain (like the primary visual cortex V1C) to the anterior lateral regions (like the prefrontal cortex A46 & the middle temporal gyrus MTG) and then to the anterior medial regions (like the anterior cingulate cortex ACC & the lateral entorhinal cortex LEC; Fig. 4, C and D). Only L6-CT showed an exceptional pattern (Fig. 4, B and C) from this Posterior–Lateroanterior–Medioanterior (P–LA–MA) trend. Nevertheless, the shared trend allowed for further analysis of a consensus regional axis for cortical neurons (Fig. 4C; Methods).

Epigenetic alterations along this axis suggest regional specification of cerebral cortices. For instance, the transcription factor NR2F1 (also known as COUP-TFI) has gradient expression during brain development, which is vital for establishing the caudal-rostral regional specialization in the neocortex (43) and the boundary between the neocortex and the entorhinal cortex (50). Our data showed low gene body methylation in V1C (P) and LEC (MA) and high in A46 (LA; Fig. 4G and fig. S16B), accompanied by a reversed trend of gene expression (fig. S16A). Two chromatin domains associated with NR2F1 showed interaction strengths changing in the opposite direction (Fig. 4F). In V1C, the upstream domain interacted more with NR2F1’s promoter and had hypo-methylated DMRs compared to LEC. In contrast, the downstream domain displayed a stronger interaction with NR2F1’s promoter and featured DMRs hypo-methylated in LEC (Fig. 4, F and G). Two neighbor genes NR2F1-AS1 and FAM172A, encompassed in these two domains respectively, showed concordant expression trends with the domain strengths (fig. S16A). Such coherent variations in epigenetics and transcription imply regulatory domain switching and alternative CRE usage to activate the same gene in different cortical regions, which needs further investigation.

Systematic examination of regionally differential epigenetic features in cortical neurons determined in total 14,606 (average 2.9k for each major type) regional DMGs (rDMGs), 885.4k (63.2k) regional DMRs (rDMRs), 773k (71.2k) regional differential loops (rDLs) and 1,495 (136) regional differential domain boundaries (rDB; fig. S16B; Methods). Many rDMGs and rDMRs showed monotonic methylation gradients along the P–LA–MA axis (Fig. 4E, and fig. S16, C and D), whereas more complex patterns (such as NR2F1) also existed.

Basal ganglia neurons exhibited remarkable regional diversity as well. An L-D-V axis (lateral to dorsal to ventral) became evident in the basal ganglia (Fig. 4H) with accompanying epigenetic shifts. For instance, moving from NAC through CaB to Pu, the LSAMP gene increased in mCH (Fig. 4, I and J) and decreased in strengths of chromatin domains and loops around (Fig. 4K). We determined 6,371 rDMGs and 398.8k rDMRs in the four major types of basal ganglia (MSN-D1, MSN-D2, FOXP2 and CHD7; fig. S16B), and identified 98,276 (50,271) rDLs and 193 (99) rDBs (fig. S16B) in MSN-D1 and MSN-D2 cells. The majority of rDMGs and rDMRs showed strong (anti-)correlations with the L–D–V axis (fig. S16, C to F), highlighting regional variation as a key to basal ganglia within-cell-type heterogeneity. Distinctions in both functions and neural connections between the dorsal (CaB and Pu) and the ventral parts of basal ganglia, particularly its major component striatum, have been noted previously (51, 52). Our data and analysis provided the epigenetic basis of the dorsal-ventral differences and refined the regional differences within the dorsal basal ganglia (Fig. 4H).

A considerable amount (427 out of 746) of TF motifs were enriched in rDMRs (fig S16G; Methods). Approximately 47% of these TFs are expressed in the corresponding cell types (fig. S16H), with expression (anti-)correlated with the regional axes (for example, fig. S16I). These findings hint at potential region-specific regulatory mechanisms in the brain, possibly underlying functional diversities.

## Conservation of brain cell types and DMRs between humans and mice.

**Figure 5.**
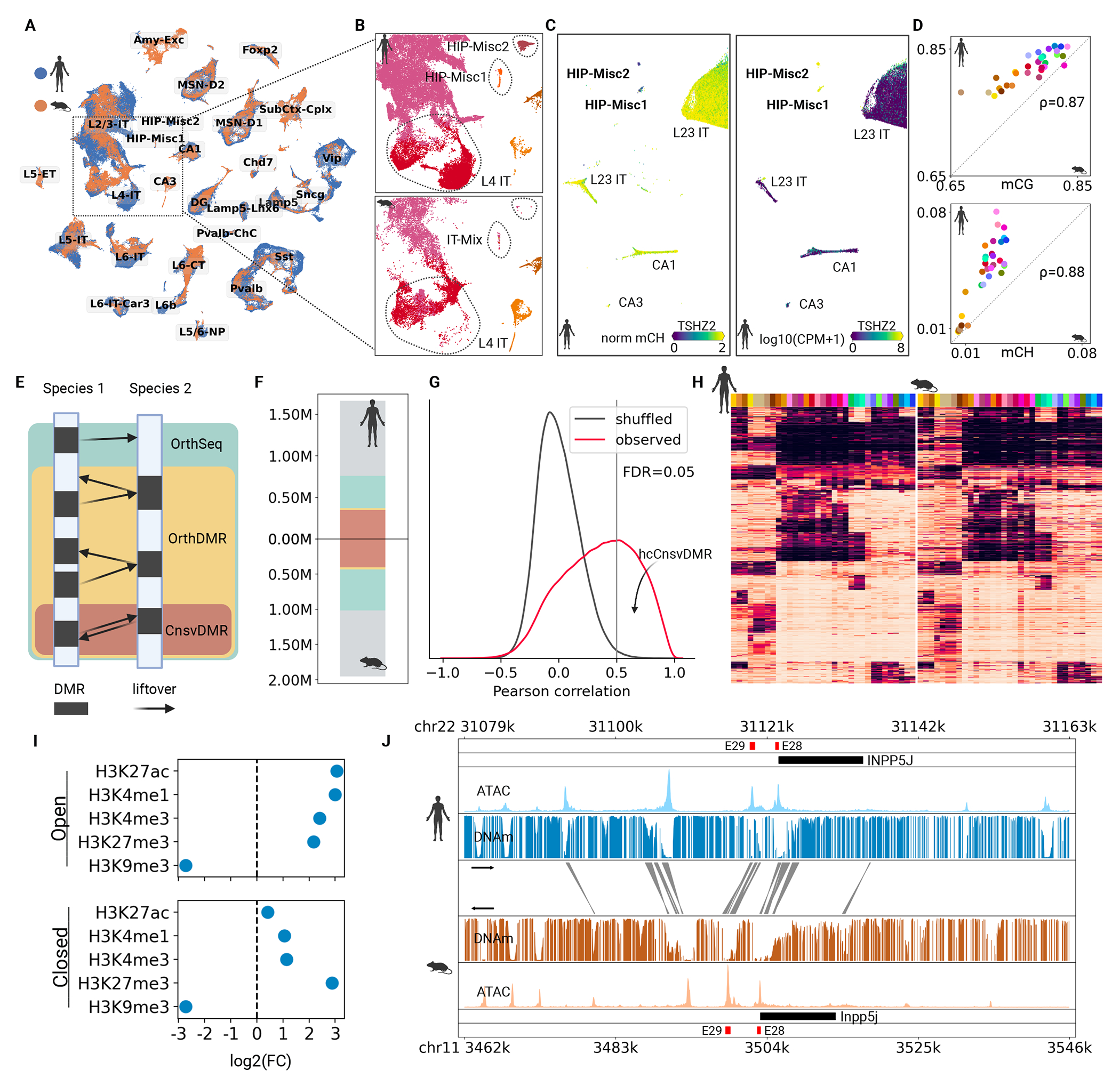
Cross-species comparison between human and mouse brain cell methylomes. (A) Integration of single-cell methylomes between human and mouse brains, visualized using 2D t-SNE. (B) Discrepancy between cell types of human and mouse brains in cell types L4-IT, HIP-Misc1, and HIP-Misc2. (C) CH-hypomethylation and gene expression of TF TSHZ2 in the cell types HIP-Misc1 and HIP-Misc2. (D) Correlated global mCH and mCG of conserved cell types between human and mouse. (E) Schematic of cross-species matching of cell type DMRs. (F) Overall, ~50% of DMRs have orthologous sequences in the other species, among which ~25% are reciprocal DMRs. (G) Distribution of cross-species correlation of DMR methylations (red) and the randomly shuffled background (black). (H) Examples of methylation fractions of hcCnsvDMRs. (I) The enrichment of the hcCnsvDMRs in the histone modification marks. (H) Browser view of hcCnsvDMRs around gene INPP5J in major type Pvalb. The regions colored by red are the cell type-specific distal enhancers validated in Ref (54).

Brain cell type conservation between primates and rodents was noted in several neocortical regions (17, 53). To assess whether the conservation holds in broader brain regions, we compared the single-nucleus DNA methylation profiles from human and mouse (1), using corresponding regions including the cerebral cortex, basal forebrain, basal nuclei, and hippocampus (Methods). The integration analysis showed three major types defined in human brains were discrepant with mouse brain cells (Fig 5A). Mouse L4-IT neurons aligned only to subpopulations of their human counterparts (Fig. 5B), confirming a larger heterogeneity in human L4-IT neurons (17). The human hippocampal HIP-Misc1 neurons were integrated with some mouse cortical IT neurons, and HIP-Misc2 neurons did not match any mouse cell type. The parallel snRNA dataset (11) validated these two human hippocampal cell types (Fig. 5C and fig. S17B). Although the unmatched cell types will need further investigation, the major type taxonomies were generally conserved across broader brain regions between humans and mice (Fig. 5A and fig. S17A), whereas both global CG- and CH-methylation were consistently higher in humans than in mice for corresponding cell types (Fig. 5D and fig. S17C).

To compare the gene regulation between human and mouse brains, we used liftOver to match major-type hypo-DMRs identified within single species (Fig. 5E). 40~60% hypo-DMRs across cell types had ortholog sequences in the other species (and we referred to these DMRs as OrthSeqs). Around half of OrthSeqs had their orthologs also hypo-DMRs in the other species (OrthDMRs). Most (95%) of OrthDMRs were reciprocally matchable (CnsvDMRs; Fig. 5F and fig. S17D). Methylation fractions of CnsvDMRs showed remarkable correlations across cell types between human and mouse (Fig. 5, G and H), suggesting functional conservation between species.

We further selected the most highly correlated DMRs (hcCnsvDMRs, Fig. 5G). Functional enrichment analysis of hcCnsvDMRs showed that they were enriched in biological processes related to forebrain development and in cellular components related to dendrites and synapses (fig. S17, F and G; Methods). Comparison to histone modifications in mouse forebrains (4) demonstrated these DMRs were depleted from heterochromatic regions (H3K9me3) as well as enriched in regions of enhancers (H3K27ac & H3K4me1), promoters (H3K4me3), and poised enhancers (H3K27me3; fig. S17E; Methods). Categorizing the hcCnsvDMRs further into open or closed status based on their chromatin accessibility (2) showed that open DMRs were enriched in enhancers and promoters. In contrast, closed DMRs were particularly enriched in the poised enhancers (Fig. 5I), which had probably been active during development.

Methylation conservation between species hints at a strategy for enhancer discovery through comparative epigenetics. For example, INPP5J, a specific gene of Pvalb neurons, had many distal and proximal hcCnsvDMRs overlapping with matched chromatin-accessible regions (Fig. 5J), including two validated as specific enhancers for viral targeting of mouse Pvalb neurons (Fig. 5J) (54).

## Single-cell methylation barcodes (scMCodes) reliably predict human brain cell Identity.

**Figure 6.**
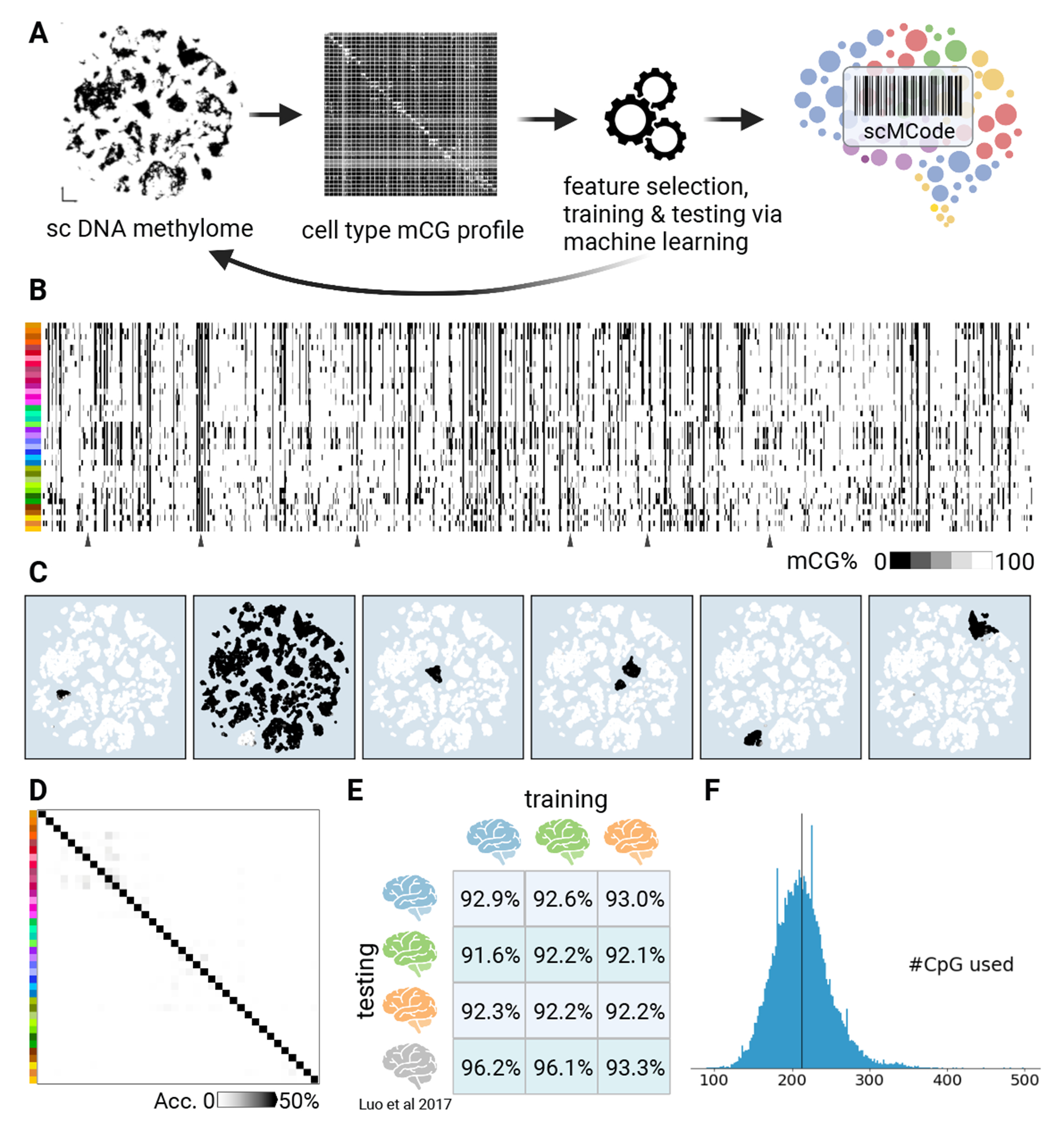
snMCodes for brain cell types. (A) Workflow of deriving snMCodes. (B) snMCodes derived from all three donors. (C) Examples of cell-type specificity of snMCode features. (D) Heatmap showing confusion matrix of snMCodes in predicting cell types. (E) Cell-type-prediction accuracy in cross-donor test. (F) snMCodes predict human cell types with a limited number of CpG sites at single-cell resolution.

DNA methylation variation in the genomes of cells contains molecular “engrams” representing past and present gene regulatory events (55). We observed distinct DNA methylation patterns on many CpG sites highly specific to brain cell types (for example, fig. S18B). This led us to devise single-cell methylation barcodes (scMCodes) to determine brain cell types at single cell level using the methylation status of selected CpG sites (Fig. 6A and fig. S18A; Methods).

We first selected CpG sites distinguishing brain cell types iteratively (Methods). These sites were further clustered into 39k groups according to their across-cell-type methylation patterns. We then assessed their cell-type predicting power through machine learning models with cross-validation (fig. S18A; Methods). 800 groups with a total of 12k CpG sites were selected as the scMCodes (Fig. 6B and C, and tables S8 and S9) to achieve good predicting power (Fig. 6D) while minimizing feature number (fig. S18C). These scMCodes achieved ~93% accuracy (Fig. 6D; Methods).

Cross-donor tests were conducted among the three donors of this study and an external individual (5). The results showed high prediction accuracies (92~96%; Fig. 6E), demonstrating the cross-individual robustness of the scMCode approach. Single-cell sequencing has limited genomic coverage. On average, only ~200 CpG sites of scMCodes were detected in each cell (Fig. 6F), which underscores the effectiveness of scMCode in determining human brain cell types using a few hundred select methylation sites.

## Discussion

A profound understanding of cellular diversity and distinctive gene regulatory mechanisms in the human brain is pivotal for elucidating brain functions and formulating therapeutics for brain disorders. We have compiled a comprehensive single-cell DNA methylation and 3D genome structure atlas of human brains with 524,010 deeply sequenced nuclei from 46 distinct brain regions, permitting us to identify 188 epigenetically distinct cell types. The extensive profiling of brain regions in this study has allowed us to identify cell types specific to subcortical regions and compare epigenetic diversity within the same cell type across different brain regions, which considerably expands previous work (3, 5, 56). Additionally, we have made considerable strides in understanding the 3D genome diversity across brain cell types and regions, facilitated by a 30-fold increase in cell profiling via snm3C-seq. Moreover, the specificity of domains and loops across 29 cell types was determined, pushing the cell type resolution extensively beyond previous studies (28, 57–59).

Single-nucleotide resolution DNAm has proven valuable in predicting epigenetic age (60), tracing cell lineage (61, 62), and diagnosing life-threatening diseases (63, 64). The intricate regulatory information encoded in DNAm has enabled us to distill a set of single-cell methylation barcodes (scMCodes) for reliable cell-type identification. Given that circulating-free DNA (cfDNA) methylation has been recognized as a robust tool for cancer diagnosis (58) and provided promising biomarkers for brain disorders (59), our scMCode method presents itself as a potentially transformative tool for the non-invasive diagnosis of brain disorders. It could aid in pinpointing pathological brain cell types and inform treatment selection, marking a stride forward in precision medicine.

However, several avenues warrant further exploration. 1) Beyond the 46 brain regions sampled in this study, the human brain has other intricate structures with complex cell diversity, particularly in subcortical regions. A more comprehensive brain region sampling, beyond what was available for this study, would provide deeper insights into the underlying gene regulation complexities. 2) Availability of high-quality tissues restricted us to only three male donors. Although this satisfied the purpose of this study for surveying human brain cell types and revealing their epigenomic patterns, expanding the donor base would further elucidate individual variations of brain cells, alongside the genetic impact on gene regulatory diversity. 3) Our findings largely stem from molecular modality correlations. Verifying these associations is imperative for delineating the functionality of regulatory elements, mapping regulatory networks, and harnessing putative enhancers for cell subtype studies.

Overall, this multimodal human brain cell atlas enriches our understanding of brain cells with a foundational epigenomic perspective. It offers not only an invaluable resource for exploring cell type diversity, gene regulation complexity, regional variation, and evolutionary conservation within brain cells but also provides the essential elements, such as putative regulatory elements, for the development of innovative genetic tools for cell type-specific targeting.

## Supplementary Material

Table 7

Table 6

Table 9

Table 8

Table 4

Table 2

Table 1

Table 5

Table 3

suppl info

## Data Availability

The data analyzed in this study were produced through the Brain Initiative Cell Census Network (BICCN:RRID:SCR_015820) and deposited in the NEMO Archive (RRID:SCR_002001) under identifier nemo:dat-jx4eu3g accessible at https://assets.nemoarchive.org/dat-jx4eu3g. Raw and processed data were also deposited to NCBI GEO/SRA with accession number GSE215353. A browser of single cell methylation can be found at https://cellxgene.cziscience.com/collections/fdebfda9-bb9a-4b4b-97e5-651097ea07b0. A summarization of data availability can be found at http://neomorph.salk.edu/hba/.
