# Supplementary material for: Single Cell DNA Methylation and 3D Genome Architecture in the Human Brain
Source: Science. Author manuscript; Available in PMC 2023 Oct 13. (PMC10572106; doi:10.1126/science.adf5357)
Supplement: suppl info [file NIHMS1929560-supplement-suppl_info.docx]

Supplementary Materials for

**Single Cell DNA Methylation and 3D Genome Architecture in the Human Brain**

Wei Tian, Jingtian Zhou, Anna Bartlett, Qiurui Zeng, Hanqing Liu, Rosa G. Castanon, Mia Kenworthy, Jordan Altshul, Cynthia Valadon , Andrew Aldridge, Joseph R. Nery, Huaming Chen, Jiaying Xu, Nicholas D. Johnson, Jacinta Lucero, Julia K. Osteen, Antonio Pinto-Duarte, Nora Emerson, Jon Rink, Jasper Lee, Yang Li, Kimberly Siletti, Michelle Liem, Naomi Claffey, Caz O'Connor, Anna Marie Yanny, Julie Nyhus, Nick Dee, Tamara Casper, Nadiya Shapovalova, Daniel Hirschstein, Song-Lin Ding, Rebecca Hodge, Boaz P. Levi, C. Dirk Keene, Sten Linnarsson, Ed Lein, Bing Ren, M. Margarita Behrens and Joseph R. Ecker

Corresponding author: Joseph R. Ecker ecker@salk.edu

**The PDF file includes:**

Materials and Methods

Supplementary Text

Figs. S1 to S18

References

**Other Supplementary Materials for this manuscript include the following:**

Tables S1 to S9

**Materials and Methods**

Human postmortem tissue specimen screening

These studies were intended to be the first explorations of cellular, transcriptional and epigenomic variation across the human brain using the latest single nucleus methylome (this study), RNA-seq, and ATAC-seq technologies. These methods perform best on tissue of the highest quality, prepared using methods optimized, which involved short postmortem interval (PMI targeting <12 hours), highly consistent tissue slabbing and photo documentation to ensure anatomically precise sampling, freezing with supercooled isopentane to preserve tissue integrity, and proper storage under vacuum in -80˚C freezers. In addition to low PMI, stringent exclusionary criteria were applied for RNA integrity (RIN, >7.0), infectious diseases, head trauma, intubation, neuropathology and manner of death.

The availability of tissues was a significant challenge given the highly stringent exclusionary criteria. Brain specimens meeting these criteria, and for which whole brain hemispheres could be obtained for the current study, were quite rare and with a heavy male bias. Between 2018-2022 16 donors met these criteria, with only three female donors who ultimately were excluded based on QC or other exclusionary criteria. The three donors passing all exclusionary and QC criteria were all males.

Human postmortem tissue specimen processing

De-identified adult postmortem human brain tissue was obtained after receiving permission from the deceased’s next of kin. Tissue collection was performed per the United States Uniform Anatomical Gift Act of 2006, described in the California Health and Safety Code section 7150 (effective 1/1/2008) and other applicable state and federal laws and regulations. In addition, the Western Institutional Review Board reviewed tissue collection procedures and determined that they did not constitute human subjects research requiring institutional review board (IRB) review.

Male donors 18–68 years of age with no known history of neuropsychiatric or neurological conditions were considered for inclusion in the study. Routine serological screening for infectious diseases (HIV, Hepatitis B, and Hepatitis C) was conducted using donor blood samples, and donors testing positive for infectious diseases were excluded from the study. Specimens were screened for RNA quality, and samples with average RNA integrity (RIN) values ≥7.0 were considered for inclusion in the study. Postmortem brain specimens were processed as previously described [(*17*)](https://paperpile.com/c/cCKYZi/IgzFn)(dx.doi.org/10.17504/protocols.io.bf4ajqse). Briefly, coronal brain slabs were cut at 1 cm intervals, photographed, frozen in dry-ice cooled isopentane, and transferred to vacuum-sealed bags for storage at -80°C until the time of further use. For the dissection of brain regions of interest, photos of tissue slabs were annotated by a neuroanatomist to outline regions to target for dissections. Then, tissue slabs were removed from the -80°C freezer and briefly transferred to -20°C, where they were held for ~1-3 hours to allow tissues to equilibrate to -20°C. Tissues were then transferred to a custom temperature-controlled cold table held at -20°C and the region of interest was removed using standard razor blades or scalpels. Tissue blocks were stored at -80°C in vacuum-sealed bags until later use.

Nuclei isolation and Fluorescence Activated Nuclei Sorting (FANS)

Nucleus isolation was conducted using a standard protocol as previously described (dx.doi.org/10.17504/protocols.io.y6rfzd6). Gating on DAPI and NeuN fluorescence intensity was as described previously [(*17*)](https://paperpile.com/c/cCKYZi/IgzFn). NeuN+ and NeuN- nuclei were sorted into separate tubes and were pooled at a defined ratio of 90% NeuN+ and 10% NeuN- nuclei after sorting. Sorted samples were centrifuged, frozen in a solution of 1X PBS, 1% BSA, 10% DMSO, and 0.5% RNAsin Plus RNase inhibitor (Promega, N2611), and stored at -80°C until further processing. The presorted nuclei pellets were defrosted and resuspended in DPBS+1%BSA, centrifuged, resuspended back in 1ml of DPBS, and sorted into 384-well plates. Nuclei from donors H19.30.001 and H19.30.002 were prepared and sorted into 384-well plates. For donor H19.30.004, frozen tissue blocks received from AIBS were processed following procedures previously described [(*7*)](https://paperpile.com/c/cCKYZi/8tPb1). Nuclei were labeled for NeuN fluorescence and sorted into 384-well plates as described [(*1*)](https://paperpile.com/c/cCKYZi/x536h).

Library preparation and Illumina sequencing

**snmC-seq library preparation.** snmC-seq3 libraries were prepared using an updated version of snmC-seq2. In brief, samples underwent bisulfite conversion and were barcoded with random primers. Samples were then pooled through two SPRI cleanups to compress 16 x 384-well plates into 1 x 96-well plates. Pooled samples were then adapted and amplified as previously described. Next, libraries were pooled and cleaned through two more SPRI cleanups. Finally, library concentrations were determined by Qubit and normalized for sequencing. snmC-seq3 and snm3C-seq (see below) libraries generated from human brain tissues were sequenced using an Illumina Novaseq 6000 instrument with S4 flowcells and 150 bp paired-end mode.

**snm3C-seq library preparation.** For some samples from donors H19.30.001 and H19.30.002, presorted nuclei were used. The presorted nuclei pellets were defrosted and resuspended in DPBS+1%BSA, centrifuged, and resuspended back in 1 ml of DPBS. For the remaining samples of donors H19.30.001 and H19.30.002 and all samples from donor H19.30.004, frozen tissue was pulverized using a mortar and pestle. All samples were then immediately crosslinked with 2% formaldehyde in solution for 5 min, quenched with 0.2M Glycine for 5 min, centrifuged and washed with DPBS, and stored at -80°C until ready for further processing. Next, nuclei were conditioned and digested using an Arima kit adapted for snm3C-seq for 1hr at 37°C, and 20 min at 65°C to inactivate enzymes, then ligated for 15min at room temperature. Finally, nuclei were resuspended in 1ml of DPBS+1%BSA, filtered through a 0.2 2µM filter, and sorted similarly to the snmC-seq3 samples.

The detailed protocols for snmC/snm3C-seq were described here dx.doi.org/10.5281/zenodo.8319891

Donor-specific genomes

**gDNA Library prep protocol.** Genomic DNA was extracted from ground, frozen tissue using the DNeasy Blood and Tissue Kit (Qiagen, Valencia, CA). One µg of DNA was fragmented with a Covaris S2 (Covaris, Woburn, MA) to 300 bp, followed by end repair (Lucigen) and the addition of a 3’ A base (New England Biolabs). Cytosine-methylated adapters provided by Illumina (Illumina, SanDiego, CA) we ligated to the sonicated DNA at 16˚C for 16 hours with T4 DNA ligase (New England Biolabs). Adapter-ligated DNA was isolated by two rounds of purification with AMPure X P beads (Beckman Coulter Genomics, Danvers, MA). The adapter-ligated DNA molecules were enriched by 4 cycles of PCR with the following reaction composition: 25µL of Kapa HiFi Hotstart (KapaBiosystems, Woburn, MA) and 5µl TruSeq PCR Primer Mix (Illumina) (50µlfinal). The thermocycling parameters were: 95˚C 2min, 98˚C 30sec, then 4cycles of 98˚C 15 sec, 60˚C 30 sec, and 72˚C 1min, ending with one 72˚C 5 min step. The reaction products were purified using AMPure X P beads. The purified PCR reactions of the adapter-ligation resulted in a library used for subsequent sequencing in Novaseq 6000.

**Variant calling from donor genome sequencing.** Whole genome sequencing reads were first QCed with the software fastp (v0.20.1) [(*65*)](https://paperpile.com/c/cCKYZi/ZNi6u). The command line used is “fastp -i input_PE_R1.fastq.gz -I input_PE_R2.fastq.gz -o output_PE_R1.fastq.gz -O output_PE_R2.fastq.gz -w 4”. The QCed reads were then mapped to human genome assembly GRCh38 (hg38) via the software BWA (v0.7.17)[(*66*)](https://paperpile.com/c/cCKYZi/LYLEX) with the BWA-MEM algorithm with mapping results stored in bam format through the software samtools (v1.10)[(*67*)](https://paperpile.com/c/cCKYZi/4UOkZ). Specifically, the command line used for mapping is “bwa mem -t 20 hg38-ref input_PE_R1.fastq.gz input_PE_R2.fastq.gz | samtools view -Sb - > output.bam”.

The mapped reads were analyzed with the germline short variant discovery workflow of the Genome Analysis Toolkit (GATK, v4.1.8.1)[(*68*)](https://paperpile.com/c/cCKYZi/PwgmZ). Briefly, we first removed the duplicated reads from the mapped reads, which then went through a base quality score recalibration step (BQSR) to generate analysis-ready reads. The variant references used in the BQSR step were dbSNP138, Mills and 1000 Genomes gold standard indels, and 1000 Genomes phase 1 SNPs. Next, candidate variants (SNPs+InDels) were called with the HaplotypeCaller of GATK from the analysis-ready reads and further filtered with a variant quality score recalibration step (VQSR) to determine the high-confidence SNPs and InDels, respectively. The variant references used to recalibrate SNP quality scores were Hapmap 3.3, OMNI 2.5, 1000 Genomes phase 1 and dbSNP138, and of InDels were Mills and 1000 Genomes gold standard indels and dbSNP138. All the references used in the BQSR and VQSR were downloaded from the GATK resource bundle (ftp://ftp.broadinstitute.org/bundle/hg38).

**Donor-specific reference genome.** For each donor, we selected the high-confidence homozygous SNPs using the function SelectVariants of GATK, and created donor-specific reference genomes by substituting the homozygous SNPs into the hg38 FASTA file using the function FastaAlternateReferenceMaker of GATK

**Common homozygous SNPs.** By comparing the homozygous SNPs of donors, we constructed a list of common SNPs shared among the three donors.

Mapping and count/feature matrix generation

For sequence read mapping of both snmC-seq3 and snm3C-seq datasets, we used our own custom pipeline (https://github.com/lhqing/cemba_data, version 1.2.1.dev94+gc65e173). The main steps of this pipeline included: 1) Demultiplexing FASTQ files into single-cell; 2) Reads level QC; 3) Mapping; 4) BAM file processing and QC; 5) final molecular profile generation. The details of the five steps were previously described [(*10*)](https://paperpile.com/c/cCKYZi/DN4Wq). We mapped all of the reads to the donor-specific genomes. After mapping, we calculated the methylcytosine counts and total cytosine counts for two sets of genomic features in each cell. Non-overlapping chromosome 100kb bins of the hg38 genome (generated by “bedtools makewindows -w 100000”), were used for clustering analysis, and the genes defined by the human GENCODE v33 were used for cluster annotation and integration with datasets. Both CG and CH methylation levels of the features were normalized as previously described [(*1*)](https://paperpile.com/c/cCKYZi/x536h). The cell-by-feature matrices were generated from normalized methylation levels of each feature set.

Quality control measures

The sequenced cells were filtered based on these metrics: 1) mCCC% < 0.06; 2) global mCG% > 0.5; 3) global mCH% < 0.15; 4) total final reads > 250,000; 5) mapping rate > 0.5. For cells profiled with snm3C-seq, we required a cell to have > 50,000 cis contacts with a distance over 2500bp.

Clustering and annotation of snmC-seq3 data

**Clustering analysis.** CG- and CH-methylation levels of 100kb genomic bins were used as input features for clustering. We performed clustering analysis iteratively using the software package ALLCools (<https://github.com/lhqing/ALLCools>). In each iteration, the 100kb bins were first filtered by removing bins with mean total cytosine base calls < 250 or > 3000. Those who overlap with the ENCODE blacklist [(*69*)](https://paperpile.com/c/cCKYZi/vJLD5) were also excluded from the clustering analysis. The Top 5,000 highly variable features (HVFs) were then selected separately from both CG- and CH-methylation via support vector regression (SVR). We then applied principle component analysis (PCA) to each 5,000 features to reduce dimension. The top *n* principle components (PCs) were selected for each methylation type until there is no significant difference between the distributions of *n*-th and *(n+1)*-th PCs by two-sample Kolmogorov-Smirnov test with the criteria as the adjusted p-values < 0.1. We further performed pre-clustering for each top PC set and selected the PCs that are enriched in pre-clusters (adjusted p-values<=0.05). Finally, the selected PCs from both CG and CH methylation PCs were concatenated for further analysis. We used Harmony [(*70*)](https://paperpile.com/c/cCKYZi/FesJa) on the selected PCs in order to eliminate individual differences. The Harmonized features were further fed into the consensus clustering procedures previously described [(*1*)](https://paperpile.com/c/cCKYZi/x536h).

**Doublet/debris identification.** The read number of each cell in one plate is stable in both snmC-seq3 and snm3C-seq. Therefore we adopted a doublet/debris detection strategy based on cell relative reads to its plate. We first normalized the read number per cell to the mean reads of its plate. The cells with plate-relative-read numbers>1.2 or <0.8 were considered doublet/debris candidates. After each iteration of clustering, clusters would be labeled as doublet/debris if the cluster contained over 80% doublet/debris candidates and were eliminated from further analysis.

**Cell type annotation.** The clusters were manually annotated as major or subtypes according to their hypomethylated genes, which were either canonical brain cell type markers or determined *de novo* from the current dataset. We required each cell type to have at least five differentially methylated genes in CG and CH methylation compared to the other cell types. Otherwise, it would be merged with the closest cluster. A candidate cell type would be labeled as an outlier if all its cells were from a single donor.

In major type level, where possible, cell types were annotated using the nomenclature for known brain cell types previously described in the literature(e.g. (13)); otherwise, cell clusters were annotated according to either the regional composition or distinct marker genes of the cell type. One caveat of the former approach is that cell types annotated using marker genes defined in rodents or non-human primates might not reflect the corresponding gene activity in human cell types. For example, the gene SNCG is lowly expressed in the human major type corresponding to mouse Sncg cells. Nevertheless, using common nomenclature aids in cross-species comparison and existing knowledge transferring.

Clustering and annotation of snm3C-seq data

To annotate cells from snm3C-seq, we combined them with the annotated snmC-seq3 cells and carried out an iterative clustering analysis similar to what was described above. The only difference was that batch effects from both individuals and sequencing technologies were corrected using the software Scanorama [(*71*)](https://paperpile.com/c/cCKYZi/JFCrQ). After each clustering iteration, the cell type annotations were transferred from snmC-seq3 cells to snm3C-seq cells with a K Nearest Neighbor (KNN) classifier.

Robust dendrogram of cell types

We resampled a certain number of cells from each cell type without replacement to compute the average methylome profile for the cell type with genome features of 100kb-bins of both CG- and CH-methylation. The resampling number is 800 for major types and 500 for subtypes. The average profiles were then used to compute the pairwise correlation distances. This process was repeated 500 times to compute an average pairwise distance matrix, which was then used to construct the final cell-type dendrogram via hierarchical clustering with average linkage.

Determine differentially methylated genes

We determined the DMGs pairwise between cell subtypes for CG- and CH-methylation separately. To avoid potential bias caused by an imbalance of cell numbers of cell types, we downsampled cells in each cell subtype to no more than 500. All the protein-coding and long non-coding RNA genes (lncRNAs) defined by the human GENCODE v33 were tested for significant methylation decrease (or hypomethylation) using the Wilcoxon rank-sum test. The p-values were adjusted with multitest correction using the Benjamini-Hochberg procedure. We computed the Area Under the Receiver Operating Characteristic curve (AUROC) for the candidate genes. The genes with adjusted p-values≤0.001 and AUROC≥0.8 were considered pairwise DMGs in CG- and CH- methylation.

Determine differentially methylated regions

We merged single-cell DNA methylation profiles into the cell type (major type/subtype) profiles according to their cluster annotation in both donor-aggregated and donor-separated ways. Non-common homozygous SNP CpG sites of these methylation profiles were filtered out before further analysis. We then used the DMRfind function of the software MethylPy (v1.4.2; [(*72*)](https://paperpile.com/c/cCKYZi/ed4os) ) to determine the mCG DMRs across all cell types of the donor-aggregated profiles. The command line used is “methylpy DMRfind --output-prefix OUTPUT_FILE_NAME --samples SAMPLE_NAMES --mc-type CGN --dmr-max-dist 250 --sig-cutoff 0.01 --allc-files MC_FILES”. We further merged the successive DMRs if their distance is within 250bp and the Pearson correlation of their mCG fractions across 188 subtypes is greater than 0.8. We further screened each DMR by evaluating the reproducibility of the methylation pattern across cell types between donor-aggregated and -separated profiles. The evaluation criteria were 1) the Pearson’s correlation coefficient between the mCG fractions across cell types is ≥0.5, and 2) the mean-absolute-error (MAE) is ≤0.1.

Each reproducible DMR was then assigned as hypo- or hyper-DMRs in each cell type based on the difference of its mCG fraction from its robust mean. The robust-mean m of each DMR was calculated by averaging the mCG fractions between 25th and 75th percentiles across cell types. The DMRs with mCG fractions greater than m+0.3 were assigned as the hyper-DMRs in each cell type, and lower than m-0.3 were assigned as hypo-DMRs. DMRs containing only 1 CG site or without any hypo- or hyper-DMR assignment were excluded from further analyses.

Motif enrichment analysis

746 transcription factor binding profiles (motif) from JASPAR2020 [(*73*)](https://paperpile.com/c/cCKYZi/rYUaz) were used to perform the motif enrichment analysis. Cell-type-specific hypo-DMRs were first segmented into 500bp bins, and then annotated with each motif by intersecting with the genome locations of the motifs. Motif genome locations were downloaded from http://expdata.cmmt.ubc.ca/JASPAR/downloads/UCSC_tracks/2020/hg38. To test for motif enrichment in the major cell types, hypo-DMRs were used as foreground signals, and the hypo-DMRs of all other major types were used as background. For testing at the cell subtype level, hypo-DMRs in only the other subtypes that belong to the same major type were used as background. The one-sided Fisher exact test was used to calculate the p-values of enrichment of the foreground against the background.

Integration among different single-cell datasets

**Feature matrices for human single-cell DNA methylation, expression and open chromatin.** CG- and CH-cell type marker genes determined from the mC dataset for both major and subtype levels were used as the features for integration analysis. When integrating with scRNA (companion manuscript Siletti et al. [(*11*)](https://paperpile.com/c/cCKYZi/xrySe)) or snATAC datasets (companion manuscript Li et al.), we used the opposite values of gene body methylation since they generally are strongly anti-correlated with gene expression. Both scRNA and snATAC datasets were normalized by the averaged total UMI counts of the featured genes and then transformed by *log(x+1)*. The neuronal cell types and non-neuronal cell types were integrated separately. CH-methylation was used for neuronal cell types, while CG-methylation was used for non-neuronal cell types. An additional filtering step was applied before integrating non-neuronal cell types, which required the total UMI of the featured genes of each cell to be larger than 3,000 for both scRNA and snATAC datasets.

**Feature matrices for human and mouse single-cell DNA methylation.** We used only homologous genes between human and mouse to perform the integration analysis. The list of homologous genes was downloaded from the Mouse Genome Informatics (MGI) database (<http://www.informatics.jax.org/homology.shtml>). The homologous genes were selected from the same features used when integrating with scRNA and snATAC datasets. Human brain cells from thalamus, midbrain, cerebellum, pons, and entorhinal cortices were excluded since no counterparts exist from the public mouse dataset [(*1*)](https://paperpile.com/c/cCKYZi/x536h). The mouse dataset was re-annotated in the same way as the human dataset. CG-methylation was used to integrate neuronal and non-neuronal cell types separately.

**Method to integrate different single-cell sequencing datasets.** After feature matrix generation, we used a 3-step method analogous to Seurat v3 to project two datasets $X$ and $Y$ onto the same space: 1) Using canonical correlation analysis (CCA) to capture the shared variance across cells between datasets; 2) finding anchors as 5 mutual nearest neighbors (MNN) between the two datasets; 3) pulling the two datasets into the same space. To allow the scalability, we randomly selected 20,000 cells from each dataset ($X_{ref}$ and $Y_{ref}$) as a reference to fit the CCA, and transform the other cells ($X_{qry}$ and $Y_{qry}$) onto the same CC space. Specifically, the canonical correlation vectors (CCV) of $X_{ref}$ and $Y_{ref}$ (denoted as $U_{ref}$ and $V_{ref}$) are computed by singular value decomposition on their dot product, $U_{ref}SV_{ref}^{T}=X_{ref}Y_{ref}^{T}$, where $U_{ref}^{T}U_{ref}=I$ and $V_{ref}^{T}V_{ref}=I$. Then the CCV of $X_{qry}$ and $Y_{qry}$ (denoted as $U_{qry}$ and $V_{qry}$) are computed by $U_{qry}=X_{qry}{(Y}_{ref}^{T}V_{ref})/S$ and $V_{qry}=Y_{qry}{(X}_{ref}^{T}U_{ref}^{T})$. U and V were normalized by dividing the L2-norm of each row, and used to find MNN anchors and score anchors using the same method as Seurat v3. $X$ and $Y$ were also combined vertically and the PCs of this combined matrix were integrated together using the same method as Seurat v3 through the anchors generated from the previous step. This integration step projects the PCs of one dataset (query) to the PCs of the other dataset (reference) while keeping the PCs of the reference dataset unchanged. The resulting PCs were used for visualization and finding matched clusters between datasets.

3D genome analysis

The 3D genome features were analyzed at both single-cell and pseudobulk levels. For single-cell analysis, we used scHiCluster [(*15*)](https://paperpile.com/c/cCKYZi/mRpsU) to impute the contact matrices at 100kb resolution with pad = 1, 25kb resolution with pad = 1 for contact within 10.05 Mb, 10kb resolution with pad = 2 for contact within 5.05 Mb. To speed up the imputation at 10kb resolution, the convolution and random walk were performed within each 30 Mb sliding window across each chromosome with a step size of 10 Mb. Only the values within the 10 Mb in the center of the sliding window were used as the final result.

For pseudobulk analysis, we merged the cells from each group (major type, subtype, or region) by taking the sum of raw matrices or the average of imputed matrices over cells within the group, and only randomly selected 1500 cells to use if the group contained more than 1500 cells. We also had a group with merged all cell types used in the compartment analysis and embedding comparison. This group contains 5707 cells in total, generated by randomly selected 200 cells with >=100,000 contacts from each major type, except for L5-ET where only 107 cells were identified and all used in the analysis. The details of methods are described below.

Contact distance distribution analysis

We generated a histogram of contacts for each single cell based on the distance between the two anchors of the contact. The bins are equally divided on the log2 distance scale, with a step size of 0.125, ranging from 2500 bp to 249 Mb (length of the longest chromosome). The i-th bin is the number of contacts with a distance between $2500\times2^{0.125i}$ and $2500\times2^{0.125(i+1)}$. In Figs. 2B and S5, the short-long ratio was defined as the proportion of contacts in 51st (200k) to 76th (2M) bins divided by the proportion of contacts in 103rd (20M) to 114th (50M) bins.

Compartment analysis

Pseudo-bulk contact matrices of each chromosome at 100kb resolution were used for compartment analysis. We first used the merged contact maps of the 5707 cells and filtered out the 100kb bins with abnormal coverage. Specifically, the coverage of bin i on chromosome c (denoted as R_c,i_) was defined as the sum of the i-th row of the contact matrix of chromosome c. We only kept the bins with coverage between the 99th percentile of R_c_ and twice the median of R_c_ minus the 99th percentile of R_c_. Contact matrices were normalized by distance, and Pearson's correlation matrices of the normalized matrices were computed. These merged contact maps were used to fit the principal component analysis (PCA) models per chromosome. The first principal components (PC1) were used as compartment scores, and the sign of the model was adjusted to ensure the compartment with higher CpG density had positive scores. We visually inspected PC1 of the merged matrices to ensure the values correspond to the plaid pattern of the correlation matrix rather than chromosome arms. The contact maps of each major type were filtered and converted to the correlation matrices in the same way as described above and were then transformed with the PCA models. Both raw matrices and imputed matrices were used for this analysis. We used the merged raw matrices for fitting the PCA model, and transformed the correlation matrices of raw matrices in each cell type as raw compartment scores, and transformed the correlation matrices of imputed matrices in each cell type as imputed compartment scores. In general, the imputed matrices work better with smaller cell populations, while the raw matrices provide higher resolution when enough cells are merged.

To examine whether the enriched longer-range interactions are inter- or intra- compartment, we stratified the contact distance plot by the difference or summation of compartment scores at the contact anchors. The difference of the scores reflected whether the contact was intra- or inter-compartment, and the larger difference represented inter-compartment. The summation of the scores distinguished whether the contact was AA or BB for intra-compartment contacts, and the large positive summation represents AA interaction whereas the small negative summation represents BB interaction. Note that longer-range contained more inter-compartment interactions than shorter-range in general, so non-neuronal cells which had more longer-range interactions also had more inter-compartment contacts than neurons when counting the raw contact counts. The results we reported in fig. S5, F to K used distance normalized contact counts, which reflected a relative proportion of intra- or inter-compartment contacts at each distance. Therefore, our results only suggested that a higher proportion of longer-range contacts in non-neuronal cells were intra-compartment, which did not indicate that there were more intra-compartment contacts in total in non-neuronal cells.

Saddle plots and compartment strengths are computed in the same way as described in [(*74*)](https://paperpile.com/c/cCKYZi/rsmwd). Specifically, within each chromosome, we rank all the 100kb bins based on compartment scores, and group the bins into 50 equal-interval groups. The distance normalized interaction strength between each pair of bins, or the PCC of mCG or mCH levels between each pair of bins were averaged within each group. The axes are ranked by the compartment score of the cell types so that BB interactions are on the top left and AA interactions are on the bottom right.

Differential compartments (DCs) were identified with dcHiC [(*75*)](https://paperpile.com/c/cCKYZi/ddZhh) between all major types or neuronal major types using the raw compartment scores as input. A large proportion (>60%) of bins were identified as differential with a traditional q-value threshold of 0.01. Therefore, we only selected the top DCs with a Z-score transformed Mahalanobis distance >1.960 (97.5 percentile of standard normal distribution).

Identification of domains and differential domain boundaries

Domains and insulation scores were derived with scHiCluster at 25kb resolution. Specifically, domains were identified within each single cell with TopDom [(*76*)](https://paperpile.com/c/cCKYZi/VoD6N) on the imputed matrices at 25kb resolution. Insulation scores were computed in each cell group (major type or major type within a brain region) for each bin with the pseudo-bulk imputed matrices (average over single cells) and a window size of 10 bins. The boundary probability of a bin is defined as the proportion of cells having the bin called as a domain boundary among the total number of cells from the group.

The number of domains identified in single cells is correlated with the number of short-range reads which could affect the performance of imputation. To avoid computational artifacts, we selected the cells from each cell type to match the distribution of short-range contacts across cell types and observed the same trend in fig. S6, D and E, which suggests these differences between domain numbers and sizes are not completely explained by the different short/long ratios between neurons and non-neurons.

To identify differential domain boundaries between n cell groups, we derived an nx2 contingency table for each 25kb bin, where the values in each row represent the number of cells from the group that has the bin called as a boundary or not as a boundary. We computed the Chi-square statistic and p-value of each bin and used the peaks of the statistics across the genome as differential boundaries. The peaks are defined as a local maximum of Chi-square statistics within FDR <1e-3 (Benjamini and Hochberg procedure). If two peaks are within 5 bins of each other, we only keep the peak with a higher Chi-Square statistic. We also require the peaks to have a Z-score transformed Chi-square statistic >1.960 (97.5 percentile of standard normal distribution), and differences between maximum and minimum boundary probability >0.05.

Identification of chromatin loops and differential loops

Chromatin loops were identified with scHiCluster [(*15*)](https://paperpile.com/c/cCKYZi/mRpsU) in each major type, subtype, and major type within each brain region, respectively. We only perform loop calling between 50 kb and 5 Mb, given that increasing the distance only leads to a limited increase in the number of significant loops. For each single cell, the imputed matrix of each chromosome Q_cell_ was log-transformed, and Z-score normalized at each diagonal (result denoted as E_cell_) and subtracted a local background between >=30 kb and <=50 kb (result denoted as T_cell_), similar to SnapHiC [(*77*)](https://paperpile.com/c/cCKYZi/aLTg0). A pseudo-bulk level t-statistic was computed to quantify the deviation of E and T from 0 across single cells from the cell group, where larger deviations represent higher enrichment against global (E) or local (T) background. E_cell_ is also shuffled across each diagonal to generate E_shufflecell_, and then T_shufflecell_, to estimate a background of the t-statistics. An empirical FDR can be derived by comparing the t-statistics of observed cells versus shuffled cells. We required the pixels to have an average E >0, fold change >1.33 against donut and bottom left backgrounds, fold change >1.2 against horizontal and vertical backgrounds [(*77*)](https://paperpile.com/c/cCKYZi/aLTg0), and FDR <0.01 compared to global (E) and local (T) backgrounds. The loop summits were selected from the loop pixels with a breadth-first search algorithm, where we started from the loop pixels with the largest E, and connected it with all the other loop pixels within 20kb (L0 distance) with smaller E values. The loop pixel with the largest E value in each connected component of loop pixels was defined as a loop summit. We only used the concept of summit during the counting of loop summits, and in all other cases, we used “loop” to represent loop pixels.

To compare the interaction strength of loops between different groups of cells, we adopt an analysis of variance (ANOVA) framework to compute the F statistics for each loop identified in at least one cell group using either Q_cell_(result denoted as F_Q_) or T_cell_ (result denoted as F_T_). Then, we Z-scored F_Q_ and F_T_ across all the loops being tested and selected the ones with F_Q_ and F_T_ > 1.036 (85th percentile of standard normal distribution) as differential loops. The threshold was decided by visually inspecting the contact maps as well as the correlation of interaction and loop anchor CG methylation.

Motif enrichment analysis between differential loops and constant loops was carried out after controlling the interaction strength and the enrichment against the local background. We first generated a pool of constant loops whose Z-scored F_Q_ and F_T_ < 0. Then we grouped the differential and constant loops into 100 x 100 groups based on F_Q_ and F_T_. We selected the same number of loops from each group for differential and constant loops and compared the motif enrichment in the differential loops or constant loops compared to the union of them.

Differential loops were identified in 11 comparisons, including between all major types; between neuronal major types; between neuron, glia (ASC, ODC, OPC), MGC, PC, EC, VLMC; between glial major types; between excitatory neurons (L2/3-IT, L4-IT, L5-IT, L6-IT, L6-IT-Car3, L5/6-NP, L6-CT, L6b, L5-ET, Amy-Exc), inhibitory neurons (Lamp5, Lamp5-Lhx6, Sncg, Vip, Pvalb, Pvalb-ChC, Sst, Chd7), cerebral nucleus neurons (MSN-D1, MSN-D2, Foxp2), and SubCtx-Cplx; between excitatory major types; between inhibitory major types; between cerebral nucleus major types; between intra-telencephalic (IT) major types (L2/3-IT, L4-IT, L5-IT, L6-IT); between Caudal ganglionic eminence (CGE)-derived major types (Lamp5, Lamp5-Lhx6, Sncg, Vip); between Medium spiny neurons major types (MSN-D1, MSN-D2). The results can be found in table S6. Aggregate peak analysis (APA) of some of the comparisons is shown in fig. S8. For each single loop pixel, the imputed contact map from -100kb to +100kb was selected and min-max normalized to the range of 0 to 1, and averaged across all the differential loops that have a folder change of Q and T greater than 1.2 and 1.5, respectively, comparing the average of foreground cell types and the average of background cell types.

Single-cell embedding based on different 3D genome features

**Contact based.** The imputed contacts at 100kb resolution with distance >=100kb and <=1 Mb are used as features for singular value decomposition (SVD) dimension reduction. The first 30 principal components were normalized by singular values and L2 norms per cell and then used for t-SNE visualization in Fig. 1G and S2F. To better visualize the heterogeneity of neuronal cells, we downsample each of the neuronal cell populations to 1,000 cells and, together with all the neurons to fit the t-SNE, and project back the other non-neuronal cells. Higashi [(*20*)](https://paperpile.com/c/cCKYZi/QH3HP) and fastHigashi [(*21*)](https://paperpile.com/c/cCKYZi/4cLiX) were used in the comparison of embedding in fig. S7. Due to the memory limitation (256G), we only run these tools at 500kb resolution rather than 100kb (same as suggested in their papers and github pages). The donor information was used as a confounding factor in the models to avoid the embedding being driven by donor differences.

**Compartment based.** Single-cell compartment scores were computed using either the CpG density method or the eigenvector method on raw contact maps or contact maps imputed by scHiCluster or Higashi. CpG method used the CpG density of each 100kb bins across the genome, and a value for each bin was computed for each single cell as the average of CpG density of other 100kb bins on the same chromosome, weighted by the interaction strength between the two bins. The eigenvector method used an average of imputed matrices across all single cells to compute the correlation matrices (as described in the compartment analysis) and fit PCA models to transform the correlation matrices of all single cells. Higashi was also used to impute the contact maps after generating the cell embedding as described in “Single-cell embedding based on chromatin contacts”, using either 0 neighbors or 5 neighbors on the embedding space to help imputation. Although using 5 neighbors generated compartments with higher cell type specificity, this method enforced the smoothing of information on the cell embedding, which could artificially augment the difference between imputed matrices when the embedding can separate the cell types well. Therefore, this makes it challenging to claim if the separation of cell types is due to the intrinsic heterogeneity of compartments across single cells or due to the smoothing of the embedding and we still show the result with 0 neighbors in Fig. S7. We then performed SVD on the cell-by-bin compartment score matrix $X=USV^{T}$ to derive the cell embedding $U$.

**Loop based.** We combined the loop pixels identified in all major types to make a meta loop list and generated a binary cell-by-loop matrix where each element indicated whether a contact was detected in the cell at the loop pixel. Latent semantic analysis with log term frequency was applied to the binary matrix (denoted as $A$) to compute the embedding. Specifically, we selected the columns having 1 in more than 5 rows, then computed the column sum of the matrix (${colsum}_{j}=\sum_{i=1}^{\#cell} A_{ij}$) and kept only the bins with Z-scored ${log}_{2}colsum$ between -2 and 2. The filtered matrix was normalized by dividing the row sum of the matrix to generate a term frequency matrix $TF$, and further converted to $X$ used for singular value decomposition $X=USV^{T}$, where $X_{ij}={log(TF}_{ij}\times100000+1)\times log(1+\frac{\#cell}{{colsum}_{j}})$.

We also generated a cell-by-loop matrix $B$ where each element indicated the imputed contact at each loop pixel in each single cell. This is a dense matrix of 5.7k x 3.2M, which limits the ability of this method to scale up to all cells in our dataset. We performed eigenvalue decomposition $BB^{T}=USU^{T}$ and rank the eigenvectors by the eigenvalues from large to small to derive the cell embedding $U$.

**Domain boundary-based.** We generated a binary cell-by-25kb bin matrix where each element indicated whether the bin was identified as a domain boundary in the cell. The same LSI framework was used to obtain the cell embedding.

**Clustering benchmark.** We applied L2 normalization within each cell on the top dimensions for all embeddings. For t-SNE visualization, we used top 25 dimensions, except in Higashi and fastHigashi we used top 128 dimensions. K-Means was used to perform clustering, and the top 50 dimensions were used, except for Higashi and fastHigashi, 128 dimensions were used. k was enumerated from 3 to 12 and the result with the highest adjusted rand index (ARI) compared to the cluster labels was shown in fig. S7. To benchmark the ability to separate excitatory cell types, we used L2/3-IT, L4-IT, L5-IT, L6-IT, L6-IT-Car3, L5/6-NP, L6b, L6-CT, and L5-ET. For inhibitory cell types, we used Lamp5-Lhx6, Lamp5, Sncg, Vip, Pvalb-ChC, Pvalb, and Sst.

The failure to resolve cell types could be due to biological reasons: 1) the differences of compartments across cell types are small, or 2) the heterogeneity of compartments across cells within the same cell type are huge, or technical reasons: 3) the power of algorithms to identify compartments on single-cell Hi-C data is limited. Based on the other analyses, we can identify the differential compartments between neuronal cell types as well as excitatory or inhibitory subtypes that strongly correlate with gene expression. This suggests that compartment differences exist at the pseudobulk level between finer-scale cell types that cannot be distinguished in compartment-based single-cell embedding. Thus, the single-cell heterogeneity or the computational challenges could be the major determinants. The analysis of chromosome imaging data could help further distinguish the two factors given that they are usually considered the gold standard for chromatin structures and do not need imputation algorithms for compartment calling. A previous study concluded small differences between compartments across single cells [(*78*)](https://paperpile.com/c/cCKYZi/NLU5n). Even though how large these differences are relative to the across cell type differences remain elusive and would need the chromosome/genome level chromosome imaging data from complex tissues to resolve. In summary, this result is the combined effect of biological feature specificity and computational limitations for quantifying the features accurately within single cells, and the conclusion is drawn from the best methods to date we can apply and might be challenged by technology and algorithm improvement.

Comparison between differential 3D genome structure and other modalities

**Compartment.** The raw compartment scores were quantile normalized across cell types. For each 100kb bin, we used this normalized score to compute its PCC with the ATAC, mCG, and mCH signals at the same 100kb bin across cell types. We also computed PCC between the normalized compartment scores with the expression level of genes whose promoters (TSS±2kb) or gene bodies (TSS-2kb to TES+2kb) overlap with the 100kb bin.

**Domain.** The boundary probability was defined at the start position of each 25kb bin. We used the ATAC, mCG, and mCH signals at the upstream and downstream 10kb bin of the boundary and took the average signal of the two bins to compute PCC with the boundary probability. We also computed PCC between the boundary probabilities with the expression level of genes whose promoters or gene bodies overlap with the two 10kb bins.

**Loop.** The interaction strength was defined for each loop pixel between two 10kb bins. We used the ATAC, mCG, and mCH signals at the two anchor bins of the loop and took the average signal of the two bins to compute PCC with the imputed loop strength (Q). We also computed PCC between the loop strength with the expression level of genes whose promoters or gene bodies overlap with the two 10kb bins, or whose gene bodies are between the two 10kb bins.

Note that the differences between compartment and domain or loop in correlation analyses could be due to the different resolution, given the usage of 100kb resolution for ATAC and methylation could dilute the signals of regulatory elements. Domain and loop are more comparable given that the quantification of ATAC and methylation signals are at 10kb resolution for the analyses.

Differentially expressed genes (DEGs) and comparison with 3D genome structures

Due to the differences between major type annotation, we assigned each RNA cell a major type label according to the mC cell based on the integration of neuronal cells between scRNA-seq data and snmC-seq data. The non-neuronal cells were labeled according to their original annotation given the clear correspondence between the two annotations. We randomly selected 1000 RNA cells from each major type, where the probability of a neuronal cell being chosen is proportional to the confidence of label transfer from mC cells to that RNA cell. This procedure provides 29k RNA cells in total from the 29 major types used in our 3C analysis. For each cluster pair, the p-values were derived with the Wilcoxon rank-sum test, and the fold-change is computed as the ratio between the average expression level across cells in the two clusters. The genes with an absolute value of log2 fold-change greater than 1 and False Discovery Rate (FDR, Benjamini-Hochberg Procedure) values smaller than 0.01 were considered as differentially expressed. The top 100 DEGs with the smallest FDR (BH procedure) were used as top DEGs between the cluster pair and the top results from all possible pairs were concatenated and duplicates were removed to generate a final list of top DEGs. This analysis identified 1099 top DEGs between neuronal major types and 1358 DEGs between all major types on autosomes.

We then calculated the Pearson Correlation Coefficient (PCC) between 3D genome structures and gene expression across neuronal major types. To avoid the bias led by the cutoff selection for differential analysis, we grouped the bins and genes based on the differential statistics and investigated the correlation for the bins and genes assigned to each group (figs. S11, E and F, and S12, E and F). For the expression level of each DEG, we also computed its correlation with the quantile normalized compartment scores of each 100kb bin, the boundary probability of each position with 25kb sliding interval, or the strength of loops within TSS-5Mb to TES+5Mb region of the gene (Fig. 2, K to N). We shuffled the 3D genome features and genes within each major type to calculate null PCC and estimate FDR. For each PCC value (denoted as x) between gene i and 3D genome feature j, a left-side FDR was computed as the ratio between the proportion of shuffled PCC smaller than x and the proportion of observed PCC smaller than x, and a right-side FDR was computed as the ratio between proportion of shuffled PCC greater than x and proportion of observed PCC greater than x. The PCC threshold corresponding to left-side and right-side FDR<0.01 was computed (denoted as tl and tr), and the final PCC threshold for significance was determined as ±(max(abs(tl), abs(tr))).

Cis-regulatory elements (CREs) prediction

Based on the pairwise CH-DMGs determined between cell subtypes, we assign a gene as hypomethylated in one subtype if it is a hypomethylated DMG in at least 40 out of 187 pairs compared with other subtypes. A DMR is assigned to a subtype if it is either CG-hypomethylated in the subtype (see section “*Determine differentially methylated regions*” above) or its CG-methylation level is below 0.3. A DMR is considered as a candidate cis-regulatory element if it is connected by a differential loop to a gene that is also a DMG in the same subtype. We do not require the differential loop connecting the DMR-DMG pair to be a loop detected in the subtype of the pair. The reason for this loose criterion is threefold: 1) the strength of the differential loop anti-correlates with the methylation levels (Fig 2K). If a DMR-DMG pair is connected with a differential loop in one subtype, the loop likely exists in another subtype with the DMR-DMG pair of similar methylation statuses; 2) Cis-regulatory elements are usually pleiotropic [(*79*)](https://paperpile.com/c/cCKYZi/uAmCc), The loops detected in subtypes covered by the m3c dataset could be reused in another uncovered if the methylation status of the DMR-DMG pairs is similar. 3) Loops could be missed in detection due to either the limitation of the computation methods or the insufficient coverage in certain subtypes. The DNA looping information transferring among subtypes could cope with such a situation to some extent.

Association between brain disorder risk variants and DMRs across cell types

We obtained GWAS summary statistics for quantitative traits related to neurological disease and control traits of intelligence [(*80*)](https://paperpile.com/c/cCKYZi/eSujj), educational attainment [(*81*)](https://paperpile.com/c/cCKYZi/iDSby), alcohol usage[(*82*)](https://paperpile.com/c/cCKYZi/cJmDu), Alzheimer's Disease [(*83*)](https://paperpile.com/c/cCKYZi/a4aSi), bipolar disorder [(*84*)](https://paperpile.com/c/cCKYZi/yWiI0), attention deficit hyperactivity disorder [(*85*)](https://paperpile.com/c/cCKYZi/GIfIW), neuroticism [(*86*)](https://paperpile.com/c/cCKYZi/4f4uL), schizophrenia [(*87*)](https://paperpile.com/c/cCKYZi/h8xSc), amyotrophic lateral sclerosis [(*88*)](https://paperpile.com/c/cCKYZi/kfrEn), tobacco use disorder [(*89*)](https://paperpile.com/c/cCKYZi/CiUtF), insomnia [(*90*)](https://paperpile.com/c/cCKYZi/EKAOk), sleep duration, coronary artery disease [(*91*)](https://paperpile.com/c/cCKYZi/6Yumh), height, tiredness [(*92*)](https://paperpile.com/c/cCKYZi/NWMm0), type 1 diabetes [(*93*)](https://paperpile.com/c/cCKYZi/Vj2T9), type 2 diabetes [(*94*)](https://paperpile.com/c/cCKYZi/Waqsi), allergy [(*95*)](https://paperpile.com/c/cCKYZi/OMo8u), birth length [(*96*)](https://paperpile.com/c/cCKYZi/ozElW), and birth weight [(*97*)](https://paperpile.com/c/cCKYZi/Vs11F).

We prepared summary statistics in the standard format for linkage disequilibrium score regression. Next, we converted major-type hypo-DMRs to human genome assembly GRCh37 (hg19) coordinates using the software LiftOver, and annotated with the 1000 Genomes Project Phase 3 SNPs [(*98*)](https://paperpile.com/c/cCKYZi/dsuF8). The superset of the hypo-DMRs was used as the background. Finally, we used cell-type-specific linkage disequilibrium score regression (<https://github.com/bulik/ldsc>; [(*40*)](https://paperpile.com/c/cCKYZi/vgdPQ)) to estimate the enrichment coefficient of each annotation for each trait.

Brain regional axes from DNA methylation profiles

Both CG- and CH- highly variable 100kb-bins of one cell type (the same features for clustering analysis) were used to compute a lower dimensional representation with the principle component analysis (PCA). First, a neighbor graph of the cells was constructed in the PCA space. Then, for each cell, a regional identity vector was computed by averaging the location information of this cell and its neighbors. A pairwise Manhattan distance matrix was then constructed from the regional identity vectors to capture relations among brain regions. The principle coordinate analysis (PCoA) was applied to this distance matrix to obtain a lower-dimensional embedding in the regional space as well as preserve relative distances among cells. Thus, the cells were transformed from the methylome space to the regional space.

In the regional space, we perform the trajectory analysis with the Elastic Principal Graph (EPG) algorithm implemented in STREAM [(*49*)](https://paperpile.com/c/cCKYZi/vSFqh). The parameters epg_alpha, epg_mu, and epg_lambda were manually adjusted to ensure the resulting trajectories well represented the distributions of the cells in the regional space. Each cell was assigned a regional index (or pseudotime) range in [0,1] according to its relative position to the trajectory. The cells were then grouped into 20 bins along the trajectories based on their regional index. The mean DNA methylation profiles can be computed for each bin.

**Consensus regional axis for cortex and basal ganglia.** The mean regional index was first computed for cells from each cortical region in each cell type. Then the average regional indices were calculated by averaging the mean regional indices across the corresponding cell types. Finally, the consensus regional axis was constructed by ranking the average indices.

**Regional DMGs.** We used a one-vs-rest strategy to calculate region-specific CH-DMGs (rDMGs) within major types from the cortex and basal ganglia. To avoid potential bias caused by an imbalance of cell numbers in different regions, we downsampled cells in each region to no more than 500. Using the Wilcoxon rank-sum test, protein-coding genes and lncRNAs were tested for significant methylation decrease (or hypomethylation). The p-values were adjusted with multitest correction using the Benjamini-Hochberg procedure. The genes with adjusted p-values≤1^-10^ and log2 fold-change ≤ -0.1 were considered rDMGs.

**Regional DMRs.** Cells from the same brain region were merged for each major type to construct the regional pseudo-bulk methylation profiles. Then the DMRfind function of the software MethylPy was used to determine the candidate rDMRs with the same options as in determining cell-type DMRs. If a candidate rDMR has CG-methylation variation ≥ 0.6 across regions tested, it is considered an rDMR.

**Regional enriched motifs of TFs.** For simplicity, we selected rDMRs with methylation levels changing monotonically (PCC≥0.5 or ≤-0.5) with the regional axes that we revealed in cortex and basal ganglia (Fig 4H&C), and performed the TF motif enrichment analysis against cell type-specific hypomethylated DMRs. TF motifs with adjusted p-val <1e-50 were considered as enriched.

Enrichment analysis on conserved DMRs between human and mouse

**Functional enrichment analysis of hcCnsvDMRs.** The Genomic Regions Enrichment of Annotations Tool (GREAT) [(*99*)](https://paperpile.com/c/cCKYZi/sni9g) was used to compute the Gene Ontology (GO) term enrichment of hcCnsvDMRS. “Basal+extension” option (5.0 kb upstream, 1.0 kb downstream, and up to 100 kb max extension) was selected for gene association, and “curated regulatory domains” are included in the analysis.

**Comparison between hcCnsvDMRs and histone modification marks in mouse forebrains.** Replicated peaks of histone modification marks of P0 mouse forebrain were downloaded from the Encode project [(*4*)](https://paperpile.com/c/cCKYZi/n3FgY). Particularly, H3K27ac (ENCFF044YBD), H3K27me3 (ENCFF461UUN), H3K4me1 (ENCFF467MYU), H3K4me3 (ENCFF066LGF), and H3K9me3 (ENCFF997XJK) were used. The software Genomic Association Tester (GAT, v1.3.6)[(*100*)](https://paperpile.com/c/cCKYZi/uaJFT) was used to compute the enrichment of hcCnsvDMRs in the histone modification marks. Accessibility of hcCnsvDMRs was determined by comparing them with snATAC peaks profiled from P56 mouse brains [(*2*)](https://paperpile.com/c/cCKYZi/O9Ftg).

scMCode construction

**Candidate CpG sites.** We constructed the pseudo-bulk mCG profile for each major type and then iteratively selected CpG sites to distinguish all major types. In each iteration, CpG sites were selected according to the criteria: 1) they are either almost entirely methylated (mCG%≥80%) or unmethylated (mCG%≤20%) among all the remaining major types; 2) both two methylation statuses are presented among the remaining major types; 3) The CpG sites should have coverage ≥10 in ≥80% of the remaining major types. These selected CpG sites were added to the CpG site pool for later scMCode construction. In each iteration, the methylation levels of the selected CpGs in the remaining major types were binarized if they are ≥80% or ≤20%. Pairwise distances were computed between binarized methylation status, and the cell types that had a distance <20 to any of the other major types were kept for the next iteration of CpG selection. In total, 221,140 CpG sites were selected as candidates for scMCode construction.

**CpG site selection for scMCode.** The methylation levels of candidate CpG sites across all the major types were trinary-discretized based on their DNAm fractions (discretized values are -1 for mCG%≤20%, 1 for mCG%≥80%, and 0 for 20%<mCG%<80%) in major type pseudo bulk level. The CpG sites were further grouped into 38,945 features based on these discretized DNAm status across major types. To prevent scMCode from bias caused by cell type population differences or individual variations of donors, we randomly select 300 cells from each major type from each donor as the dataset for scMCode construction. In each cell, the methylation state of each feature was either computed by averaging the methylation levels of all the CpG sites belonging to this feature (AverageCpG) or by directly using the methylation level of a randomly picked single CpG site belonging to this feature (RandomCpG). The cell-by-feature matrix was trinary-discretized based on their DNAm fractions (discretized values are -1 for mCG%<50%, 1 for mCG%>50%, and 0 for mCG%=50% or uncovered) in single cell level and then used to train a random forest (RF) model to predict major types. A 4-fold cross-validation scheme was used to prevent overfitting. Finally, the top 800 most important features were selected to construct the scMCode for major types. We observed no difference in predicting performance between AverageCpG and RandomCpG, indicating the robustness of scMCode.

**Boost the prediction accuracy with K-Nearest-Neighbor imputation.** We achieved ~88% single-cell predicting accuracy directly with the cell-by-feature matrix. Given the limited coverage of single-cell data, the cell-by-feature matrix could be further imputed to improve the prediction accuracy. Within the training dataset, we randomly selected half of the cells and merged them into pseudo-cells according to their major types. This process was repeated 20 times, and a pseudo-cell-by-feature matrix was constructed from these pseudo-cells in the same way. A K-Nearest-Neighbor (KNN) imputer was built upon this matrix. The testing dataset was first imputed with the KNN imputer and then fed to the RF model for prediction. The KNN imputation step improved the prediction accuracy to ~93%.

**Cross-donor tests.** We derived scMCodes for each donor, and trained the KNN imputer and RF classifier correspondingly. The single donor scMCodes and models were then applied to the data of other donors to assess the cross-individual robustness. When the training and testing donors are the same (diagonal in Fig 6E), a 4-fold cross-validation scheme is used to prevent overfitting and to assess the accuracy.

**Supplementary Text**

Figure related notes

**Figure 2.** (I) Sample sizes are 1188, 2047, 173615, 1024, 1716, 148250 from left to right for each subplot. (J) Sample sizes are 619, 1259, 710, 1716, 168938, 56027, 247125, 55, 343, 84, 417, 6684, 9303, 81904 from left to right.

**Figure 4.** (F) The relative domain strengths (blue numbers) are ratios between the summations of all bins within each domain; the relative promoter strengths (purple numbers) are ratios between the summations of all bins related to the bin of NR2F1 promoter within each domain.


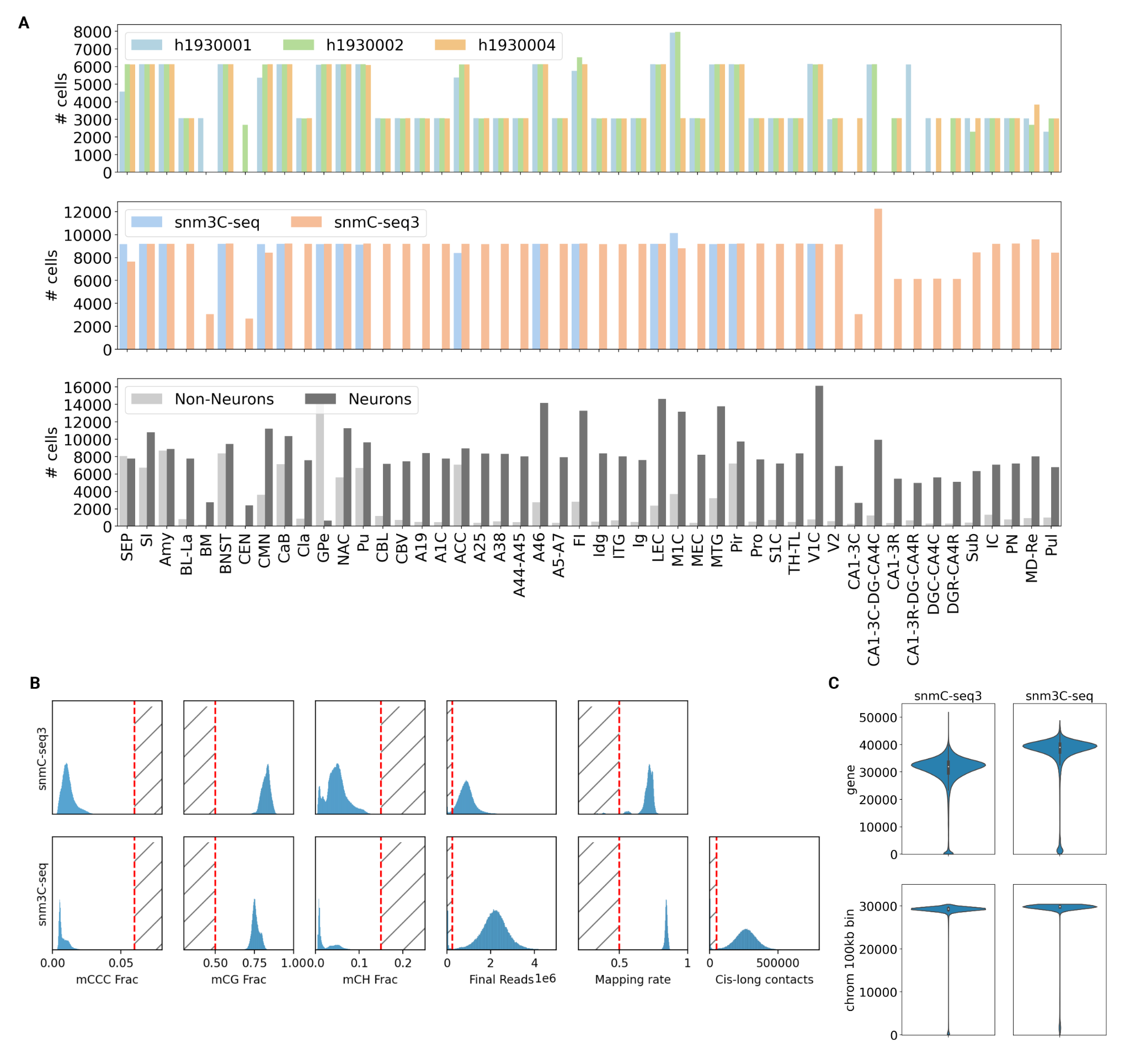


**Figure S1. Sample information and QC metrics.** (A) Cell number distributions of donors (top), epigenetic profiling assays (mid), and neuronal/non-neuronal cell types (bottom) from different brain regions. (B) QC metrics are used in filtering cells in snmC-seq and snm3C-seq. (C) Coverage per cell distributions of genomic features of genes and 100kb-bins in mC and m3C datasets.


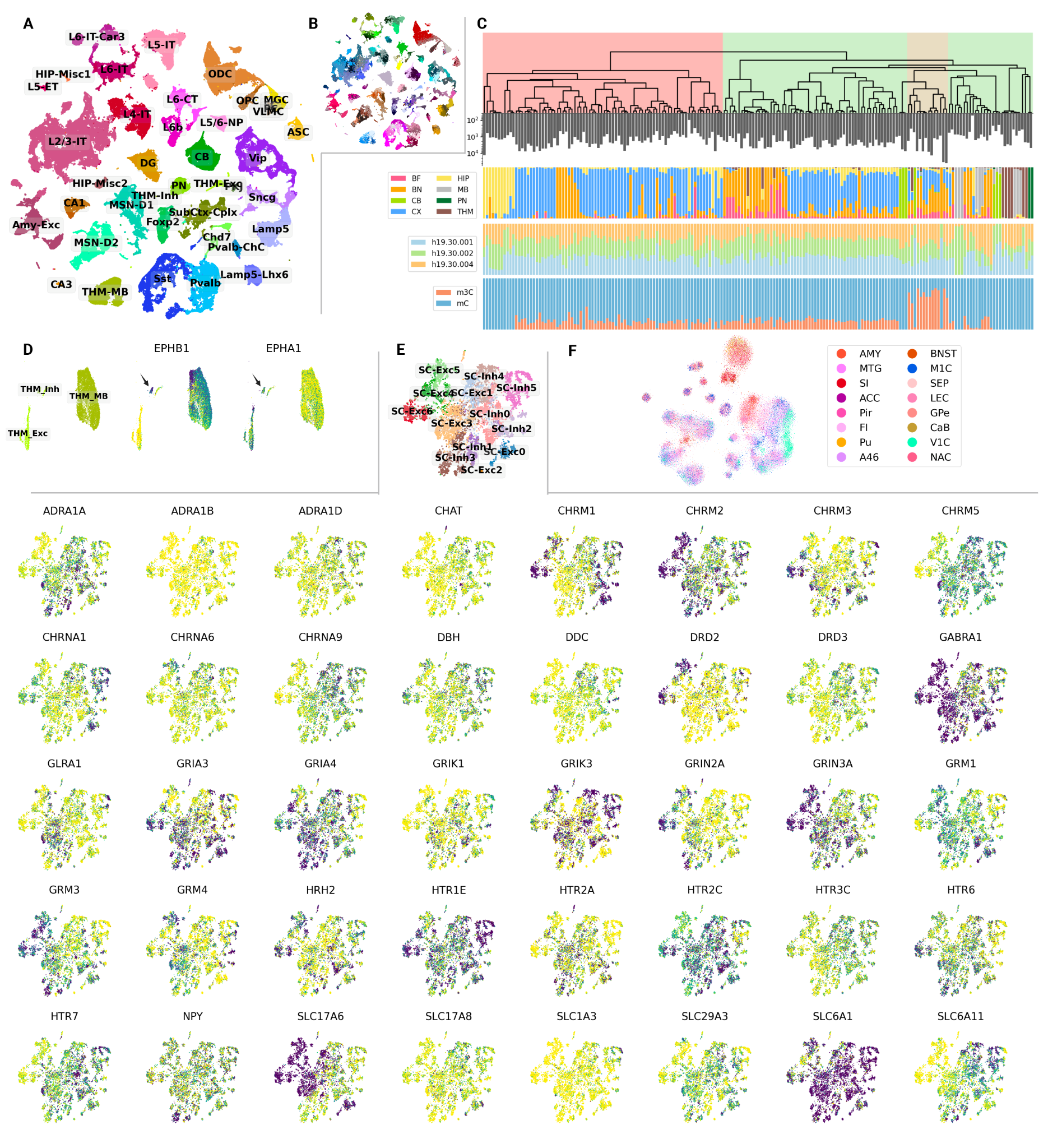


**Figure S2. Clustering and annotation of brain cell types.** (A) 2D t-SNE visualization of mC cells colored by 40 major types. (B) 2D t-SNE visualization of mC cells colored by 188 subtypes. (C) The robust dendrogram of the subtypes and the corresponding meta info of brain structures, donor origins, and epigenetic profiling assays. (D) The thalamus major type THM-Inh is hypomethylated in genes EPHB1 and EPHA1 which are specifically expressed by habenular nuclei of thalamus. (E) Subtypes of SubCtx-Cplx major type show highly variable DNA methylation in the genes of neurotransmitter receptors, transporters, and neuropeptides. (F) Regional diversity of single-cell embedding computed from chromatin conformation. The embedding is the same as in Fig 1G, while colored by the dissection regions.


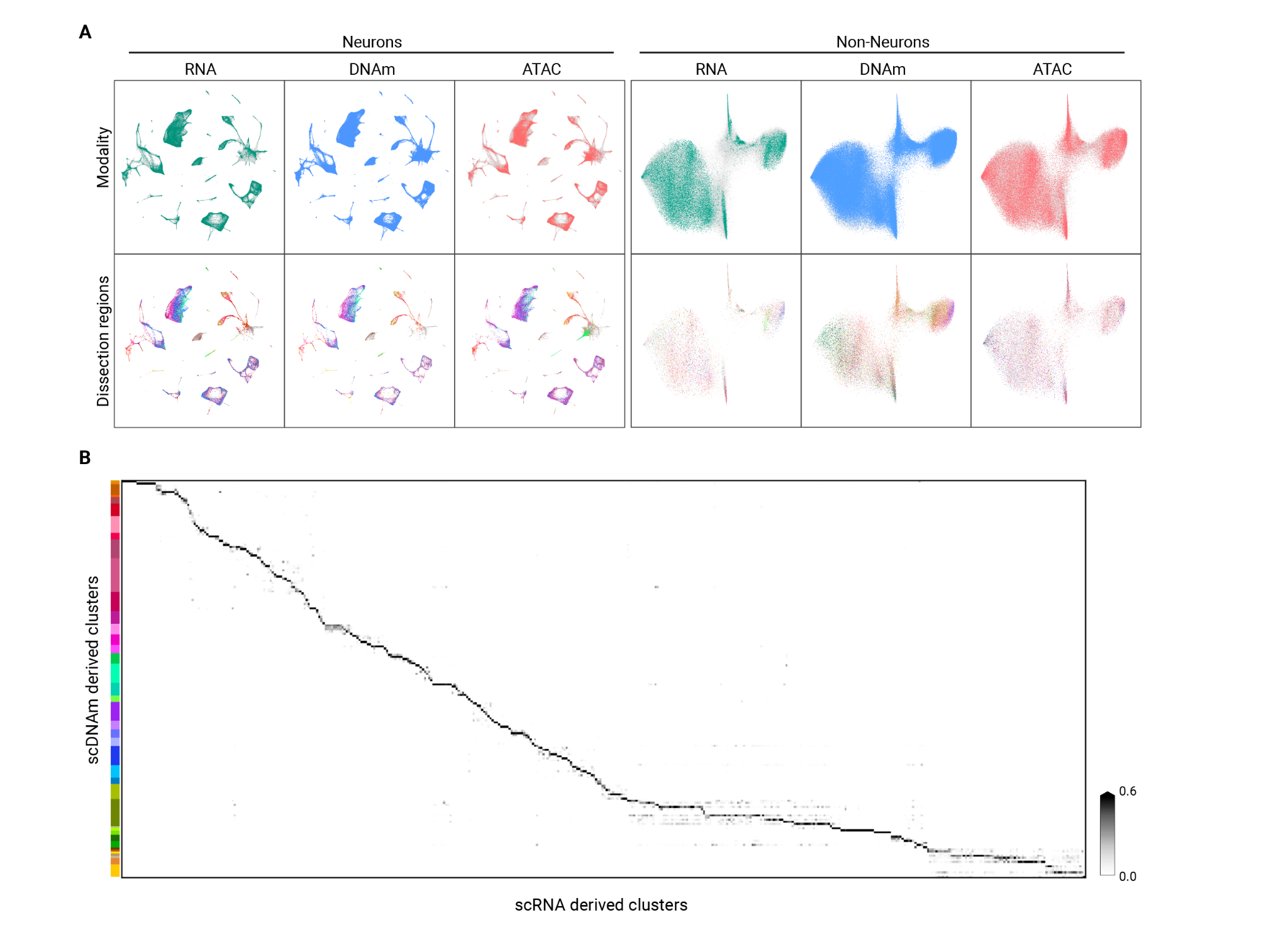


**Figure S3. Integration between modalities.** (A) 2D t-SNE visualization of integration results between snmC, scRNA, and snATAC datasets. The integration shows cell types and regional diversity are consistent among the three modalities. (B) Heatmap shows the cross-tabulation between scRNA cluster labels and transferred mC annotation. The count number was normalized by rows.


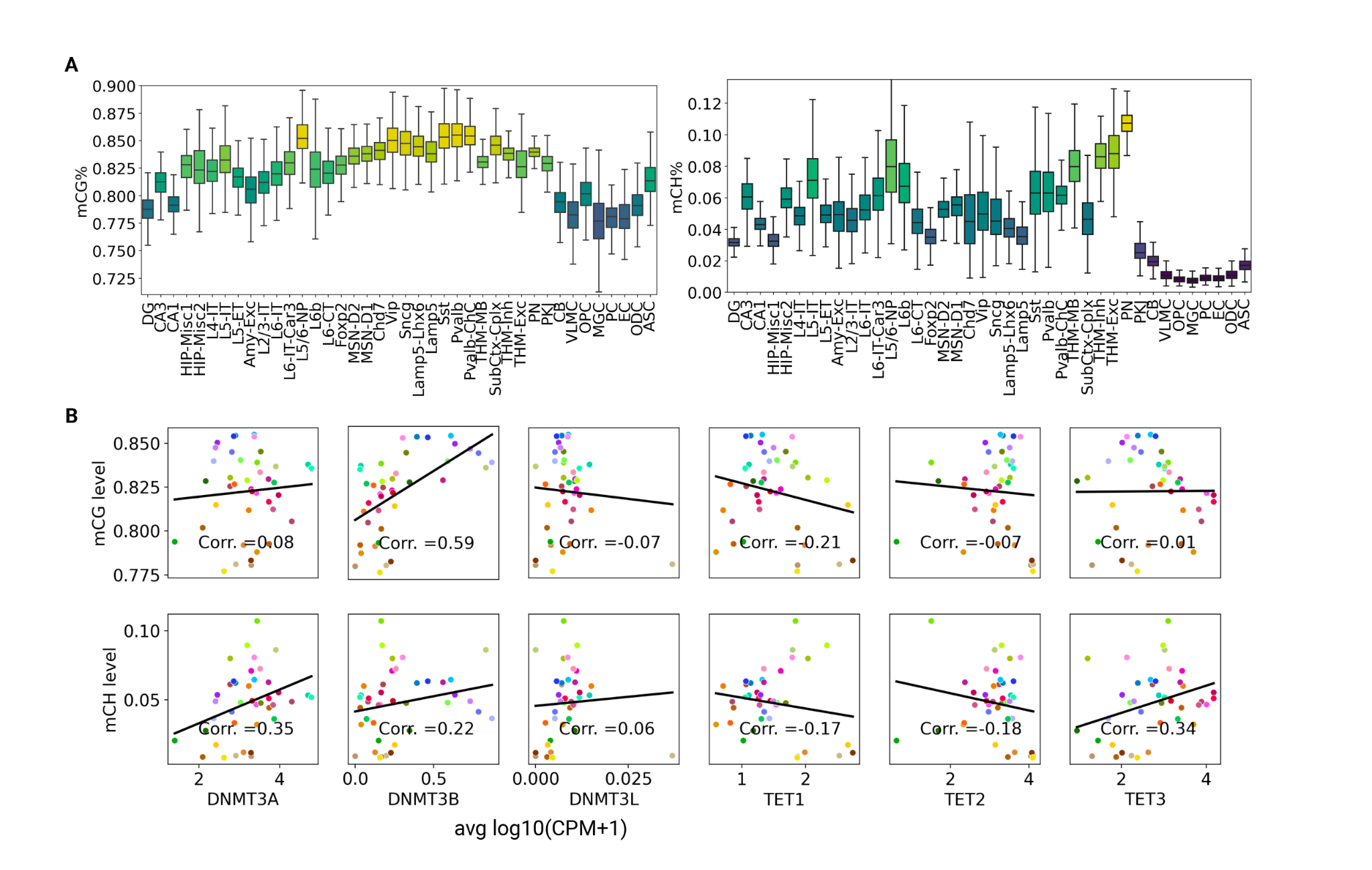


**Figure S4. Global methylation levels across major types.** (A) Global CG- and CH-methylation levels of major types. (B) Pearson correlation between global CG- and CH-methylation levels and gene expression of DNA methylation readers/modifiers across major types.


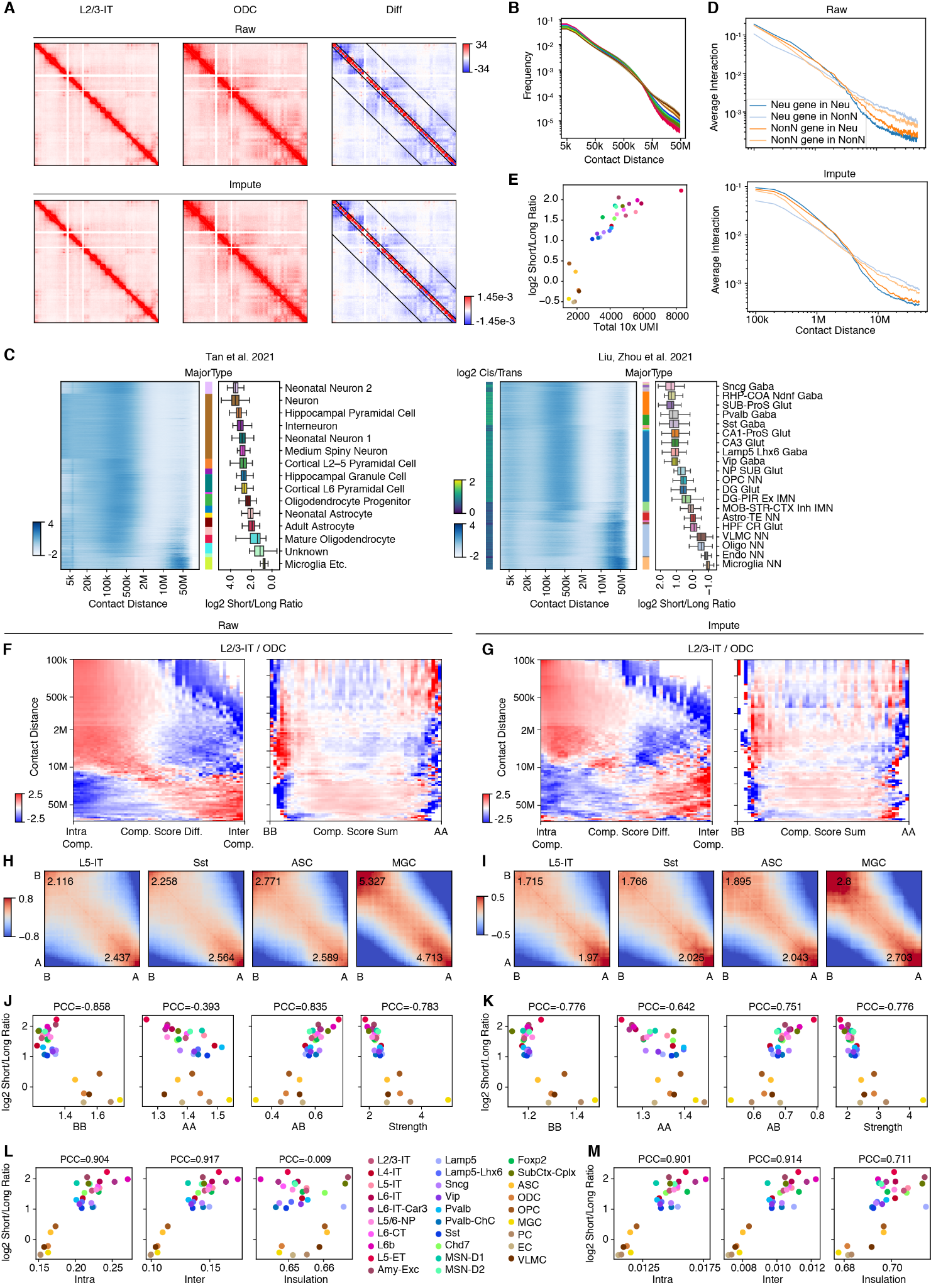


**Figure S5. Diversity of contact distances across major types.** (A) Raw (top) and imputed (bottom) contact map of L2/3-IT (neuron, left), ODC (non-neuron, middle), and the subtraction of ODC from L2/3-IT (right) at 100 kb resolution. The color bars are shared within each row. (B) Frequency of contacts against genomic distance in all major types. Contacts are grouped in an arithmetic scale of distance while the x-axis shows a log scale of distance, so sample points are denser on the right than on the left. (C) Contact distance of mouse brain cells from Tan et al. 2021 (left) and Liu, Zhou et al. 2021 (right). Heatmap shows frequency of contacts against genomic distance in each single cell, Z-score normalized within each cell (row). The x-axis is binned at log2 scale. The bars show the major type and log2 cis/trans ratio (when the information is available) of each cell. Boxplot shows the log2 short/long ratio of major types. Color palettes are shared between the major type bars and the boxes. Centerline denotes the median; box limits denote the first and third quartiles; and whiskers denote 1.5 × the interquartile range. (D) Frequency of contacts in L2/3-IT (dark) or ODC (light) raw (top) or imputed (bottom) contact map against genomic distance for the top 100 DEGs with a higher expression level in L2/3-IT than ODC (Blue) or reversely (Orange). The contacts are grouped in an arithmetic scale of distance while the x-axis shows a log scale of distance, so sample points are denser on the right than on the left. (E) Median log2 ratio between short and long-range contact (defined in Methods) frequencies against average UMI detected per cell. The color palettes are shared across the whole manuscript. (F and G) The ratio between L2/3-IT and ODC of frequency of contacts grouped by genomic distance and the difference (left) or summation (right) of compartment scores at the two anchors of a contact (Methods), Z-score normalized within each distance (row). Raw compartment scores were used in (F) and imputed compartment scores in (G). (H and I) Saddle plots of the four cell types are shown in Fig. 2, C to E (Methods). The axes are ranked by the compartment score of the cell types. Values are average distance-normalized raw (H) or imputed (I) contacts. The number at the corner represents the ratio between BB and BA interaction strength (top left) or the ratio between AA and AB interaction strength (bottom right). (J to M) The relationship across major types between log2 short/long ratio and interaction strength between BB compartment, AA compartment, AB compartment, and compartment strength (AA+BB)/(AB+BA) (J and K), or intra-domain interaction strength, inter-domain interaction strength, and insulation score (inter/intra) (L and M) on raw (J and L) or imputed (K and M) contact maps.


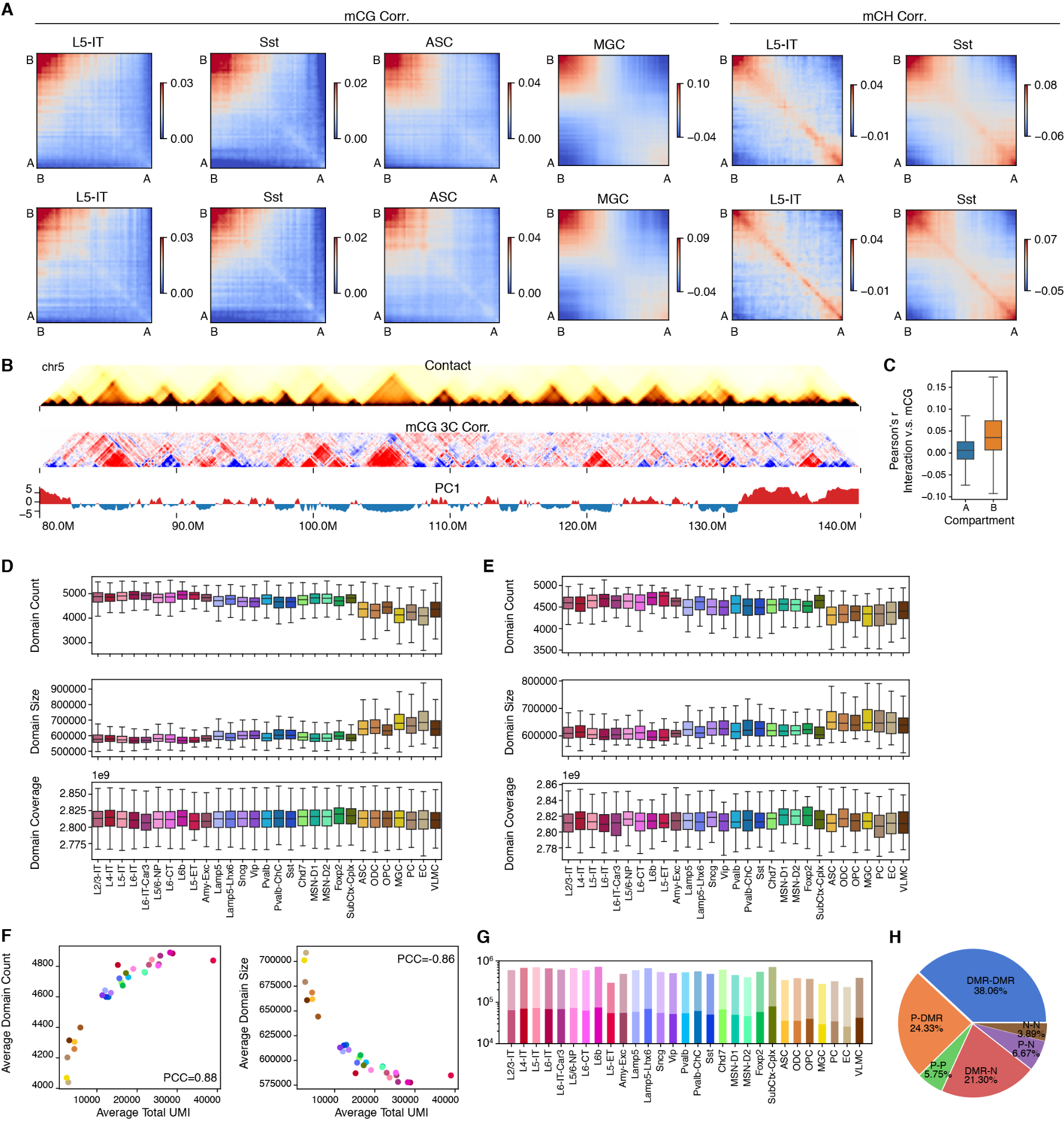


**Figure S6. Compartment, domain, and loop in brain cells.** (A) Saddle plots of the four cell types are shown in Fig. 2, C to E (Methods). The axes are ranked by the raw (top) or imputed (bottom) compartment score of the cell types. Values are the average correlation of mCG (left) or mCH (right) level between pairs of 100kb bins. For mCH correlation, only neuronal types are shown. (B) Imputed contact map (top), the correlation between 3C and mCG across single cells (middle), and compartment score computed from raw contact matrices (bottom) at 100kb resolution. (C) Correlation between 3C and mCG in the triangle region of A compartment segments (n=346,260) and B compartment segments (n=172,045). (D and E) Number of domains (top), size of domains in bp (middle), and total basepairs within domains (bottom) of major types quantified within each single cell using all the cells (D) or only the cells with a matched number of contacts within 10 Mb across cell types (E). (F) Relationship between average total unique molecular identifiers (UMIs, x-axis) and average domain count (top, y-axis) or average domain sizes (bottom, y-axis) over single cells across major types. (G) Number of loop pixels (transparent color) and loop summit (solid color) in major types. (H) Proportion of different categories of loops. P denotes loop anchors overlapping with promoters (TSS±2k), DMR denotes loop anchors overlapping with DMRs but not promoters, and N denotes loop anchors overlapping with neither promoters nor DMRs. For all the boxplots, the center line denotes the median; box limits denote first and third quartiles; and whiskers denote 1.5 × the interquartile range.


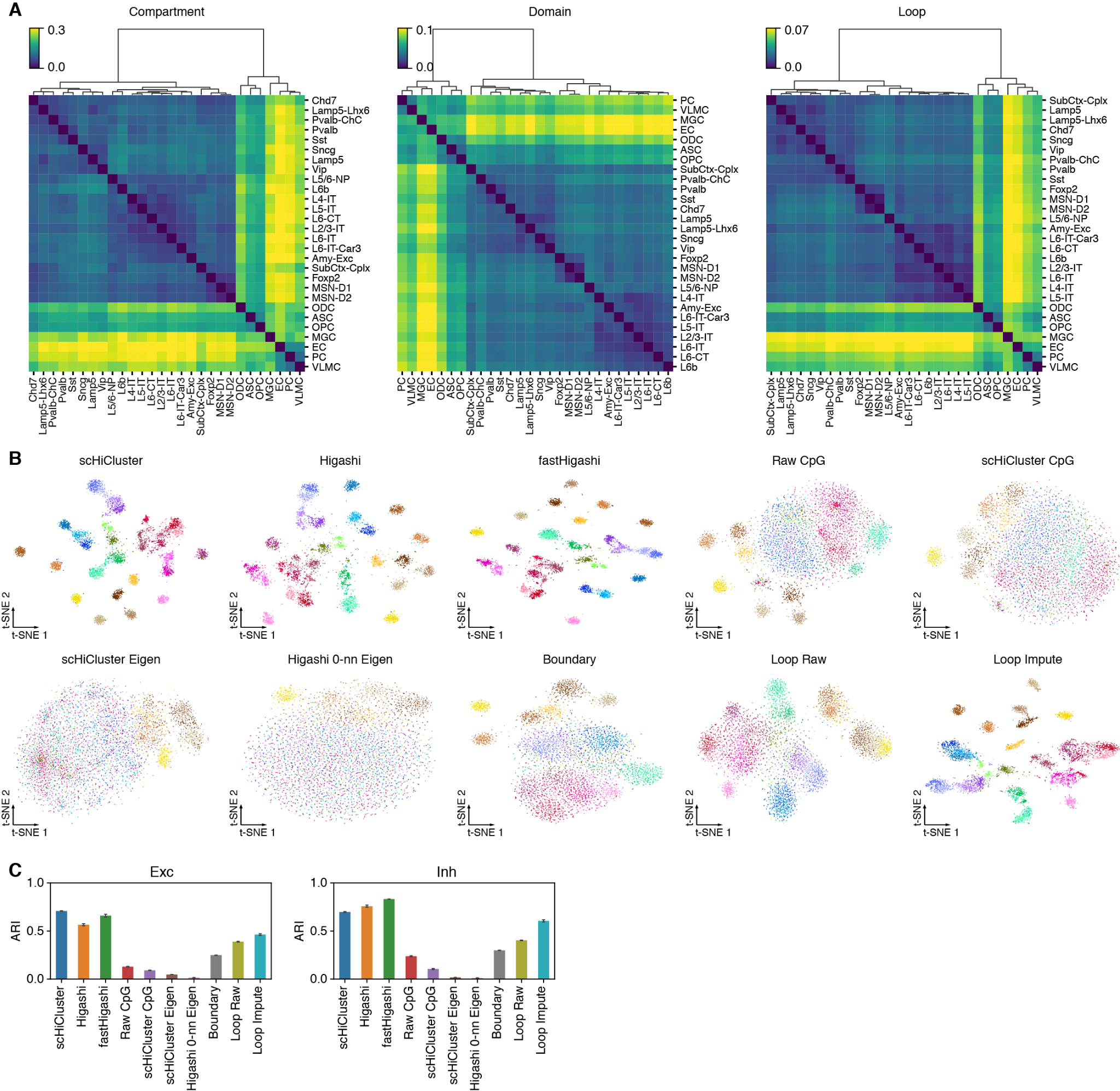


**Figure S7. Specificity of compartment, domain, and loop.** (A) Cosine distances between major types are measured by raw compartment score (left), boundary probabilities across all 25kb bins (middle), or imputed contact strengths across all loop pixels identified in at least one cell type (right). (B) t-SNE of human brain cells (n=5,707) using different methods (Methods). (C) Adjusted Rand Index (ARI) between clusters using the cell embedding generated from the different methods as features and cortical excitatory (left) or cortical inhibitory (right) major type labels. The error bars represent the standard error of the mean of K-Means clustering with ten different random seeds for initialization.


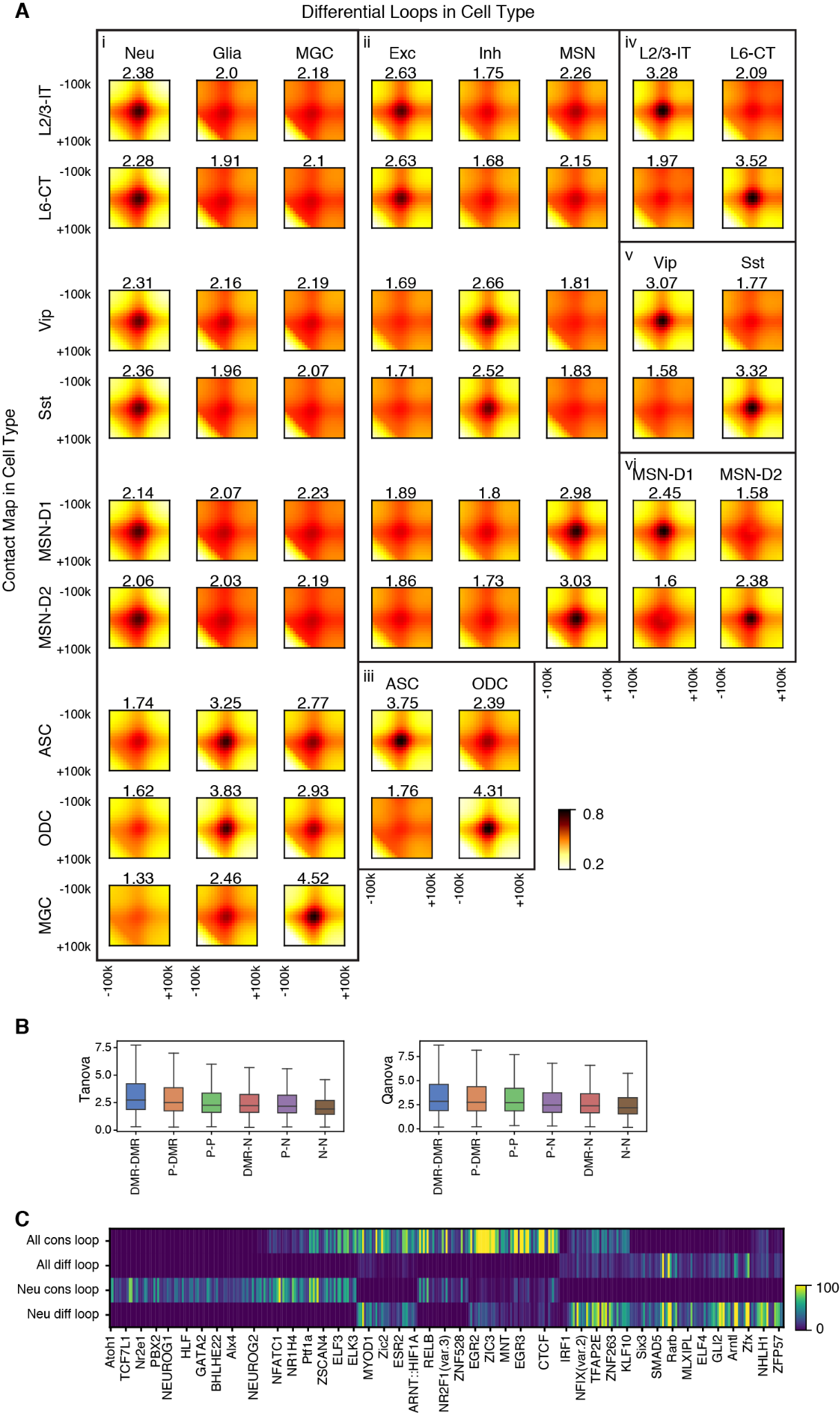


**Figure S8. Differential loop across brain major types.** (A) Aggregate peak analysis (APA, methods) of differential loops between neuron, glia, and non-neuronal cells (i), between excitatory, inhibitory, and MSN neurons (ii), between ASC, ODC and OPC (iii), between excitatory major types (iv), inhibitory major types (v), or MSN-D1 and MSN-D2 (vi). (B) ANOVA statistics of different categories of loop pixels are computed with T (left) or Q (right). (C) Log10 q-value (Fisher exact test, Benjamini-Hochberg procedure) of motif enrichment in differential loops and constant loops compared to their union of them.


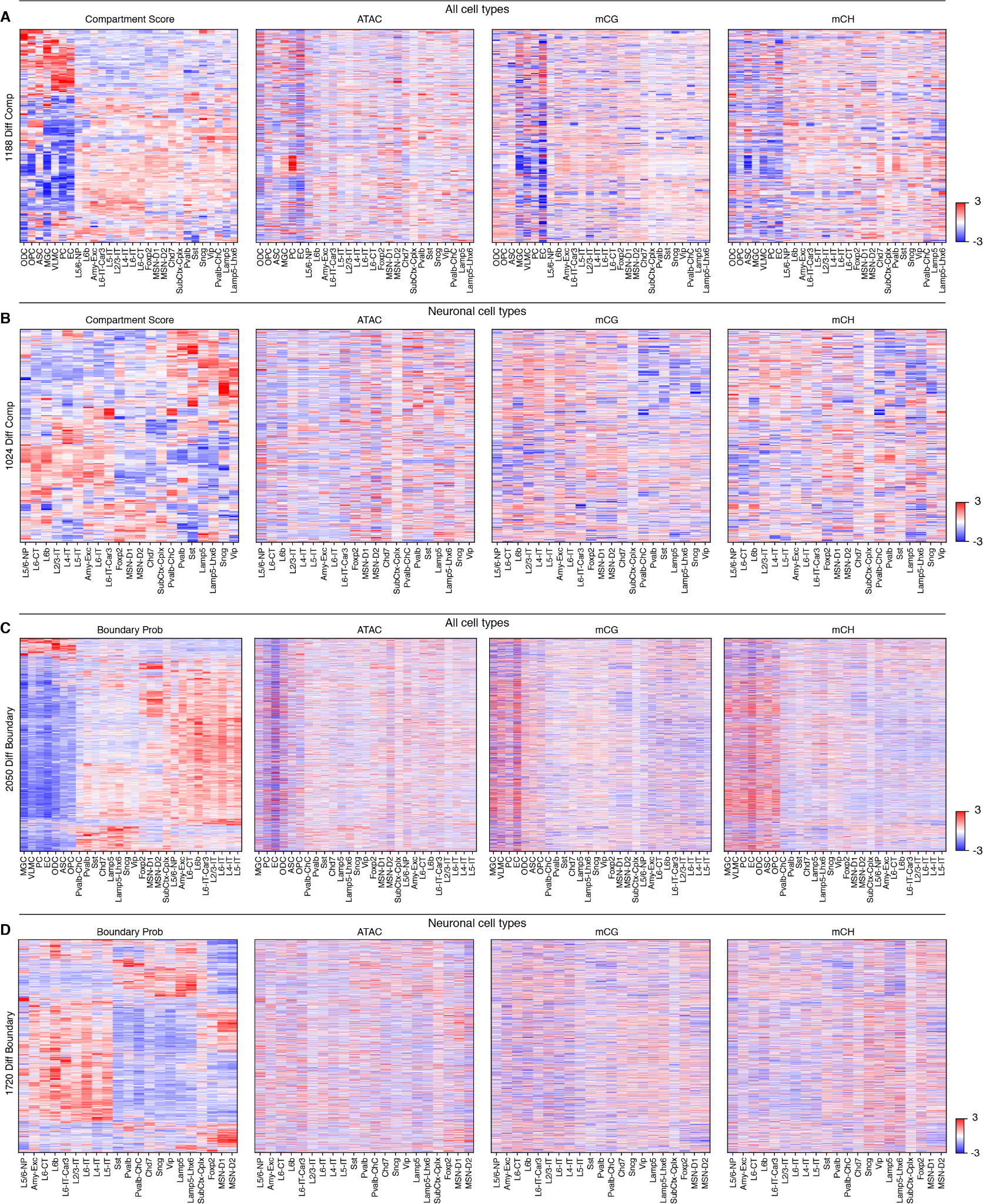


**Figure S9. Correlation between compartment, domain and epigenome.** (A and B) Compartment score, ATAC signals, mCG and mCH level of differential compartments at 100 kb resolution between all major types (A) or all neuronal major types (B). (C and D) Domain boundary probabilities of differential boundaries at 25 kb resolution across all major types (C) or all neuronal major types (D), and average ATAC signals, mCG and mCH level of the two 10kb bins on both sides of the boundaries. Values are Z-score normalized within each row. All four heatmaps in the same row share the row and column orders.


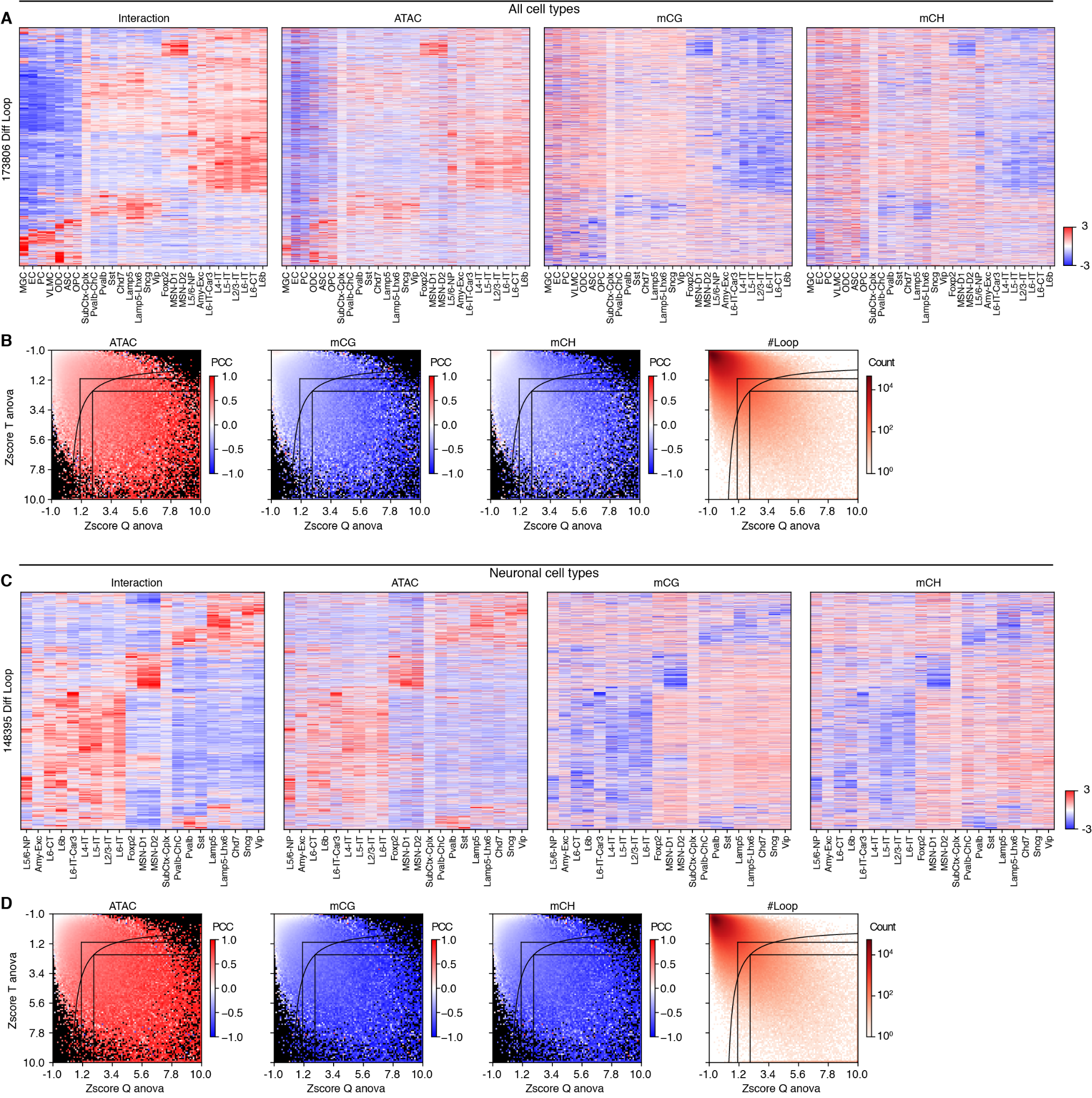


**Figure S10. Correlation between loop and epigenome.** (A and C) Interaction strength of differential loops at 10 kb resolution across all major types (A) or neuronal major types (C), and average ATAC signals, mCG and mCH level of the two anchors of the differential loops. Values are Z-score normalized within each row. All four heatmaps in the same row share the row and column orders. (B and D) PCC between interaction strength and average ATAC signal (left), mCG (middle left), or mCH (middle right) level at two anchors or the number of loop pixels (right) with different T and Q ANOVA statistics.


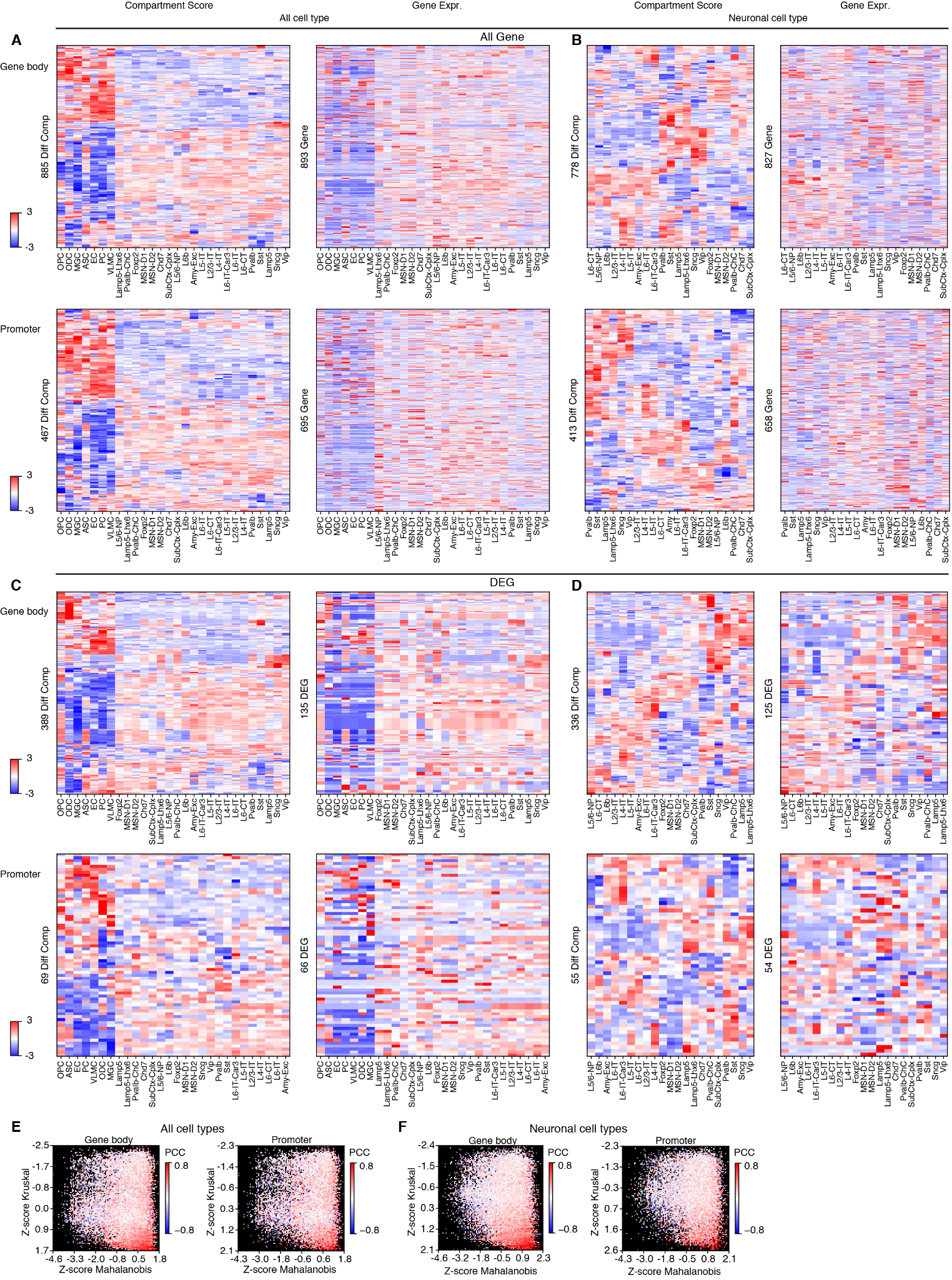


**Figure S11. Correlation between compartment and transcription.** (A to D) Raw compartment scores (left) of differential compartments at 100kb resolution across all major types (A and C) or neuronal major types (B and D) and expression level (right) of all genes (A and B) or DEGs (C and D) whose gene body (top) or promoter (bottom) overlap with the 100kb bin. Values are Z-score normalized within each row. Left and right heatmaps share the row and column orders. When a bin overlaps multiple genes, the bin is repeated in the left heatmap, and vice versa for a gene overlapping multiple bins. (E and F) Average PCC between compartment score and gene expression across all major types (E) or neuronal major types (F) for the bins and genes showing different diversity between cell types. PCC was computed for each pair of 100kb bin and gene when the bin overlapped the gene promoter (left) or gene body (right). RNA diversity was quantified by the Z-score of Kruskal statistics for expression level between cell types. Compartment diversity was quantified by the Z-score of Mahalanobis distance between cell types.


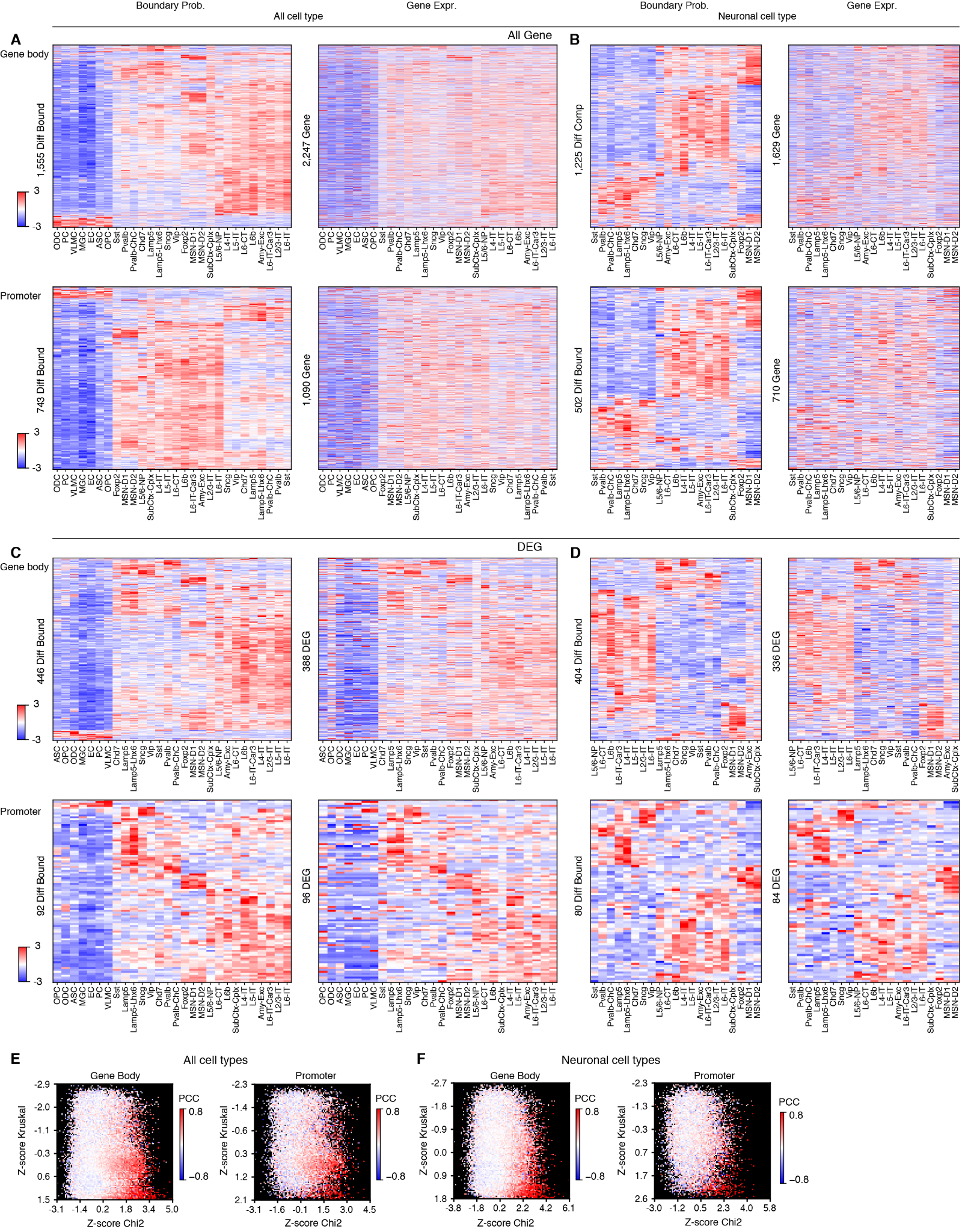


**Figure S12. Correlation between domain and transcription.** (A to D) Domain boundary probability (left) of differential boundaries at 25kb resolution across all major types (A and C) or neuronal major types (B and D) and expression level (right) of all genes (A and B) or DEGs (C and D) whose gene body (top) or promoter (bottom) overlap with the 20kb flanking region of the differential boundaries (left end of the 25kb bin). Values are Z-score normalized within each row. Left and right heatmaps share the row and column orders. When a bin overlaps multiple genes, the bin is repeated in the left heatmap, and vice versa for a gene overlapping multiple bins. (E and F) Average PCC between boundary probability and gene expression across all major types (E) or neuronal major types (F) for the boundaries and genes showing different diversity between cell types. PCC was computed for each pair of boundary and gene when the flanking 20kb region of boundary overlapped the gene promoter (left) or gene body (right). RNA diversity was quantified by the Z-score of Kruskal statistics for expression level between major types. Boundary diversity was quantified by the Z-score of Chi-Square statistics between major types.


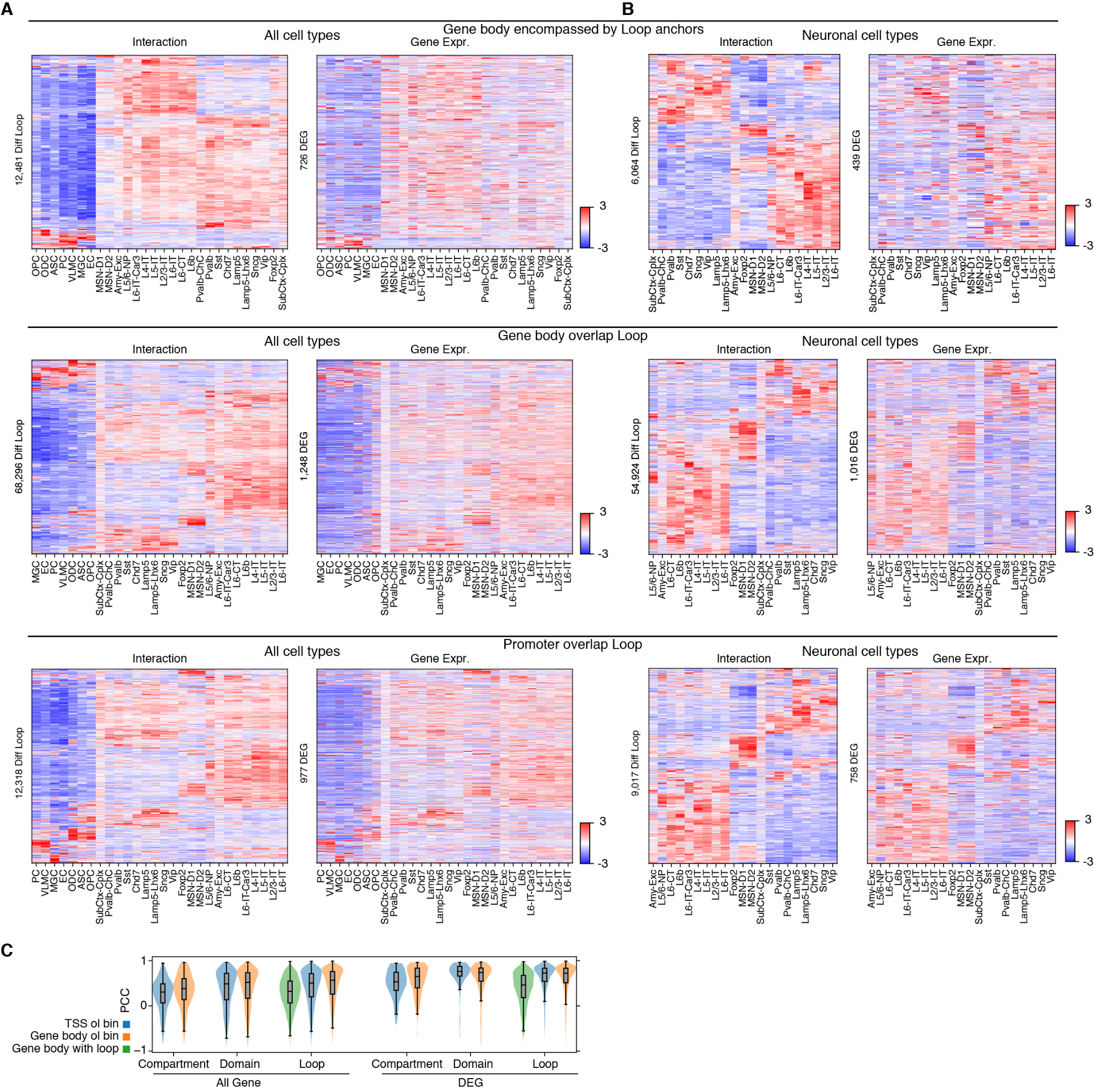


**Figure S13. Correlation between loop and transcription.** (A and B) Interaction strength (left) of differential loops across all major types (A) or neuronal major types (B) and expression level (right) of DEGs whose gene body is encompassed by the two anchors of the differential loops (top), gene body (middle) or promoter (bottom) overlap either anchor of the differential loops. Values are Z-score normalized within each row. Left and right heatmaps share the row and column orders. When a loop overlaps multiple genes, the loop is repeated in the left heatmap, and vice versa for a gene overlapping multiple loops. (C) PCC between compartment score, boundary probability, or loop interaction strength and gene expression across all major types for different categories of overlap described above (x-axis) for all genes (left) or top DEGs only (right). Sample sizes are 711, 1386, 1090, 2331, 295078, 86626, 312990, 69, 396, 96, 460, 14306, 12718, 99281 from left to right.


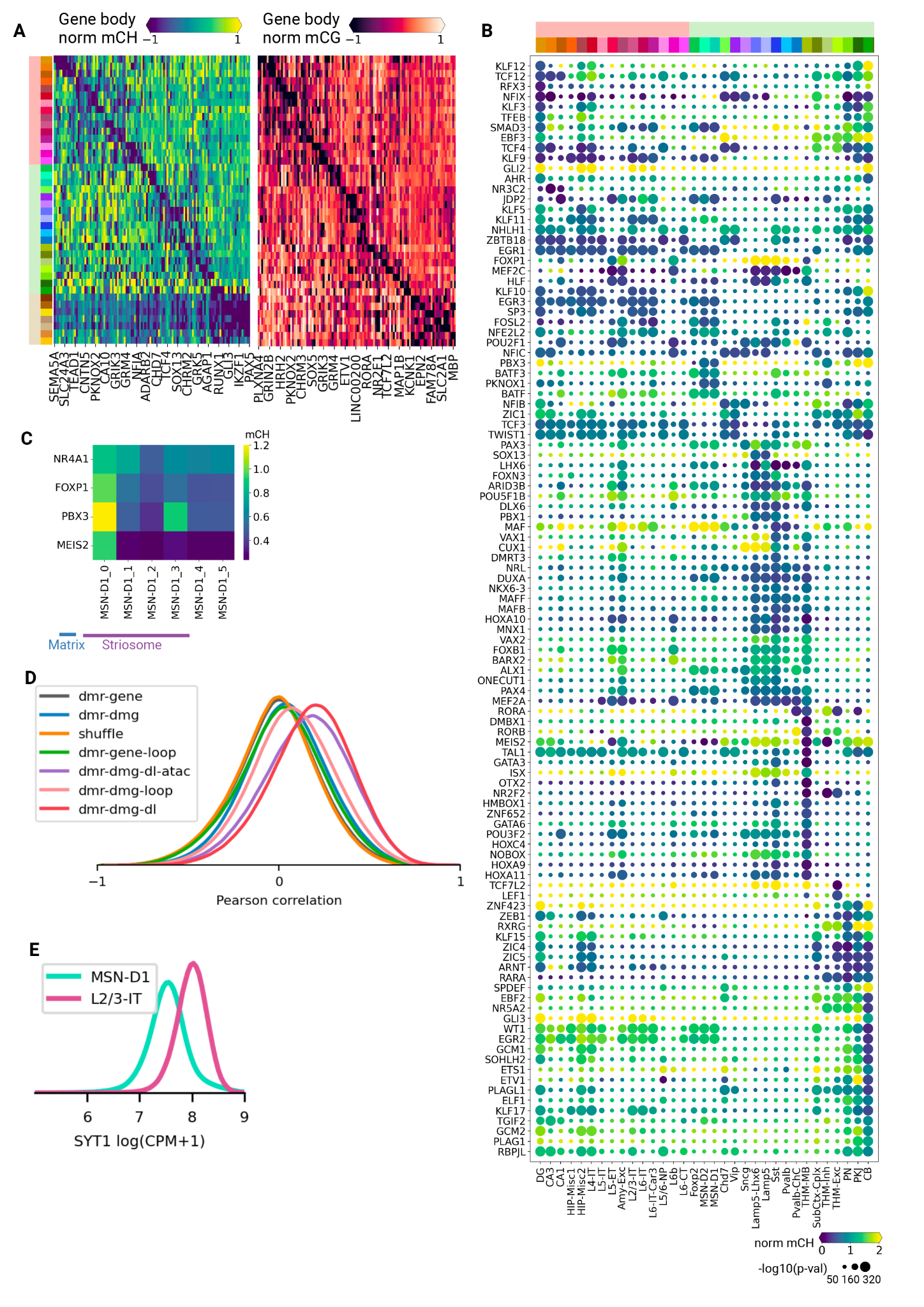


**Figure S14. Gene regulation in brain cells.** (A) Different major types have specific marker genes in both CG- and CH-methylation. All marker genes shown in the heatmaps are TFs, neurotransmitter receptors, transporters or neuropeptides. (B) The scatter plot of CH-methylation and enrichment of TFs that were assigned to the major types. (C) Heatmap shows average CH-methylation levels of striosome markers among MSN-D1 subtypes. The subtypes MSN-D1 1-5 are hypomethylated in these genes, indicating they are likely from the striosome compartment of striatum. (D) Distribution of Pearson correlations between CG-methylation levels of DMRs and CH-methylation levels of genes. Consideration of differentiation of gene body methylation and DNA loops greatly improves the association between DMRs and genes. (E) Distribution of SYT1 expressions in MSN-D1 and L2/3-IT. L2/3-IT cells have high expression levels.


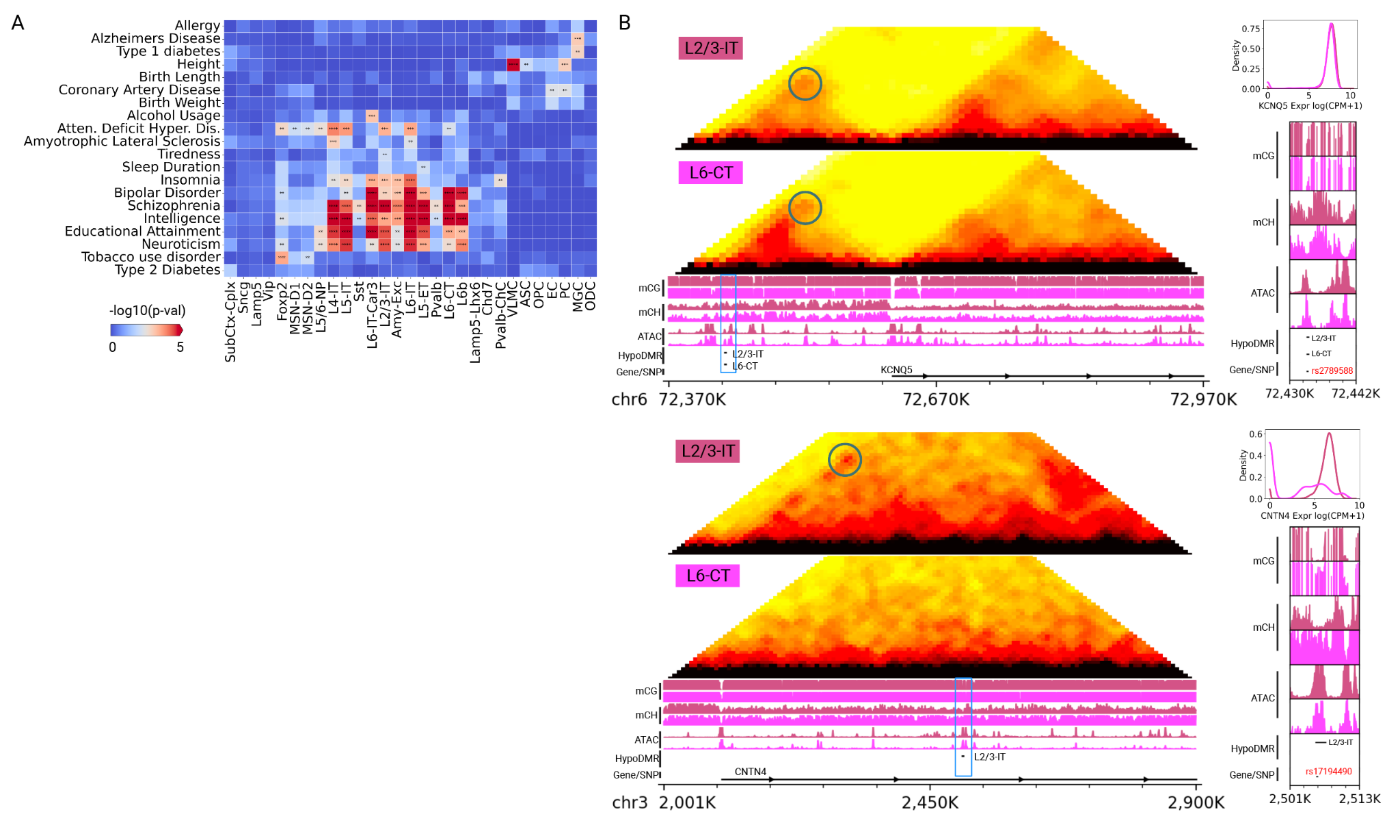


**Figure S15. Brain disorder risk variant enrichment.** (A) Heatmap showing the results of linkage disequilibrium score regression analysis of the variants associated with the indicated traits or diseases in loop-overlapped DMRs identified from human major cell types. (B) Examples of complexity and heterogeneity of risk variants overlapping DMRs and corresponding chromatin conformation, DNA methylation, chromatin accessibility, and gene expression levels. Upper panel, risk variant rs2789588 and gene KCNQ5. Lower panel, risk variant rs17194490 and gene CNTN4. In each subfigure, the circle(s) in the left panel denotes the implicated loops, the lower right panel shows the zoom-in view of the blue boxed regions, and the upper right panel shows the expression distribution of the corresponding genes..


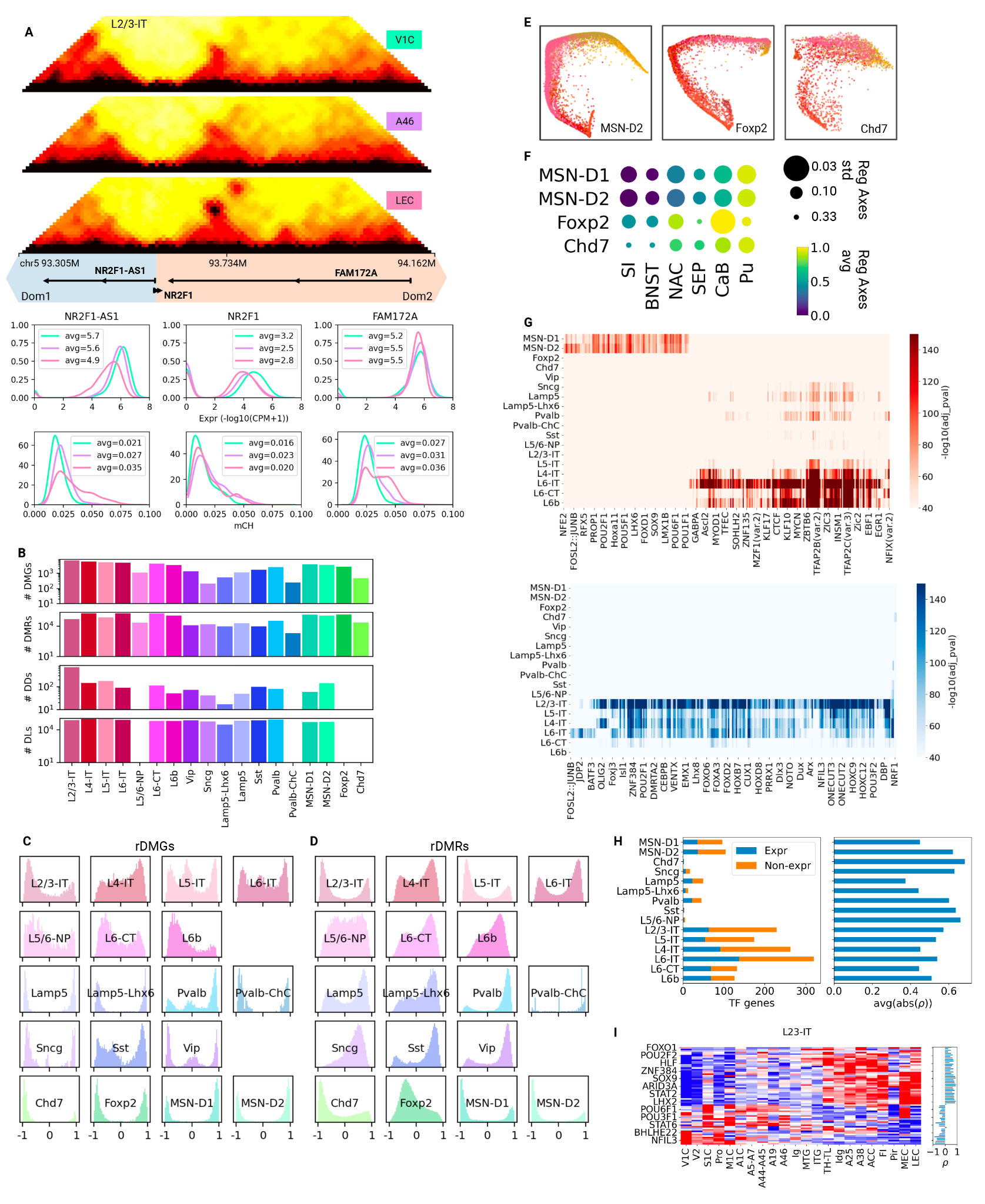


**Figure S16. Regional axes of cortical and subcortical cells.** (A) The gene NR2F1 has higher expression levels in L2/3-IT cells from V1C and LEC than A46. (B) It also has concordant lower CH-methylation levels in L2/3-IT cells from V1C and LEC than A46. (C) Chromatin conformation around the gene NR2F1 shows gradient changes in domain and loop strength. The two associated chromatin domains change in opposite directions. (D) Zoom-in view of example differential-loop-overlapping rDMRs from the “increasing” domain. The methylation levels decrease from V1C to A46 to LEC. (E) The number of regionally differential features is shown in bar plots. (F, G) Distributions of Pearson correlations between methylation levels of rDMG (CH, left) or rDMR(CG, right) and regional axes determined in cortical regions (left) and basal ganglia (right), respectively. Considerable features show methylation gradients along the axes, manifested by Pearson correlations close to -1 or 1. (H) The embedding of major types MSN-D2, Foxp2, and Chd7 in regional spaces colored by dissection regions show that they share similar regional axes. (I) The consensus regional axis of basal ganglia was constructed in the same way as in Fig 4C.


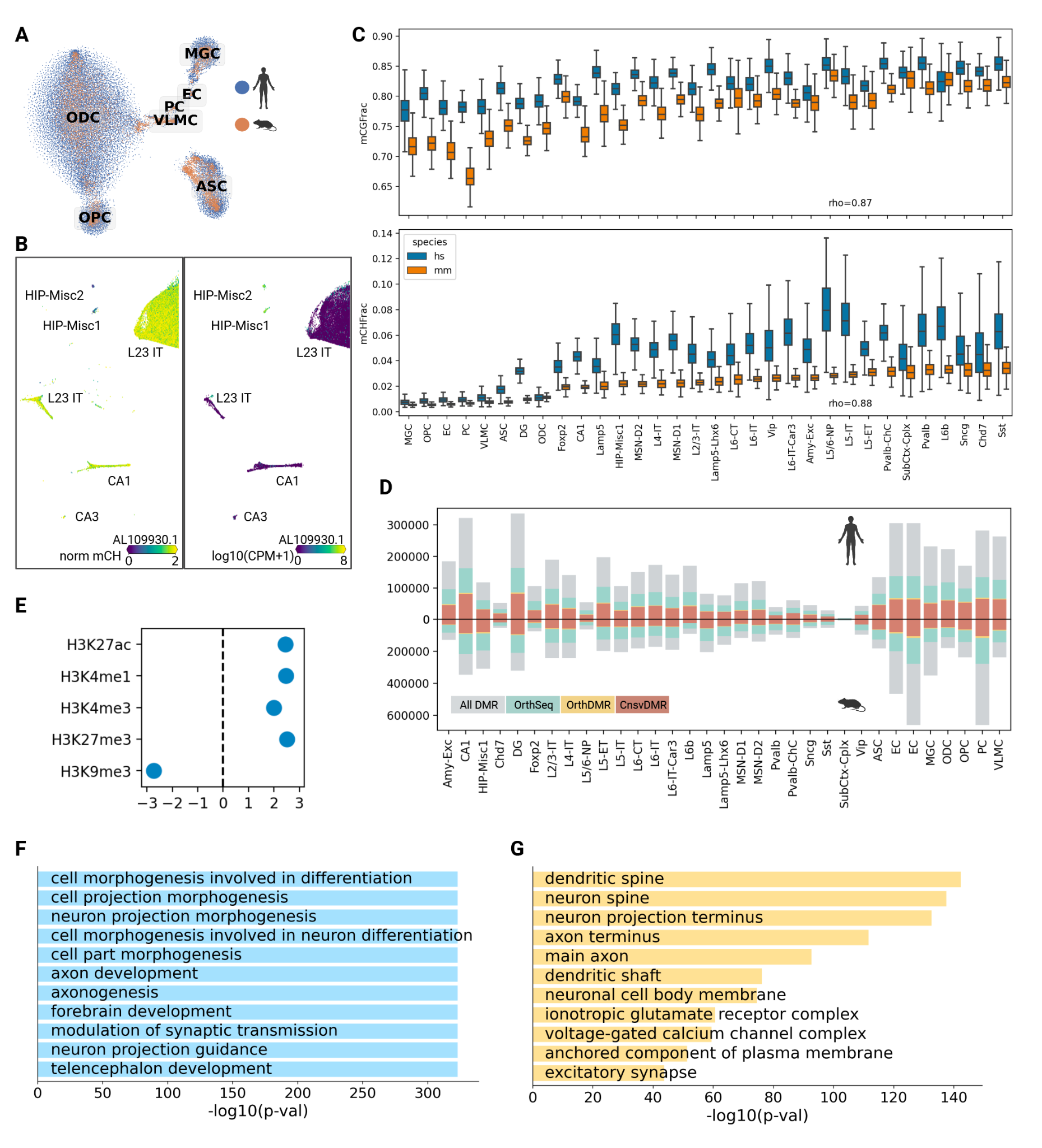


**Figure S17. Cross-species comparison between human and mouse brain cell methylomes.** (A) Integration of single-cell methylomes between human and mouse brains shows cell type conservation across species in non-neurons. (B) The cell types of HIP-Misc1 and HIP-Misc2 both feature CH-hypomethylation and gene expression of lncRNA AL109930.1. (C) Boxplots show a detailed comparison of global CG- and CH-methylation levels of conserved cell types between the human and mouse. (D) Cell type-specific numbers of DMRs in different cross-species matching categories. (E) Comparison to histone modification marks in mouse forebrains shows that the hcCnsvDMRs are depleted from heterochromatic regions (H3K9me3) as well as enriched in regions of enhancers (H3K27ac & H3K4me1), promoters (H3K4me3), and poised enhancers (H3K27m3) (F, G) GO term enrichment analysis show that hcCnsvDMRs are highly enriched in biological processes related to forebrain development (F) and in cellular components related to dendrites and synapses (G).


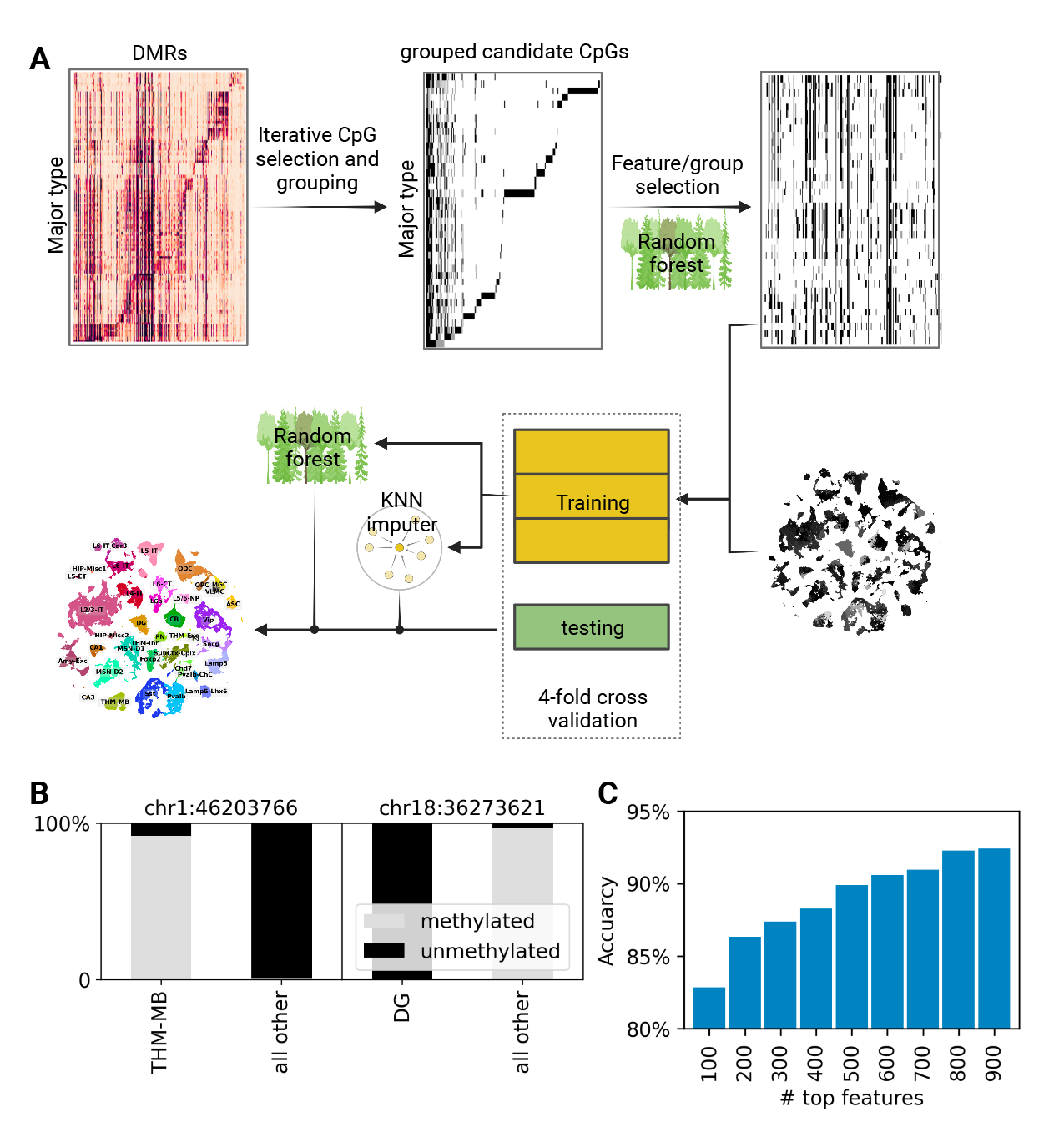


**Figure S18. snMCodes for brain cell types.** (A) Detailed workflow of the derivation of snMCodes (Methods). (B) Example of highly cell-type-specific differentially methylated CpG sites. (C) Prediction accuracy of snMCodes increases with the number of features used, which show saturation around 800~900.

Captions for Supplementary tables

**Table S1.**

Information of samples

**Table S2.**

Brain regions and abbreviation

**Table S3.**

Information of brain donors

**Table S4.**

Cell type taxonomy

**Table S5.**

Cell meta information and annotation of single nuclei profiled with snmC-seq3 and snm3C-seq

**Table S6.**

Differential loop between major types

**Table S7.**

Candidate Cis-Regulatory Elements

**Table S8.**

CpG sites and groups of scMCodes

**Table S9.**

Methylation status of feature groups of scMCodes

**References**

1. [H. Liu, J. Zhou, W. Tian, C. Luo, A. Bartlett, A. Aldridge, J. Lucero, J. K. Osteen, J. R. Nery, H. Chen, A. Rivkin, R. G. Castanon, B. Clock, Y. E. Li, X. Hou, O. B. Poirion, S. Preissl, A. Pinto-Duarte, C. O’Connor, L. Boggeman, C. Fitzpatrick, M. Nunn, E. A. Mukamel, Z. Zhang, E. M. Callaway, B. Ren, J. R. Dixon, M. M. Behrens, J. R. Ecker, DNA methylation atlas of the mouse brain at single-cell resolution. *Nature*. **598**, 120–128 (2021).](http://paperpile.com/b/cCKYZi/x536h)

2. [Y. E. Li, S. Preissl, X. Hou, Z. Zhang, K. Zhang, Y. Qiu, O. B. Poirion, B. Li, J. Chiou, H. Liu, A. Pinto-Duarte, N. Kubo, X. Yang, R. Fang, X. Wang, J. Y. Han, J. Lucero, Y. Yan, M. Miller, S. Kuan, D. Gorkin, K. J. Gaulton, Y. Shen, M. Nunn, E. A. Mukamel, M. M. Behrens, J. R. Ecker, B. Ren, An atlas of gene regulatory elements in adult mouse cerebrum. *Nature*. **598**, 129–136 (2021).](http://paperpile.com/b/cCKYZi/O9Ftg)

3. [D.-S. Lee, C. Luo, J. Zhou, S. Chandran, A. Rivkin, A. Bartlett, J. R. Nery, C. Fitzpatrick, C. O’Connor, J. R. Dixon, J. R. Ecker, Simultaneous profiling of 3D genome structure and DNA methylation in single human cells. *Nat. Methods*. **16**, 999–1006 (2019).](http://paperpile.com/b/cCKYZi/lgFpv)

4. [Y. He, M. Hariharan, D. U. Gorkin, D. E. Dickel, C. Luo, R. G. Castanon, J. R. Nery, A. Y. Lee, Y. Zhao, H. Huang, B. A. Williams, D. Trout, H. Amrhein, R. Fang, H. Chen, B. Li, A. Visel, L. A. Pennacchio, B. Ren, J. R. Ecker, Spatiotemporal DNA methylome dynamics of the developing mouse fetus. *Nature*. **583**, 752–759 (2020).](http://paperpile.com/b/cCKYZi/n3FgY)

5. [C. Luo, C. L. Keown, L. Kurihara, J. Zhou, Y. He, J. Li, R. Castanon, J. Lucero, J. R. Nery, J. P. Sandoval, B. Bui, T. J. Sejnowski, T. T. Harkins, E. A. Mukamel, M. M. Behrens, J. R. Ecker, Single-cell methylomes identify neuronal subtypes and regulatory elements in mammalian cortex. *Science*. **357**, 600–604 (2017).](http://paperpile.com/b/cCKYZi/DdVIi)

6. [A. de Mendoza, D. Poppe, S. Buckberry, J. Pflueger, C. B. Albertin, T. Daish, S. Bertrand, E. de la Calle-Mustienes, J. L. Gómez-Skarmeta, J. R. Nery, J. R. Ecker, B. Baer, C. W. Ragsdale, F. Grützner, H. Escriva, B. Venkatesh, O. Bogdanovic, R. Lister, The emergence of the brain non-CpG methylation system in vertebrates. *Nat Ecol Evol*. **5**, 369–378 (2021).](http://paperpile.com/b/cCKYZi/M88MS)

7. [R. Lister, E. A. Mukamel, J. R. Nery, M. Urich, C. A. Puddifoot, N. D. Johnson, J. Lucero, Y. Huang, A. J. Dwork, M. D. Schultz, M. Yu, J. Tonti-Filippini, H. Heyn, S. Hu, J. C. Wu, A. Rao, M. Esteller, C. He, F. G. Haghighi, T. J. Sejnowski, M. M. Behrens, J. R. Ecker, Global epigenomic reconfiguration during mammalian brain development. *Science*. **341**, 1237905 (2013).](http://paperpile.com/b/cCKYZi/8tPb1)

8. [R. Tillotson, J. Cholewa-Waclaw, K. Chhatbar, J. C. Connelly, S. A. Kirschner, S. Webb, M. V. Koerner, J. Selfridge, D. A. Kelly, D. De Sousa, K. Brown, M. J. Lyst, S. Kriaucionis, A. Bird, Neuronal non-CG methylation is an essential target for MeCP2 function. *Mol. Cell*. **81**, 1260–1275.e12 (2021).](http://paperpile.com/b/cCKYZi/NfOef)

9. [J. Dekker, L. Mirny, The 3D Genome as Moderator of Chromosomal Communication. *Cell*. **164**, 1110–1121 (2016).](http://paperpile.com/b/cCKYZi/a9vXG)

10. [C. Luo, A. Rivkin, J. Zhou, J. P. Sandoval, L. Kurihara, J. Lucero, R. Castanon, J. R. Nery, A. Pinto-Duarte, B. Bui, C. Fitzpatrick, C. O’Connor, S. Ruga, M. E. Van Eden, D. A. Davis, D. C. Mash, M. M. Behrens, J. R. Ecker, Robust single-cell DNA methylome profiling with snmC-seq2. *Nat. Commun.* **9**, 3824 (2018).](http://paperpile.com/b/cCKYZi/DN4Wq)

11. [K. Siletti, R. Hodge, A. M. Albiach, L. Hu, K. W. Lee, P. Lönnerberg, T. Bakken, S.-L. Ding, M. Clark, T. Casper, N. Dee, J. Gloe, C. Dirk Keene, J. Nyhus, H. Tung, A. M. Yanny, E. Arenas, E. S. Lein, S. Linnarsson, Transcriptomic diversity of cell types across the adult human brain. *bioRxiv* (2022), p. 2022.10.12.511898.](http://paperpile.com/b/cCKYZi/xrySe)

12. [Y. E. Li, S. Preissl, M. Miller, N. D. Johnson, Z. Wang, H. Jiao, C. Zhu, Z. Wang, Y. Xie, O. Poirion, C. Kern, A. Pinto-Duarte, W. Tian, K. Siletti, N. Emerson, J. Osteen, J. Lucero, L. Lin, Q. Yang, Q. Zhu, S. Espinoza, A. M. Yanny, J. Nyhus, N. Dee, T. Casper, N. Shapovalova, D. Hirschstein, R. D. Hodge, S. Linnarsson, T. Bakken, B. Levi, C. Dirk Keene, J. Shang, E. S. Lein, A. Wang, M. Margarita Behrens, J. R. Ecker, B. Ren, A comparative atlas of single-cell chromatin accessibility in the human brain. *bioRxiv* (2022), p. 2022.11.09.515833.](http://paperpile.com/b/cCKYZi/lXwyJ)

13. [J. Feng, Y. Zhou, S. L. Campbell, T. Le, E. Li, J. D. Sweatt, A. J. Silva, G. Fan, Dnmt1 and Dnmt3a maintain DNA methylation and regulate synaptic function in adult forebrain neurons. *Nat. Neurosci.* **13**, 423–430 (2010).](http://paperpile.com/b/cCKYZi/lcuEu)

14. [Y. He, J. R. Ecker, Non-CG Methylation in the Human Genome. *Annu. Rev. Genomics Hum. Genet.* **16**, 55–77 (2015).](http://paperpile.com/b/cCKYZi/yZgEY)

15. [J. Zhou, J. Ma, Y. Chen, C. Cheng, B. Bao, J. Peng, T. J. Sejnowski, J. R. Dixon, J. R. Ecker, Robust single-cell Hi-C clustering by convolution- and random-walk–based imputation. *Proceedings of the National Academy of Sciences*. **116**, 14011–14018 (2019).](http://paperpile.com/b/cCKYZi/mRpsU)

16. [L. Tan, W. Ma, H. Wu, Y. Zheng, D. Xing, R. Chen, X. Li, N. Daley, K. Deisseroth, X. S. Xie, Changes in genome architecture and transcriptional dynamics progress independently of sensory experience during post-natal brain development. *Cell*. **184**, 741–758.e17 (2021).](http://paperpile.com/b/cCKYZi/GbMPe)

17. [R. D. Hodge, T. E. Bakken, J. A. Miller, K. A. Smith, E. R. Barkan, L. T. Graybuck, J. L. Close, B. Long, N. Johansen, O. Penn, Z. Yao, J. Eggermont, T. Höllt, B. P. Levi, S. I. Shehata, B. Aevermann, A. Beller, D. Bertagnolli, K. Brouner, T. Casper, C. Cobbs, R. Dalley, N. Dee, S.-L. Ding, R. G. Ellenbogen, O. Fong, E. Garren, J. Goldy, R. P. Gwinn, D. Hirschstein, C. D. Keene, M. Keshk, A. L. Ko, K. Lathia, A. Mahfouz, Z. Maltzer, M. McGraw, T. N. Nguyen, J. Nyhus, J. G. Ojemann, A. Oldre, S. Parry, S. Reynolds, C. Rimorin, N. V. Shapovalova, S. Somasundaram, A. Szafer, E. R. Thomsen, M. Tieu, G. Quon, R. H. Scheuermann, R. Yuste, S. M. Sunkin, B. Lelieveldt, D. Feng, L. Ng, A. Bernard, M. Hawrylycz, J. W. Phillips, B. Tasic, H. Zeng, A. R. Jones, C. Koch, E. S. Lein, Conserved cell types with divergent features in human versus mouse cortex. *Nature*. **573**, 61–68 (2019).](http://paperpile.com/b/cCKYZi/IgzFn)

18. [T. Nagano, Y. Lubling, C. Várnai, C. Dudley, W. Leung, Y. Baran, N. Mendelson Cohen, S. Wingett, P. Fraser, A. Tanay, Cell-cycle dynamics of chromosomal organization at single-cell resolution. *Nature*. **547**, 61–67 (2017).](http://paperpile.com/b/cCKYZi/HmaUq)

19. [J. D. Buenrostro, B. Wu, U. M. Litzenburger, D. Ruff, M. L. Gonzales, M. P. Snyder, H. Y. Chang, W. J. Greenleaf, Single-cell chromatin accessibility reveals principles of regulatory variation. *Nature*. **523**, 486–490 (2015).](http://paperpile.com/b/cCKYZi/9pR3M)

20. [R. Zhang, T. Zhou, J. Ma, Multiscale and integrative single-cell Hi-C analysis with Higashi. *Nat. Biotechnol.* **40**, 254–261 (2022).](http://paperpile.com/b/cCKYZi/QH3HP)

21. [R. Zhang, T. Zhou, J. Ma, Ultrafast and interpretable single-cell 3D genome analysis with Fast-Higashi. *Cell Syst*. **13**, 798–807.e6 (2022).](http://paperpile.com/b/cCKYZi/4cLiX)

22. [J. R. Dixon, S. Selvaraj, F. Yue, A. Kim, Y. Li, Y. Shen, M. Hu, J. S. Liu, B. Ren, Topological domains in mammalian genomes identified by analysis of chromatin interactions. *Nature*. **485**, 376–380 (2012).](http://paperpile.com/b/cCKYZi/6UUps)

23. [S. S. P. Rao, M. H. Huntley, N. C. Durand, E. K. Stamenova, I. D. Bochkov, J. T. Robinson, A. L. Sanborn, I. Machol, A. D. Omer, E. S. Lander, E. L. Aiden, A 3D map of the human genome at kilobase resolution reveals principles of chromatin looping. *Cell*. **159**, 1665–1680 (2014).](http://paperpile.com/b/cCKYZi/ljSGF)

24. [J. R. Dixon, I. Jung, S. Selvaraj, Y. Shen, J. E. Antosiewicz-Bourget, A. Y. Lee, Z. Ye, A. Kim, N. Rajagopal, W. Xie, Y. Diao, J. Liang, H. Zhao, V. V. Lobanenkov, J. R. Ecker, J. A. Thomson, B. Ren, Chromatin architecture reorganization during stem cell differentiation. *Nature*. **518**, 331–336 (2015).](http://paperpile.com/b/cCKYZi/GtqCA)

25. [A. D. Schmitt, M. Hu, I. Jung, Z. Xu, Y. Qiu, C. L. Tan, Y. Li, S. Lin, Y. Lin, C. L. Barr, B. Ren, A Compendium of Chromatin Contact Maps Reveals Spatially Active Regions in the Human Genome. *Cell Rep.* **17**, 2042–2059 (2016).](http://paperpile.com/b/cCKYZi/fCqMw)

26. [B. Bonev, N. Mendelson Cohen, Q. Szabo, L. Fritsch, G. L. Papadopoulos, Y. Lubling, X. Xu, X. Lv, J.-P. Hugnot, A. Tanay, G. Cavalli, Multiscale 3D Genome Rewiring during Mouse Neural Development. *Cell*. **171**, 557–572.e24 (2017).](http://paperpile.com/b/cCKYZi/vteK4)

27. [Y. Zhang, T. Li, S. Preissl, M. L. Amaral, J. D. Grinstein, E. N. Farah, E. Destici, Y. Qiu, R. Hu, A. Y. Lee, S. Chee, K. Ma, Z. Ye, Q. Zhu, H. Huang, R. Fang, L. Yu, J. C. Izpisua Belmonte, J. Wu, S. M. Evans, N. C. Chi, B. Ren, Transcriptionally active HERV-H retrotransposons demarcate topologically associating domains in human pluripotent stem cells. *Nat. Genet.* **51**, 1380–1388 (2019).](http://paperpile.com/b/cCKYZi/34z7w)

28. [W. Winick-Ng, A. Kukalev, I. Harabula, L. Zea-Redondo, D. Szabó, M. Meijer, L. Serebreni, Y. Zhang, S. Bianco, A. M. Chiariello, I. Irastorza-Azcarate, C. J. Thieme, T. M. Sparks, S. Carvalho, L. Fiorillo, F. Musella, E. Irani, E. T. Triglia, A. A. Kolodziejczyk, A. Abentung, G. Apostolova, E. J. Paul, V. Franke, R. Kempfer, A. Akalin, S. A. Teichmann, G. Dechant, M. A. Ungless, M. Nicodemi, L. Welch, G. Castelo-Branco, A. Pombo, Cell-type specialization is encoded by specific chromatin topologies. *Nature*, 1–8 (2021).](http://paperpile.com/b/cCKYZi/jdVpI)

29. [L. Wiehle, G. J. Thorn, G. Raddatz, C. T. Clarkson, K. Rippe, F. Lyko, A. Breiling, V. B. Teif, DNA (de)methylation in embryonic stem cells controls CTCF-dependent chromatin boundaries. *Genome Res.* **29**, 750–761 (2019).](http://paperpile.com/b/cCKYZi/ydqnv)

30. [F. Noack, S. Vangelisti, G. Raffl, M. Carido, J. Diwakar, F. Chong, B. Bonev, Multimodal profiling of the transcriptional regulatory landscape of the developing mouse cortex identifies Neurog2 as a key epigenome remodeler. *Nat. Neurosci.* **25**, 154–167 (2022).](http://paperpile.com/b/cCKYZi/smLm1)

31. [A. Scelfo, V. Barra, N. Abdennur, G. Spracklin, F. Busato, C. Salinas-Luypaert, E. Bonaiti, G. Velasco, A. Chipont, C. Guérin, A. E. Tijhuis, D. C. J. Spierings, C. Francastel, F. Foijer, J. Tost, L. Mirny, D. Fachinetti, Tunable DNMT1 degradation reveals cooperation of DNMT1 and DNMT3B in regulating DNA methylation dynamics and genome organization. *bioRxiv* (2023), p. 2023.05.04.539406.](http://paperpile.com/b/cCKYZi/i60nA)

32. [A. Mo, E. A. Mukamel, F. P. Davis, C. Luo, G. L. Henry, S. Picard, M. A. Urich, J. R. Nery, T. J. Sejnowski, R. Lister, S. R. Eddy, J. R. Ecker, J. Nathans, Epigenomic Signatures of Neuronal Diversity in the Mammalian Brain. *Neuron*. **86**, 1369–1384 (2015).](http://paperpile.com/b/cCKYZi/yvzor)

33. [W. E. Heavner, S. Ji, J. H. Notwell, E. S. Dyer, A. M. Tseng, J. Birgmeier, B. Yoo, G. Bejerano, S. K. McConnell, Transcription factor expression defines subclasses of developing projection neurons highly similar to single-cell RNA-seq subtypes. *Proc. Natl. Acad. Sci. U. S. A.* **117**, 25074–25084 (2020).](http://paperpile.com/b/cCKYZi/G1xxs)

34. [S. Warming, R. A. Rachel, N. A. Jenkins, N. G. Copeland, Zfp423 is required for normal cerebellar development. *Mol. Cell. Biol.* **26**, 6913–6922 (2006).](http://paperpile.com/b/cCKYZi/7QilS)

35. [F. Casoni, L. Croci, C. Bosone, R. D’Ambrosio, A. Badaloni, D. Gaudesi, V. Barili, J. R. Sarna, L. Tessarollo, O. Cremona, R. Hawkes, S. Warming, G. G. Consalez, Zfp423/ZNF423 regulates cell cycle progression, the mode of cell division and the DNA-damage response in Purkinje neuron progenitors. *Development*. **144**, 3686–3697 (2017).](http://paperpile.com/b/cCKYZi/Ve1L3)

36. [L. Croci, S.-H. Chung, G. Masserdotti, S. Gianola, A. Bizzoca, G. Gennarini, A. Corradi, F. Rossi, R. Hawkes, G. G. Consalez, A key role for the HLH transcription factor EBF2COE2,O/E-3 in Purkinje neuron migration and cerebellar cortical topography. *Development*. **133**, 2719–2729 (2006).](http://paperpile.com/b/cCKYZi/N9zWu)

37. [K. Takahashi, F.-C. Liu, T. Oishi, T. Mori, N. Higo, M. Hayashi, K. Hirokawa, H. Takahashi, Expression of FOXP2 in the developing monkey forebrain: comparison with the expression of the genes FOXP1, PBX3, and MEIS2. *J. Comp. Neurol.* **509**, 180–189 (2008).](http://paperpile.com/b/cCKYZi/lQoML)

38. [T. Kaoru, F.-C. Liu, M. Ishida, T. Oishi, M. Hayashi, M. Kitagawa, K. Shimoda, H. Takahashi, Molecular characterization of the intercalated cell masses of the amygdala: implications for the relationship with the striatum. *Neuroscience*. **166**, 220–230 (2010).](http://paperpile.com/b/cCKYZi/cjwUk)

39. [J. Nasser, D. T. Bergman, C. P. Fulco, P. Guckelberger, B. R. Doughty, T. A. Patwardhan, T. R. Jones, T. H. Nguyen, J. C. Ulirsch, F. Lekschas, K. Mualim, H. M. Natri, E. M. Weeks, G. Munson, M. Kane, H. Y. Kang, A. Cui, J. P. Ray, T. M. Eisenhaure, R. L. Collins, K. Dey, H. Pfister, A. L. Price, C. B. Epstein, A. Kundaje, R. J. Xavier, M. J. Daly, H. Huang, H. K. Finucane, N. Hacohen, E. S. Lander, J. M. Engreitz, Genome-wide enhancer maps link risk variants to disease genes. *Nature*. **593**, 238–243 (2021).](http://paperpile.com/b/cCKYZi/9WBC9)

40. [B. K. Bulik-Sullivan, P.-R. Loh, H. K. Finucane, S. Ripke, J. Yang, Schizophrenia Working Group of the Psychiatric Genomics Consortium, N. Patterson, M. J. Daly, A. L. Price, B. M. Neale, LD Score regression distinguishes confounding from polygenicity in genome-wide association studies. *Nat. Genet.* **47**, 291–295 (2015).](http://paperpile.com/b/cCKYZi/vgdPQ)

41. [D. V. Hansen, J. E. Hanson, M. Sheng, Microglia in Alzheimer’s disease. *J. Cell Biol.* **217**, 459–472 (2018).](http://paperpile.com/b/cCKYZi/jXIMV)

42. [M. Liu, Y. Jiang, R. Wedow, Y. Li, D. M. Brazel, F. Chen, G. Datta, J. Davila-Velderrain, D. McGuire, C. Tian, X. Zhan, 23andMe Research Team, HUNT All-In Psychiatry, H. Choquet, A. R. Docherty, J. D. Faul, J. R. Foerster, L. G. Fritsche, M. E. Gabrielsen, S. D. Gordon, J. Haessler, J.-J. Hottenga, H. Huang, S.-K. Jang, P. R. Jansen, Y. Ling, R. Mägi, N. Matoba, G. McMahon, A. Mulas, V. Orrù, T. Palviainen, A. Pandit, G. W. Reginsson, A. H. Skogholt, J. A. Smith, A. E. Taylor, C. Turman, G. Willemsen, H. Young, K. A. Young, G. J. M. Zajac, W. Zhao, W. Zhou, G. Bjornsdottir, J. D. Boardman, M. Boehnke, D. I. Boomsma, C. Chen, F. Cucca, G. E. Davies, C. B. Eaton, M. A. Ehringer, T. Esko, E. Fiorillo, N. A. Gillespie, D. F. Gudbjartsson, T. Haller, K. M. Harris, A. C. Heath, J. K. Hewitt, I. B. Hickie, J. E. Hokanson, C. J. Hopfer, D. J. Hunter, W. G. Iacono, E. O. Johnson, Y. Kamatani, S. L. R. Kardia, M. C. Keller, M. Kellis, C. Kooperberg, P. Kraft, K. S. Krauter, M. Laakso, P. A. Lind, A. Loukola, S. M. Lutz, P. A. F. Madden, N. G. Martin, M. McGue, M. B. McQueen, S. E. Medland, A. Metspalu, K. L. Mohlke, J. B. Nielsen, Y. Okada, U. Peters, T. J. C. Polderman, D. Posthuma, A. P. Reiner, J. P. Rice, E. Rimm, R. J. Rose, V. Runarsdottir, M. C. Stallings, A. Stančáková, H. Stefansson, K. K. Thai, H. A. Tindle, T. Tyrfingsson, T. L. Wall, D. R. Weir, C. Weisner, J. B. Whitfield, B. S. Winsvold, J. Yin, L. Zuccolo, L. J. Bierut, K. Hveem, J. J. Lee, M. R. Munafò, N. L. Saccone, C. J. Willer, M. C. Cornelis, S. P. David, D. A. Hinds, E. Jorgenson, J. Kaprio, J. A. Stitzel, K. Stefansson, T. E. Thorgeirsson, G. Abecasis, D. J. Liu, S. Vrieze, Association studies of up to 1.2 million individuals yield new insights into the genetic etiology of tobacco and alcohol use. *Nat. Genet.* **51**, 237–244 (2019).](http://paperpile.com/b/cCKYZi/z0ddl)

43. [S. N. Sansom, F. J. Livesey, Gradients in the brain: the control of the development of form and function in the cerebral cortex. *Cold Spring Harb. Perspect. Biol.* **1**, a002519 (2009).](http://paperpile.com/b/cCKYZi/8PihW)

44. [M. J. Hawrylycz, E. S. Lein, A. L. Guillozet-Bongaarts, E. H. Shen, L. Ng, J. A. Miller, L. N. van de Lagemaat, K. A. Smith, A. Ebbert, Z. L. Riley, C. Abajian, C. F. Beckmann, A. Bernard, D. Bertagnolli, A. F. Boe, P. M. Cartagena, M. M. Chakravarty, M. Chapin, J. Chong, R. A. Dalley, B. David Daly, C. Dang, S. Datta, N. Dee, T. A. Dolbeare, V. Faber, D. Feng, D. R. Fowler, J. Goldy, B. W. Gregor, Z. Haradon, D. R. Haynor, J. G. Hohmann, S. Horvath, R. E. Howard, A. Jeromin, J. M. Jochim, M. Kinnunen, C. Lau, E. T. Lazarz, C. Lee, T. A. Lemon, L. Li, Y. Li, J. A. Morris, C. C. Overly, P. D. Parker, S. E. Parry, M. Reding, J. J. Royall, J. Schulkin, P. A. Sequeira, C. R. Slaughterbeck, S. C. Smith, A. J. Sodt, S. M. Sunkin, B. E. Swanson, M. P. Vawter, D. Williams, P. Wohnoutka, H. R. Zielke, D. H. Geschwind, P. R. Hof, S. M. Smith, C. Koch, S. G. N. Grant, A. R. Jones, An anatomically comprehensive atlas of the adult human brain transcriptome. *Nature*. **489**, 391–399 (2012).](http://paperpile.com/b/cCKYZi/Uw7VF)

45. [Z. Yao, H. Liu, F. Xie, S. Fischer, R. S. Adkins, A. I. Aldridge, S. A. Ament, A. Bartlett, M. M. Behrens, K. Van den Berge, D. Bertagnolli, H. R. de Bézieux, T. Biancalani, A. S. Booeshaghi, H. C. Bravo, T. Casper, C. Colantuoni, J. Crabtree, H. Creasy, K. Crichton, M. Crow, N. Dee, E. L. Dougherty, W. I. Doyle, S. Dudoit, R. Fang, V. Felix, O. Fong, M. Giglio, J. Goldy, M. Hawrylycz, B. R. Herb, R. Hertzano, X. Hou, Q. Hu, J. Kancherla, M. Kroll, K. Lathia, Y. E. Li, J. D. Lucero, C. Luo, A. Mahurkar, D. McMillen, N. M. Nadaf, J. R. Nery, T. N. Nguyen, S.-Y. Niu, V. Ntranos, J. Orvis, J. K. Osteen, T. Pham, A. Pinto-Duarte, O. Poirion, S. Preissl, E. Purdom, C. Rimorin, D. Risso, A. C. Rivkin, K. Smith, K. Street, J. Sulc, V. Svensson, M. Tieu, A. Torkelson, H. Tung, E. D. Vaishnav, C. R. Vanderburg, C. van Velthoven, X. Wang, O. R. White, Z. J. Huang, P. V. Kharchenko, L. Pachter, J. Ngai, A. Regev, B. Tasic, J. D. Welch, J. Gillis, E. Z. Macosko, B. Ren, J. R. Ecker, H. Zeng, E. A. Mukamel, A transcriptomic and epigenomic cell atlas of the mouse primary motor cortex. *Nature*. **598**, 103–110 (2021).](http://paperpile.com/b/cCKYZi/cEem0)

46. [F. M. Krienen, M. Goldman, Q. Zhang, R. C H Del Rosario, M. Florio, R. Machold, A. Saunders, K. Levandowski, H. Zaniewski, B. Schuman, C. Wu, A. Lutservitz, C. D. Mullally, N. Reed, E. Bien, L. Bortolin, M. Fernandez-Otero, J. D. Lin, A. Wysoker, J. Nemesh, D. Kulp, M. Burns, V. Tkachev, R. Smith, C. A. Walsh, J. Dimidschstein, B. Rudy, L. S Kean, S. Berretta, G. Fishell, G. Feng, S. A. McCarroll, Innovations present in the primate interneuron repertoire. *Nature*. **586**, 262–269 (2020).](http://paperpile.com/b/cCKYZi/7js67)

47. [Z. Yao, C. T. J. van Velthoven, T. N. Nguyen, J. Goldy, A. E. Sedeno-Cortes, F. Baftizadeh, D. Bertagnolli, T. Casper, M. Chiang, K. Crichton, S.-L. Ding, O. Fong, E. Garren, A. Glandon, N. W. Gouwens, J. Gray, L. T. Graybuck, M. J. Hawrylycz, D. Hirschstein, M. Kroll, K. Lathia, C. Lee, B. Levi, D. McMillen, S. Mok, T. Pham, Q. Ren, C. Rimorin, N. Shapovalova, J. Sulc, S. M. Sunkin, M. Tieu, A. Torkelson, H. Tung, K. Ward, N. Dee, K. A. Smith, B. Tasic, H. Zeng, A taxonomy of transcriptomic cell types across the isocortex and hippocampal formation. *Cell*. **184**, 3222–3241.e26 (2021).](http://paperpile.com/b/cCKYZi/SnTaD)

48. [B. Tasic, Z. Yao, L. T. Graybuck, K. A. Smith, T. N. Nguyen, D. Bertagnolli, J. Goldy, E. Garren, M. N. Economo, S. Viswanathan, O. Penn, T. Bakken, V. Menon, J. Miller, O. Fong, K. E. Hirokawa, K. Lathia, C. Rimorin, M. Tieu, R. Larsen, T. Casper, E. Barkan, M. Kroll, S. Parry, N. V. Shapovalova, D. Hirschstein, J. Pendergraft, H. A. Sullivan, T. K. Kim, A. Szafer, N. Dee, P. Groblewski, I. Wickersham, A. Cetin, J. A. Harris, B. P. Levi, S. M. Sunkin, L. Madisen, T. L. Daigle, L. Looger, A. Bernard, J. Phillips, E. Lein, M. Hawrylycz, K. Svoboda, A. R. Jones, C. Koch, H. Zeng, Shared and distinct transcriptomic cell types across neocortical areas. *Nature*. **563**, 72–78 (2018).](http://paperpile.com/b/cCKYZi/cFuq1)

49. [H. Chen, L. Albergante, J. Y. Hsu, C. A. Lareau, G. Lo Bosco, J. Guan, S. Zhou, A. N. Gorban, D. E. Bauer, M. J. Aryee, D. M. Langenau, A. Zinovyev, J. D. Buenrostro, G.-C. Yuan, L. Pinello, Single-cell trajectories reconstruction, exploration and mapping of omics data with STREAM. *Nature Communications*. **10** (2019), , doi:](http://paperpile.com/b/cCKYZi/vSFqh)[10.1038/s41467-019-09670-4](http://dx.doi.org/10.1038/s41467-019-09670-4)[.](http://paperpile.com/b/cCKYZi/vSFqh)

50. [J. Feng, W.-H. Hsu, D. Patterson, C.-S. Tseng, H.-W. Hsing, Z.-H. Zhuang, Y.-T. Huang, A. Faedo, J. L. Rubenstein, J. Touboul, S.-J. Chou, COUP-TFI specifies the medial entorhinal cortex identity and induces differential cell adhesion to determine the integrity of its boundary with neocortex. *Sci Adv*. **7** (2021), doi:](http://paperpile.com/b/cCKYZi/TiNEp)[10.1126/sciadv.abf6808](http://dx.doi.org/10.1126/sciadv.abf6808)[.](http://paperpile.com/b/cCKYZi/TiNEp)

51. [G. E. Alexander, M. R. DeLong, P. L. Strick, Parallel organization of functionally segregated circuits linking basal ganglia and cortex. *Annu. Rev. Neurosci.* **9**, 357–381 (1986).](http://paperpile.com/b/cCKYZi/zSk24)

52. [F. A. Middleton, P. L. Strick, Basal ganglia and cerebellar loops: motor and cognitive circuits. *Brain Res. Brain Res. Rev.* **31**, 236–250 (2000).](http://paperpile.com/b/cCKYZi/qY4Q1)

53. [T. E. Bakken, N. L. Jorstad, Q. Hu, B. B. Lake, W. Tian, B. E. Kalmbach, M. Crow, R. D. Hodge, F. M. Krienen, S. A. Sorensen, J. Eggermont, Z. Yao, B. D. Aevermann, A. I. Aldridge, A. Bartlett, D. Bertagnolli, T. Casper, R. G. Castanon, K. Crichton, T. L. Daigle, R. Dalley, N. Dee, N. Dembrow, D. Diep, S.-L. Ding, W. Dong, R. Fang, S. Fischer, M. Goldman, J. Goldy, L. T. Graybuck, B. R. Herb, X. Hou, J. Kancherla, M. Kroll, K. Lathia, B. van Lew, Y. E. Li, C. S. Liu, H. Liu, J. D. Lucero, A. Mahurkar, D. McMillen, J. A. Miller, M. Moussa, J. R. Nery, P. R. Nicovich, S.-Y. Niu, J. Orvis, J. K. Osteen, S. Owen, C. R. Palmer, T. Pham, N. Plongthongkum, O. Poirion, N. M. Reed, C. Rimorin, A. Rivkin, W. J. Romanow, A. E. Sedeño-Cortés, K. Siletti, S. Somasundaram, J. Sulc, M. Tieu, A. Torkelson, H. Tung, X. Wang, F. Xie, A. M. Yanny, R. Zhang, S. A. Ament, M. M. Behrens, H. C. Bravo, J. Chun, A. Dobin, J. Gillis, R. Hertzano, P. R. Hof, T. Höllt, G. D. Horwitz, C. D. Keene, P. V. Kharchenko, A. L. Ko, B. P. Lelieveldt, C. Luo, E. A. Mukamel, A. Pinto-Duarte, S. Preissl, A. Regev, B. Ren, R. H. Scheuermann, K. Smith, W. J. Spain, O. R. White, C. Koch, M. Hawrylycz, B. Tasic, E. Z. Macosko, S. A. McCarroll, J. T. Ting, H. Zeng, K. Zhang, G. Feng, J. R. Ecker, S. Linnarsson, E. S. Lein, Comparative cellular analysis of motor cortex in human, marmoset and mouse. *Nature*. **598**, 111–119 (2021).](http://paperpile.com/b/cCKYZi/hU8ch)

54. [D. Vormstein-Schneider, J. D. Lin, K. A. Pelkey, R. Chittajallu, B. Guo, M. A. Arias-Garcia, K. Allaway, S. Sakopoulos, G. Schneider, O. Stevenson, J. Vergara, J. Sharma, Q. Zhang, T. P. Franken, J. Smith, L. A. Ibrahim, K. J. Mastro, E. Sabri, S. Huang, E. Favuzzi, T. Burbridge, Q. Xu, L. Guo, I. Vogel, V. Sanchez, G. A. Saldi, B. L. Gorissen, X. Yuan, K. A. Zaghloul, O. Devinsky, B. L. Sabatini, R. Batista-Brito, J. Reynolds, G. Feng, Z. Fu, C. J. McBain, G. Fishell, J. Dimidschstein, Viral manipulation of functionally distinct interneurons in mice, non-human primates and humans. *Nat. Neurosci.* **23**, 1629–1636 (2020).](http://paperpile.com/b/cCKYZi/r9kUK)

55. [G. C. Hon, N. Rajagopal, Y. Shen, D. F. McCleary, F. Yue, M. D. Dang, B. Ren, Epigenetic memory at embryonic enhancers identified in DNA methylation maps from adult mouse tissues. *Nat. Genet.* **45**, 1198–1206 (2013).](http://paperpile.com/b/cCKYZi/sFy24)

56. [C. Luo, H. Liu, F. Xie, E. J. Armand, K. Siletti, T. E. Bakken, R. Fang, W. I. Doyle, T. Stuart, R. D. Hodge, L. Hu, B.-A. Wang, Z. Zhang, S. Preissl, D.-S. Lee, J. Zhou, S.-Y. Niu, R. Castanon, A. Bartlett, A. Rivkin, X. Wang, J. Lucero, J. R. Nery, D. A. Davis, D. C. Mash, R. Satija, J. R. Dixon, S. Linnarsson, E. Lein, M. Margarita Behrens, B. Ren, E. A. Mukamel, J. R. Ecker, Single nucleus multi-omics identifies human cortical cell regulatory genome diversity. *Cell Genomics*. **2** (2022), p. 100107.](http://paperpile.com/b/cCKYZi/5qC9b)

57. [A. Nott, I. R. Holtman, N. G. Coufal, J. C. M. Schlachetzki, M. Yu, R. Hu, C. Z. Han, M. Pena, J. Xiao, Y. Wu, Z. Keulen, M. P. Pasillas, C. O’Connor, C. K. Nickl, S. T. Schafer, Z. Shen, R. A. Rissman, J. B. Brewer, D. Gosselin, D. D. Gonda, M. L. Levy, M. G. Rosenfeld, G. McVicker, F. H. Gage, B. Ren, C. K. Glass, Brain cell type-specific enhancer-promoter interactome maps and disease-risk association. *Science*. **366**, 1134–1139 (2019).](http://paperpile.com/b/cCKYZi/XaTLh)

58. [B. Hu, H. Won, W. Mah, R. B. Park, B. Kassim, K. Spiess, A. Kozlenkov, C. A. Crowley, S. Pochareddy, PsychENCODE Consortium, Y. Li, S. Dracheva, N. Sestan, S. Akbarian, D. H. Geschwind, Neuronal and glial 3D chromatin architecture informs the cellular etiology of brain disorders. *Nat. Commun.* **12**, 3968 (2021).](http://paperpile.com/b/cCKYZi/GgXa4)

59. [M. Song, M.-P. Pebworth, X. Yang, A. Abnousi, C. Fan, J. Wen, J. D. Rosen, M. N. K. Choudhary, X. Cui, I. R. Jones, S. Bergenholtz, U. C. Eze, I. Juric, B. Li, L. Maliskova, J. Lee, W. Liu, A. A. Pollen, Y. Li, T. Wang, M. Hu, A. R. Kriegstein, Y. Shen, Cell-type-specific 3D epigenomes in the developing human cortex. *Nature*. **587**, 644–649 (2020).](http://paperpile.com/b/cCKYZi/r0Kx4)

60. [S. Horvath, Y. Zhang, P. Langfelder, R. S. Kahn, M. P. M. Boks, K. van Eijk, L. H. van den Berg, R. A. Ophoff, Aging effects on DNA methylation modules in human brain and blood tissue. *Genome Biol.* **13**, R97 (2012).](http://paperpile.com/b/cCKYZi/xtrHy)

61. [C. Gabbutt, R. O. Schenck, D. J. Weisenberger, C. Kimberley, A. Berner, J. Househam, E. Lakatos, M. Robertson-Tessi, I. Martin, R. Patel, S. K. Clark, A. Latchford, C. P. Barnes, S. J. Leedham, A. R. A. Anderson, T. A. Graham, D. Shibata, Fluctuating methylation clocks for cell lineage tracing at high temporal resolution in human tissues. *Nat. Biotechnol.* **40**, 720–730 (2022).](http://paperpile.com/b/cCKYZi/ybywy)

62. [L. A. Salas, J. K. Wiencke, D. C. Koestler, Z. Zhang, B. C. Christensen, K. T. Kelsey, Tracing human stem cell lineage during development using DNA methylation. *Genome Res.* **28**, 1285–1295 (2018).](http://paperpile.com/b/cCKYZi/IWZa1)

63. [E. Gormally, E. Caboux, P. Vineis, P. Hainaut, Circulating free DNA in plasma or serum as biomarker of carcinogenesis: practical aspects and biological significance. *Mutat. Res.* **635**, 105–117 (2007).](http://paperpile.com/b/cCKYZi/qWCYP)

64. [M. Cisneros-Villanueva, L. Hidalgo-Pérez, M. Rios-Romero, A. Cedro-Tanda, C. A. Ruiz-Villavicencio, K. Page, R. Hastings, D. Fernandez-Garcia, R. Allsopp, M. A. Fonseca-Montaño, S. Jimenez-Morales, V. Padilla-Palma, J. A. Shaw, A. Hidalgo-Miranda, Cell-free DNA analysis in current cancer clinical trials: a review. *Br. J. Cancer*. **126**, 391–400 (2022).](http://paperpile.com/b/cCKYZi/ElENS)

65. [S. Chen, Y. Zhou, Y. Chen, J. Gu, fastp: an ultra-fast all-in-one FASTQ preprocessor. *Bioinformatics*. **34**, i884–i890 (2018).](http://paperpile.com/b/cCKYZi/ZNi6u)

66. [H. Li, Aligning sequence reads, clone sequences and assembly contigs with BWA-MEM. *arXiv [q-bio.GN]* (2013), (available at](http://paperpile.com/b/cCKYZi/LYLEX) <http://arxiv.org/abs/1303.3997>[).](http://paperpile.com/b/cCKYZi/LYLEX)

67. [P. Danecek, J. K. Bonfield, J. Liddle, J. Marshall, V. Ohan, M. O. Pollard, A. Whitwham, T. Keane, S. A. McCarthy, R. M. Davies, H. Li, Twelve years of SAMtools and BCFtools. *Gigascience*. **10** (2021), doi:](http://paperpile.com/b/cCKYZi/4UOkZ)[10.1093/gigascience/giab008](http://dx.doi.org/10.1093/gigascience/giab008)[.](http://paperpile.com/b/cCKYZi/4UOkZ)

68. [R. Poplin, V. Ruano-Rubio, M. A. DePristo, T. J. Fennell, M. O. Carneiro, G. A. Van der Auwera, D. E. Kling, L. D. Gauthier, A. Levy-Moonshine, D. Roazen, K. Shakir, J. Thibault, S. Chandran, C. Whelan, M. Lek, S. Gabriel, M. J. Daly, B. Neale, D. G. MacArthur, E. Banks, Scaling accurate genetic variant discovery to tens of thousands of samples. *bioRxiv* (2018), p. 201178.](http://paperpile.com/b/cCKYZi/PwgmZ)

69. [H. M. Amemiya, A. Kundaje, A. P. Boyle, The ENCODE Blacklist: Identification of Problematic Regions of the Genome. *Sci. Rep.* **9**, 9354 (2019).](http://paperpile.com/b/cCKYZi/vJLD5)

70. [I. Korsunsky, N. Millard, J. Fan, K. Slowikowski, F. Zhang, K. Wei, Y. Baglaenko, M. Brenner, P.-R. Loh, S. Raychaudhuri, Fast, sensitive and accurate integration of single-cell data with Harmony. *Nat. Methods*. **16**, 1289–1296 (2019).](http://paperpile.com/b/cCKYZi/FesJa)

71. [B. Hie, B. Bryson, B. Berger, Efficient integration of heterogeneous single-cell transcriptomes using Scanorama. *Nat. Biotechnol.* **37**, 685–691 (2019).](http://paperpile.com/b/cCKYZi/JFCrQ)

72. [M. D. Schultz, Y. He, J. W. Whitaker, M. Hariharan, E. A. Mukamel, D. Leung, N. Rajagopal, J. R. Nery, M. A. Urich, H. Chen, S. Lin, Y. Lin, I. Jung, A. D. Schmitt, S. Selvaraj, B. Ren, T. J. Sejnowski, W. Wang, J. R. Ecker, Human body epigenome maps reveal noncanonical DNA methylation variation. *Nature*. **523**, 212–216 (2015).](http://paperpile.com/b/cCKYZi/ed4os)

73. [O. Fornes, J. A. Castro-Mondragon, A. Khan, R. van der Lee, X. Zhang, P. A. Richmond, B. P. Modi, S. Correard, M. Gheorghe, D. Baranašić, W. Santana-Garcia, G. Tan, J. Chèneby, B. Ballester, F. Parcy, A. Sandelin, B. Lenhard, W. W. Wasserman, A. Mathelier, JASPAR 2020: update of the open-access database of transcription factor binding profiles. *Nucleic Acids Res.* **48**, D87–D92 (2020).](http://paperpile.com/b/cCKYZi/rYUaz)

74. [E. P. Nora, A. Goloborodko, A.-L. Valton, J. H. Gibcus, A. Uebersohn, N. Abdennur, J. Dekker, L. A. Mirny, B. G. Bruneau, Targeted Degradation of CTCF Decouples Local Insulation of Chromosome Domains from Genomic Compartmentalization. *Cell*. **169**, 930–944.e22 (2017).](http://paperpile.com/b/cCKYZi/rsmwd)

75. [A. Chakraborty, J. G. Wang, F. Ay, dcHiC detects differential compartments across multiple Hi-C datasets. *Nat. Commun.* **13**, 6827 (2022).](http://paperpile.com/b/cCKYZi/ddZhh)

76. [H. Shin, Y. Shi, C. Dai, H. Tjong, K. Gong, F. Alber, X. J. Zhou, TopDom: an efficient and deterministic method for identifying topological domains in genomes. *Nucleic Acids Res.* **44**, e70 (2016).](http://paperpile.com/b/cCKYZi/VoD6N)

77. [M. Yu, A. Abnousi, Y. Zhang, G. Li, L. Lee, Z. Chen, R. Fang, T. M. Lagler, Y. Yang, J. Wen, Q. Sun, Y. Li, B. Ren, M. Hu, SnapHiC: a computational pipeline to identify chromatin loops from single-cell Hi-C data. *Nat. Methods*. **18**, 1056–1059 (2021).](http://paperpile.com/b/cCKYZi/aLTg0)

78. [J.-H. Su, P. Zheng, S. S. Kinrot, B. Bintu, X. Zhuang, Genome-Scale Imaging of the 3D Organization and Transcriptional Activity of Chromatin. *Cell*. **182**, 1641–1659.e26 (2020).](http://paperpile.com/b/cCKYZi/NLU5n)

79. [G. Sabarís, I. Laiker, E. Preger-Ben Noon, N. Frankel, Actors with Multiple Roles: Pleiotropic Enhancers and the Paradigm of Enhancer Modularity. *Trends Genet.* **35**, 423–433 (2019).](http://paperpile.com/b/cCKYZi/uAmCc)

80. [J. E. Savage, P. R. Jansen, S. Stringer, K. Watanabe, J. Bryois, C. A. de Leeuw, M. Nagel, S. Awasthi, P. B. Barr, J. R. I. Coleman, K. L. Grasby, A. R. Hammerschlag, J. A. Kaminski, R. Karlsson, E. Krapohl, M. Lam, M. Nygaard, C. A. Reynolds, J. W. Trampush, H. Young, D. Zabaneh, S. Hägg, N. K. Hansell, I. K. Karlsson, S. Linnarsson, G. W. Montgomery, A. B. Muñoz-Manchado, E. B. Quinlan, G. Schumann, N. G. Skene, B. T. Webb, T. White, D. E. Arking, D. Avramopoulos, R. M. Bilder, P. Bitsios, K. E. Burdick, T. D. Cannon, O. Chiba-Falek, A. Christoforou, E. T. Cirulli, E. Congdon, A. Corvin, G. Davies, I. J. Deary, P. DeRosse, D. Dickinson, S. Djurovic, G. Donohoe, E. D. Conley, J. G. Eriksson, T. Espeseth, N. A. Freimer, S. Giakoumaki, I. Giegling, M. Gill, D. C. Glahn, A. R. Hariri, A. Hatzimanolis, M. C. Keller, E. Knowles, D. Koltai, B. Konte, J. Lahti, S. Le Hellard, T. Lencz, D. C. Liewald, E. London, A. J. Lundervold, A. K. Malhotra, I. Melle, D. Morris, A. C. Need, W. Ollier, A. Palotie, A. Payton, N. Pendleton, R. A. Poldrack, K. Räikkönen, I. Reinvang, P. Roussos, D. Rujescu, F. W. Sabb, M. A. Scult, O. B. Smeland, N. Smyrnis, J. M. Starr, V. M. Steen, N. C. Stefanis, R. E. Straub, K. Sundet, H. Tiemeier, A. N. Voineskos, D. R. Weinberger, E. Widen, J. Yu, G. Abecasis, O. A. Andreassen, G. Breen, L. Christiansen, B. Debrabant, D. M. Dick, A. Heinz, J. Hjerling-Leffler, M. A. Ikram, K. S. Kendler, N. G. Martin, S. E. Medland, N. L. Pedersen, R. Plomin, T. J. C. Polderman, S. Ripke, S. van der Sluis, P. F. Sullivan, S. I. Vrieze, M. J. Wright, D. Posthuma, Genome-wide association meta-analysis in 269,867 individuals identifies new genetic and functional links to intelligence. *Nat. Genet.* **50**, 912–919 (2018).](http://paperpile.com/b/cCKYZi/eSujj)

81. [A. Okbay, J. P. Beauchamp, M. A. Fontana, J. J. Lee, T. H. Pers, C. A. Rietveld, P. Turley, G.-B. Chen, V. Emilsson, S. F. W. Meddens, S. Oskarsson, J. K. Pickrell, K. Thom, P. Timshel, R. de Vlaming, A. Abdellaoui, T. S. Ahluwalia, J. Bacelis, C. Baumbach, G. Bjornsdottir, J. H. Brandsma, M. Pina Concas, J. Derringer, N. A. Furlotte, T. E. Galesloot, G. Girotto, R. Gupta, L. M. Hall, S. E. Harris, E. Hofer, M. Horikoshi, J. E. Huffman, K. Kaasik, I. P. Kalafati, R. Karlsson, A. Kong, J. Lahti, S. J. van der Lee, C. deLeeuw, P. A. Lind, K.-O. Lindgren, T. Liu, M. Mangino, J. Marten, E. Mihailov, M. B. Miller, P. J. van der Most, C. Oldmeadow, A. Payton, N. Pervjakova, W. J. Peyrot, Y. Qian, O. Raitakari, R. Rueedi, E. Salvi, B. Schmidt, K. E. Schraut, J. Shi, A. V. Smith, R. A. Poot, B. St Pourcain, A. Teumer, G. Thorleifsson, N. Verweij, D. Vuckovic, J. Wellmann, H.-J. Westra, J. Yang, W. Zhao, Z. Zhu, B. Z. Alizadeh, N. Amin, A. Bakshi, S. E. Baumeister, G. Biino, K. Bønnelykke, P. A. Boyle, H. Campbell, F. P. Cappuccio, G. Davies, J.-E. De Neve, P. Deloukas, I. Demuth, J. Ding, P. Eibich, L. Eisele, N. Eklund, D. M. Evans, J. D. Faul, M. F. Feitosa, A. J. Forstner, I. Gandin, B. Gunnarsson, B. V. Halldórsson, T. B. Harris, A. C. Heath, L. J. Hocking, E. G. Holliday, G. Homuth, M. A. Horan, J.-J. Hottenga, P. L. de Jager, P. K. Joshi, A. Jugessur, M. A. Kaakinen, M. Kähönen, S. Kanoni, L. Keltigangas-Järvinen, L. A. L. M. Kiemeney, I. Kolcic, S. Koskinen, A. T. Kraja, M. Kroh, Z. Kutalik, A. Latvala, L. J. Launer, M. P. Lebreton, D. F. Levinson, P. Lichtenstein, P. Lichtner, D. C. M. Liewald, LifeLines Cohort Study, A. Loukola, P. A. Madden, R. Mägi, T. Mäki-Opas, R. E. Marioni, P. Marques-Vidal, G. A. Meddens, G. McMahon, C. Meisinger, T. Meitinger, Y. Milaneschi, L. Milani, G. W. Montgomery, R. Myhre, C. P. Nelson, D. R. Nyholt, W. E. R. Ollier, A. Palotie, L. Paternoster, N. L. Pedersen, K. E. Petrovic, D. J. Porteous, K. Räikkönen, S. M. Ring, A. Robino, O. Rostapshova, I. Rudan, A. Rustichini, V. Salomaa, A. R. Sanders, A.-P. Sarin, H. Schmidt, R. J. Scott, B. H. Smith, J. A. Smith, J. A. Staessen, E. Steinhagen-Thiessen, K. Strauch, A. Terracciano, M. D. Tobin, S. Ulivi, S. Vaccargiu, L. Quaye, F. J. A. van Rooij, C. Venturini, A. A. E. Vinkhuyzen, U. Völker, H. Völzke, J. M. Vonk, D. Vozzi, J. Waage, E. B. Ware, G. Willemsen, J. R. Attia, D. A. Bennett, K. Berger, L. Bertram, H. Bisgaard, D. I. Boomsma, I. B. Borecki, U. Bültmann, C. F. Chabris, F. Cucca, D. Cusi, I. J. Deary, G. V. Dedoussis, C. M. van Duijn, J. G. Eriksson, B. Franke, L. Franke, P. Gasparini, P. V. Gejman, C. Gieger, H.-J. Grabe, J. Gratten, P. J. F. Groenen, V. Gudnason, P. van der Harst, C. Hayward, D. A. Hinds, W. Hoffmann, E. Hyppönen, W. G. Iacono, B. Jacobsson, M.-R. Järvelin, K.-H. Jöckel, J. Kaprio, S. L. R. Kardia, T. Lehtimäki, S. F. Lehrer, P. K. E. Magnusson, N. G. Martin, M. McGue, A. Metspalu, N. Pendleton, B. W. J. H. Penninx, M. Perola, N. Pirastu, M. Pirastu, O. Polasek, D. Posthuma, C. Power, M. A. Province, N. J. Samani, D. Schlessinger, R. Schmidt, T. I. A. Sørensen, T. D. Spector, K. Stefansson, U. Thorsteinsdottir, A. R. Thurik, N. J. Timpson, H. Tiemeier, J. Y. Tung, A. G. Uitterlinden, V. Vitart, P. Vollenweider, D. R. Weir, J. F. Wilson, A. F. Wright, D. C. Conley, R. F. Krueger, G. Davey Smith, A. Hofman, D. I. Laibson, S. E. Medland, M. N. Meyer, J. Yang, M. Johannesson, P. M. Visscher, T. Esko, P. D. Koellinger, D. Cesarini, D. J. Benjamin, Genome-wide association study identifies 74 loci associated with educational attainment. *Nature*. **533**, 539–542 (2016).](http://paperpile.com/b/cCKYZi/iDSby)

82. [G. Schumann, C. Liu, P. O’Reilly, H. Gao, P. Song, B. Xu, B. Ruggeri, N. Amin, T. Jia, S. Preis, M. Segura Lepe, S. Akira, C. Barbieri, S. Baumeister, S. Cauchi, T.-K. Clarke, S. Enroth, K. Fischer, J. Hällfors, S. E. Harris, S. Hieber, E. Hofer, J.-J. Hottenga, Å. Johansson, P. K. Joshi, N. Kaartinen, J. Laitinen, R. Lemaitre, A. Loukola, J. ’an Luan, L.-P. Lyytikäinen, M. Mangino, A. Manichaikul, H. Mbarek, Y. Milaneschi, A. Moayyeri, K. Mukamal, C. Nelson, J. Nettleton, E. Partinen, R. Rawal, A. Robino, L. Rose, C. Sala, T. Satoh, R. Schmidt, K. Schraut, R. Scott, A. V. Smith, J. M. Starr, A. Teumer, S. Trompet, A. G. Uitterlinden, C. Venturini, A.-C. Vergnaud, N. Verweij, V. Vitart, D. Vuckovic, J. Wedenoja, L. Yengo, B. Yu, W. Zhang, J. H. Zhao, D. I. Boomsma, J. Chambers, D. I. Chasman, T. Daniela, E. de Geus, I. Deary, J. G. Eriksson, T. Esko, V. Eulenburg, O. H. Franco, P. Froguel, C. Gieger, H. J. Grabe, V. Gudnason, U. Gyllensten, T. B. Harris, A.-L. Hartikainen, A. C. Heath, L. Hocking, A. Hofman, C. Huth, M.-R. Jarvelin, J. W. Jukema, J. Kaprio, J. S. Kooner, Z. Kutalik, J. Lahti, C. Langenberg, T. Lehtimäki, Y. Liu, P. A. F. Madden, N. Martin, A. Morrison, B. Penninx, N. Pirastu, B. Psaty, O. Raitakari, P. Ridker, R. Rose, J. I. Rotter, N. J. Samani, H. Schmidt, T. D. Spector, D. Stott, D. Strachan, I. Tzoulaki, P. van der Harst, C. M. van Duijn, P. Marques-Vidal, P. Vollenweider, N. J. Wareham, J. B. Whitfield, J. Wilson, B. Wolffenbuttel, G. Bakalkin, E. Evangelou, Y. Liu, K. M. Rice, S. Desrivières, S. A. Kliewer, D. J. Mangelsdorf, C. P. Müller, D. Levy, P. Elliott, KLB is associated with alcohol drinking, and its gene product β-Klotho is necessary for FGF21 regulation of alcohol preference. *Proc. Natl. Acad. Sci. U. S. A.* **113**, 14372–14377 (2016).](http://paperpile.com/b/cCKYZi/cJmDu)

83. [I. E. Jansen, J. E. Savage, K. Watanabe, J. Bryois, D. M. Williams, S. Steinberg, J. Sealock, I. K. Karlsson, S. Hägg, L. Athanasiu, N. Voyle, P. Proitsi, A. Witoelar, S. Stringer, D. Aarsland, I. S. Almdahl, F. Andersen, S. Bergh, F. Bettella, S. Bjornsson, A. Brækhus, G. Bråthen, C. de Leeuw, R. S. Desikan, S. Djurovic, L. Dumitrescu, T. Fladby, T. J. Hohman, P. V. Jonsson, S. J. Kiddle, A. Rongve, I. Saltvedt, S. B. Sando, G. Selbæk, M. Shoai, N. G. Skene, J. Snaedal, E. Stordal, I. D. Ulstein, Y. Wang, L. R. White, J. Hardy, J. Hjerling-Leffler, P. F. Sullivan, W. M. van der Flier, R. Dobson, L. K. Davis, H. Stefansson, K. Stefansson, N. L. Pedersen, S. Ripke, O. A. Andreassen, D. Posthuma, Genome-wide meta-analysis identifies new loci and functional pathways influencing Alzheimer’s disease risk. *Nat. Genet.* **51**, 404–413 (2019).](http://paperpile.com/b/cCKYZi/a4aSi)

84. [E. A. Stahl, G. Breen, A. J. Forstner, A. McQuillin, S. Ripke, V. Trubetskoy, M. Mattheisen, Y. Wang, J. R. I. Coleman, H. A. Gaspar, C. A. de Leeuw, S. Steinberg, J. M. W. Pavlides, M. Trzaskowski, E. M. Byrne, T. H. Pers, P. A. Holmans, A. L. Richards, L. Abbott, E. Agerbo, H. Akil, D. Albani, N. Alliey-Rodriguez, T. D. Als, A. Anjorin, V. Antilla, S. Awasthi, J. A. Badner, M. Bækvad-Hansen, J. D. Barchas, N. Bass, M. Bauer, R. Belliveau, S. E. Bergen, C. B. Pedersen, E. Bøen, M. P. Boks, J. Boocock, M. Budde, W. Bunney, M. Burmeister, J. Bybjerg-Grauholm, W. Byerley, M. Casas, F. Cerrato, P. Cervantes, K. Chambert, A. W. Charney, D. Chen, C. Churchhouse, T.-K. Clarke, W. Coryell, D. W. Craig, C. Cruceanu, D. Curtis, P. M. Czerski, A. M. Dale, S. de Jong, F. Degenhardt, J. Del-Favero, J. R. DePaulo, S. Djurovic, A. L. Dobbyn, A. Dumont, T. Elvsåshagen, V. Escott-Price, C. C. Fan, S. B. Fischer, M. Flickinger, T. M. Foroud, L. Forty, J. Frank, C. Fraser, N. B. Freimer, L. Frisén, K. Gade, D. Gage, J. Garnham, C. Giambartolomei, M. G. Pedersen, J. Goldstein, S. D. Gordon, K. Gordon-Smith, E. K. Green, M. J. Green, T. A. Greenwood, J. Grove, W. Guan, J. Guzman-Parra, M. L. Hamshere, M. Hautzinger, U. Heilbronner, S. Herms, M. Hipolito, P. Hoffmann, D. Holland, L. Huckins, S. Jamain, J. S. Johnson, A. Juréus, R. Kandaswamy, R. Karlsson, J. L. Kennedy, S. Kittel-Schneider, J. A. Knowles, M. Kogevinas, A. C. Koller, R. Kupka, C. Lavebratt, J. Lawrence, W. B. Lawson, M. Leber, P. H. Lee, S. E. Levy, J. Z. Li, C. Liu, S. Lucae, A. Maaser, D. J. MacIntyre, P. B. Mahon, W. Maier, L. Martinsson, S. McCarroll, P. McGuffin, M. G. McInnis, J. D. McKay, H. Medeiros, S. E. Medland, F. Meng, L. Milani, G. W. Montgomery, D. W. Morris, T. W. Mühleisen, N. Mullins, H. Nguyen, C. M. Nievergelt, A. N. Adolfsson, E. A. Nwulia, C. O’Donovan, L. M. O. Loohuis, A. P. S. Ori, L. Oruc, U. Ösby, R. H. Perlis, A. Perry, A. Pfennig, J. B. Potash, S. M. Purcell, E. J. Regeer, A. Reif, C. S. Reinbold, J. P. Rice, F. Rivas, M. Rivera, P. Roussos, D. M. Ruderfer, E. Ryu, C. Sánchez-Mora, A. F. Schatzberg, W. A. Scheftner, N. J. Schork, C. Shannon Weickert, T. Shehktman, P. D. Shilling, E. Sigurdsson, C. Slaney, O. B. Smeland, J. L. Sobell, C. Søholm Hansen, A. T. Spijker, D. St Clair, M. Steffens, J. S. Strauss, F. Streit, J. Strohmaier, S. Szelinger, R. C. Thompson, T. E. Thorgeirsson, J. Treutlein, H. Vedder, W. Wang, S. J. Watson, T. W. Weickert, S. H. Witt, S. Xi, W. Xu, A. H. Young, P. Zandi, P. Zhang, S. Zöllner, eQTLGen Consortium, BIOS Consortium, R. Adolfsson, I. Agartz, M. Alda, L. Backlund, B. T. Baune, F. Bellivier, W. H. Berrettini, J. M. Biernacka, D. H. R. Blackwood, M. Boehnke, A. D. Børglum, A. Corvin, N. Craddock, M. J. Daly, U. Dannlowski, T. Esko, B. Etain, M. Frye, J. M. Fullerton, E. S. Gershon, M. Gill, F. Goes, M. Grigoroiu-Serbanescu, J. Hauser, D. M. Hougaard, C. M. Hultman, I. Jones, L. A. Jones, R. S. Kahn, G. Kirov, M. Landén, M. Leboyer, C. M. Lewis, Q. S. Li, J. Lissowska, N. G. Martin, F. Mayoral, S. L. McElroy, A. M. McIntosh, F. J. McMahon, I. Melle, A. Metspalu, P. B. Mitchell, G. Morken, O. Mors, P. B. Mortensen, B. Müller-Myhsok, R. M. Myers, B. M. Neale, V. Nimgaonkar, M. Nordentoft, M. M. Nöthen, M. C. O’Donovan, K. J. Oedegaard, M. J. Owen, S. A. Paciga, C. Pato, M. T. Pato, D. Posthuma, J. A. Ramos-Quiroga, M. Ribasés, M. Rietschel, G. A. Rouleau, M. Schalling, P. R. Schofield, T. G. Schulze, A. Serretti, J. W. Smoller, H. Stefansson, K. Stefansson, E. Stordal, P. F. Sullivan, G. Turecki, A. E. Vaaler, E. Vieta, J. B. Vincent, T. Werge, J. I. Nurnberger, N. R. Wray, A. Di Florio, H. J. Edenberg, S. Cichon, R. A. Ophoff, L. J. Scott, O. A. Andreassen, J. Kelsoe, P. Sklar, Bipolar Disorder Working Group of the Psychiatric Genomics Consortium, Genome-wide association study identifies 30 loci associated with bipolar disorder. *Nat. Genet.* **51**, 793–803 (2019).](http://paperpile.com/b/cCKYZi/yWiI0)

85. [D. Demontis, R. K. Walters, J. Martin, M. Mattheisen, T. D. Als, E. Agerbo, G. Baldursson, R. Belliveau, J. Bybjerg-Grauholm, M. Bækvad-Hansen, F. Cerrato, K. Chambert, C. Churchhouse, A. Dumont, N. Eriksson, M. Gandal, J. I. Goldstein, K. L. Grasby, J. Grove, O. O. Gudmundsson, C. S. Hansen, M. E. Hauberg, M. V. Hollegaard, D. P. Howrigan, H. Huang, J. B. Maller, A. R. Martin, N. G. Martin, J. Moran, J. Pallesen, D. S. Palmer, C. B. Pedersen, M. G. Pedersen, T. Poterba, J. B. Poulsen, S. Ripke, E. B. Robinson, F. K. Satterstrom, H. Stefansson, C. Stevens, P. Turley, G. B. Walters, H. Won, M. J. Wright, ADHD Working Group of the Psychiatric Genomics Consortium (PGC), Early Lifecourse & Genetic Epidemiology (EAGLE) Consortium, 23andMe Research Team, O. A. Andreassen, P. Asherson, C. L. Burton, D. I. Boomsma, B. Cormand, S. Dalsgaard, B. Franke, J. Gelernter, D. Geschwind, H. Hakonarson, J. Haavik, H. R. Kranzler, J. Kuntsi, K. Langley, K.-P. Lesch, C. Middeldorp, A. Reif, L. A. Rohde, P. Roussos, R. Schachar, P. Sklar, E. J. S. Sonuga-Barke, P. F. Sullivan, A. Thapar, J. Y. Tung, I. D. Waldman, S. E. Medland, K. Stefansson, M. Nordentoft, D. M. Hougaard, T. Werge, O. Mors, P. B. Mortensen, M. J. Daly, S. V. Faraone, A. D. Børglum, B. M. Neale, Discovery of the first genome-wide significant risk loci for attention deficit/hyperactivity disorder. *Nat. Genet.* **51**, 63–75 (2019).](http://paperpile.com/b/cCKYZi/GIfIW)

86. [M. Luciano, S. P. Hagenaars, G. Davies, W. D. Hill, T.-K. Clarke, M. Shirali, S. E. Harris, R. E. Marioni, D. C. Liewald, C. Fawns-Ritchie, M. J. Adams, D. M. Howard, C. M. Lewis, C. R. Gale, A. M. McIntosh, I. J. Deary, Association analysis in over 329,000 individuals identifies 116 independent variants influencing neuroticism. *Nat. Genet.* **50**, 6–11 (2018).](http://paperpile.com/b/cCKYZi/4f4uL)

87. [Schizophrenia Working Group of the Psychiatric Genomics Consortium, Biological insights from 108 schizophrenia-associated genetic loci. *Nature*. **511**, 421–427 (2014).](http://paperpile.com/b/cCKYZi/h8xSc)

88. [W. van Rheenen, A. Shatunov, A. M. Dekker, R. L. McLaughlin, F. P. Diekstra, S. L. Pulit, R. A. A. van der Spek, U. Võsa, S. de Jong, M. R. Robinson, J. Yang, I. Fogh, P. T. van Doormaal, G. H. P. Tazelaar, M. Koppers, A. M. Blokhuis, W. Sproviero, A. R. Jones, K. P. Kenna, K. R. van Eijk, O. Harschnitz, R. D. Schellevis, W. J. Brands, J. Medic, A. Menelaou, A. Vajda, N. Ticozzi, K. Lin, B. Rogelj, K. Vrabec, M. Ravnik-Glavač, B. Koritnik, J. Zidar, L. Leonardis, L. D. Grošelj, S. Millecamps, F. Salachas, V. Meininger, M. de Carvalho, S. Pinto, J. S. Mora, R. Rojas-García, M. Polak, S. Chandran, S. Colville, R. Swingler, K. E. Morrison, P. J. Shaw, J. Hardy, R. W. Orrell, A. Pittman, K. Sidle, P. Fratta, A. Malaspina, S. Topp, S. Petri, S. Abdulla, C. Drepper, M. Sendtner, T. Meyer, R. A. Ophoff, K. A. Staats, M. Wiedau-Pazos, C. Lomen-Hoerth, V. M. Van Deerlin, J. Q. Trojanowski, L. Elman, L. McCluskey, A. N. Basak, C. Tunca, H. Hamzeiy, Y. Parman, T. Meitinger, P. Lichtner, M. Radivojkov-Blagojevic, C. R. Andres, C. Maurel, G. Bensimon, B. Landwehrmeyer, A. Brice, C. A. M. Payan, S. Saker-Delye, A. Dürr, N. W. Wood, L. Tittmann, W. Lieb, A. Franke, M. Rietschel, S. Cichon, M. M. Nöthen, P. Amouyel, C. Tzourio, J.-F. Dartigues, A. G. Uitterlinden, F. Rivadeneira, K. Estrada, A. Hofman, C. Curtis, H. M. Blauw, A. J. van der Kooi, M. de Visser, A. Goris, M. Weber, C. E. Shaw, B. N. Smith, O. Pansarasa, C. Cereda, R. Del Bo, G. P. Comi, S. D’Alfonso, C. Bertolin, G. Sorarù, L. Mazzini, V. Pensato, C. Gellera, C. Tiloca, A. Ratti, A. Calvo, C. Moglia, M. Brunetti, S. Arcuti, R. Capozzo, C. Zecca, C. Lunetta, S. Penco, N. Riva, A. Padovani, M. Filosto, B. Muller, R. J. Stuit, PARALS Registry, SLALOM Group, SLAP Registry, FALS Sequencing Consortium, SLAGEN Consortium, NNIPPS Study Group, I. Blair, K. Zhang, E. P. McCann, J. A. Fifita, G. A. Nicholson, D. B. Rowe, R. Pamphlett, M. C. Kiernan, J. Grosskreutz, O. W. Witte, T. Ringer, T. Prell, B. Stubendorff, I. Kurth, C. A. Hübner, P. N. Leigh, F. Casale, A. Chio, E. Beghi, E. Pupillo, R. Tortelli, G. Logroscino, J. Powell, A. C. Ludolph, J. H. Weishaupt, W. Robberecht, P. Van Damme, L. Franke, T. H. Pers, R. H. Brown, J. D. Glass, J. E. Landers, O. Hardiman, P. M. Andersen, P. Corcia, P. Vourc’h, V. Silani, N. R. Wray, P. M. Visscher, P. I. W. de Bakker, M. A. van Es, R. J. Pasterkamp, C. M. Lewis, G. Breen, A. Al-Chalabi, L. H. van den Berg, J. H. Veldink, Genome-wide association analyses identify new risk variants and the genetic architecture of amyotrophic lateral sclerosis. *Nat. Genet.* **48**, 1043–1048 (2016).](http://paperpile.com/b/cCKYZi/kfrEn)

89. [W. Zhou, J. B. Nielsen, L. G. Fritsche, R. Dey, M. E. Gabrielsen, B. N. Wolford, J. LeFaive, P. VandeHaar, S. A. Gagliano, A. Gifford, L. A. Bastarache, W.-Q. Wei, J. C. Denny, M. Lin, K. Hveem, H. M. Kang, G. R. Abecasis, C. J. Willer, S. Lee, Efficiently controlling for case-control imbalance and sample relatedness in large-scale genetic association studies. *Nat. Genet.* **50**, 1335–1341 (2018).](http://paperpile.com/b/cCKYZi/CiUtF)

90. [P. R. Jansen, K. Watanabe, S. Stringer, N. Skene, J. Bryois, A. R. Hammerschlag, C. A. de Leeuw, J. S. Benjamins, A. B. Muñoz-Manchado, M. Nagel, J. E. Savage, H. Tiemeier, T. White, 23andMe Research Team, J. Y. Tung, D. A. Hinds, V. Vacic, X. Wang, P. F. Sullivan, S. van der Sluis, T. J. C. Polderman, A. B. Smit, J. Hjerling-Leffler, E. J. W. Van Someren, D. Posthuma, Genome-wide analysis of insomnia in 1,331,010 individuals identifies new risk loci and functional pathways. *Nat. Genet.* **51**, 394–403 (2019).](http://paperpile.com/b/cCKYZi/EKAOk)

91. [C. P. Nelson, A. Goel, A. S. Butterworth, S. Kanoni, T. R. Webb, E. Marouli, L. Zeng, I. Ntalla, F. Y. Lai, J. C. Hopewell, O. Giannakopoulou, T. Jiang, S. E. Hamby, E. Di Angelantonio, T. L. Assimes, E. P. Bottinger, J. C. Chambers, R. Clarke, C. N. A. Palmer, R. M. Cubbon, P. Ellinor, R. Ermel, E. Evangelou, P. W. Franks, C. Grace, D. Gu, A. D. Hingorani, J. M. M. Howson, E. Ingelsson, A. Kastrati, T. Kessler, T. Kyriakou, T. Lehtimäki, X. Lu, Y. Lu, W. März, R. McPherson, A. Metspalu, M. Pujades-Rodriguez, A. Ruusalepp, E. E. Schadt, A. F. Schmidt, M. J. Sweeting, P. A. Zalloua, K. AlGhalayini, B. D. Keavney, J. S. Kooner, R. J. F. Loos, R. S. Patel, M. K. Rutter, M. Tomaszewski, I. Tzoulaki, E. Zeggini, J. Erdmann, G. Dedoussis, J. L. M. Björkegren, EPIC-CVD Consortium, CARDIoGRAMplusC4D, UK Biobank CardioMetabolic Consortium CHD working group, H. Schunkert, M. Farrall, J. Danesh, N. J. Samani, H. Watkins, P. Deloukas, Association analyses based on false discovery rate implicate new loci for coronary artery disease. *Nat. Genet.* **49**, 1385–1391 (2017).](http://paperpile.com/b/cCKYZi/6Yumh)

92. [V. Deary, S. P. Hagenaars, S. E. Harris, W. D. Hill, G. Davies, D. C. M. Liewald, International Consortium for Blood Pressure GWAS, CHARGE consortium Aging and Longevity Group, CHARGE consortium Inflammation Group, A. M. McIntosh, C. R. Gale, I. J. Deary, Genetic contributions to self-reported tiredness. *Mol. Psychiatry*. **23**, 789–790 (2018).](http://paperpile.com/b/cCKYZi/NWMm0)

93. [J. Chiou, R. J. Geusz, M.-L. Okino, J. Y. Han, M. Miller, R. Melton, E. Beebe, P. Benaglio, S. Huang, K. Korgaonkar, S. Heller, A. Kleger, S. Preissl, D. U. Gorkin, M. Sander, K. J. Gaulton, Interpreting type 1 diabetes risk with genetics and single-cell epigenomics. *Nature*. **594**, 398–402 (2021).](http://paperpile.com/b/cCKYZi/Vj2T9)

94. [A. Mahajan, D. Taliun, M. Thurner, N. R. Robertson, J. M. Torres, N. W. Rayner, A. J. Payne, V. Steinthorsdottir, R. A. Scott, N. Grarup, J. P. Cook, E. M. Schmidt, M. Wuttke, C. Sarnowski, R. Mägi, J. Nano, C. Gieger, S. Trompet, C. Lecoeur, M. H. Preuss, B. P. Prins, X. Guo, L. F. Bielak, J. E. Below, D. W. Bowden, J. C. Chambers, Y. J. Kim, M. C. Y. Ng, L. E. Petty, X. Sim, W. Zhang, A. J. Bennett, J. Bork-Jensen, C. M. Brummett, M. Canouil, K.-U. Ec Kardt, K. Fischer, S. L. R. Kardia, F. Kronenberg, K. Läll, C.-T. Liu, A. E. Locke, J. ’an Luan, I. Ntalla, V. Nylander, S. Schönherr, C. Schurmann, L. Yengo, E. P. Bottinger, I. Brandslund, C. Christensen, G. Dedoussis, J. C. Florez, I. Ford, O. H. Franco, T. M. Frayling, V. Giedraitis, S. Hackinger, A. T. Hattersley, C. Herder, M. A. Ikram, M. Ingelsson, M. E. Jørgensen, T. Jørgensen, J. Kriebel, J. Kuusisto, S. Ligthart, C. M. Lindgren, A. Linneberg, V. Lyssenko, V. Mamakou, T. Meitinger, K. L. Mohlke, A. D. Morris, G. Nadkarni, J. S. Pankow, A. Peters, N. Sattar, A. Stančáková, K. Strauch, K. D. Taylor, B. Thorand, G. Thorleifsson, U. Thorsteinsdottir, J. Tuomilehto, D. R. Witte, J. Dupuis, P. A. Peyser, E. Zeggini, R. J. F. Loos, P. Froguel, E. Ingelsson, L. Lind, L. Groop, M. Laakso, F. S. Collins, J. W. Jukema, C. N. A. Palmer, H. Grallert, A. Metspalu, A. Dehghan, A. Köttgen, G. R. Abecasis, J. B. Meigs, J. I. Rotter, J. Marchini, O. Pedersen, T. Hansen, C. Langenberg, N. J. Wareham, K. Stefansson, A. L. Gloyn, A. P. Morris, M. Boehnke, M. I. McCarthy, Fine-mapping type 2 diabetes loci to single-variant resolution using high-density imputation and islet-specific epigenome maps. *Nat. Genet.* **50**, 1505–1513 (2018).](http://paperpile.com/b/cCKYZi/Waqsi)

95. [M. A. Ferreira, J. M. Vonk, H. Baurecht, I. Marenholz, C. Tian, J. D. Hoffman, Q. Helmer, A. Tillander, V. Ullemar, J. van Dongen, Y. Lu, F. Rüschendorf, J. Esparza-Gordillo, C. W. Medway, E. Mountjoy, K. Burrows, O. Hummel, S. Grosche, B. M. Brumpton, J. S. Witte, J.-J. Hottenga, G. Willemsen, J. Zheng, E. Rodríguez, M. Hotze, A. Franke, J. A. Revez, J. Beesley, M. C. Matheson, S. C. Dharmage, L. M. Bain, L. G. Fritsche, M. E. Gabrielsen, B. Balliu, 23andMe Research Team, AAGC collaborators, BIOS consortium, LifeLines Cohort Study, J. B. Nielsen, W. Zhou, K. Hveem, A. Langhammer, O. L. Holmen, M. Løset, G. R. Abecasis, C. J. Willer, A. Arnold, G. Homuth, C. O. Schmidt, P. J. Thompson, N. G. Martin, D. L. Duffy, N. Novak, H. Schulz, S. Karrasch, C. Gieger, K. Strauch, R. B. Melles, D. A. Hinds, N. Hübner, S. Weidinger, P. K. E. Magnusson, R. Jansen, E. Jorgenson, Y.-A. Lee, D. I. Boomsma, C. Almqvist, R. Karlsson, G. H. Koppelman, L. Paternoster, Shared genetic origin of asthma, hay fever and eczema elucidates allergic disease biology. *Nat. Genet.* **49**, 1752–1757 (2017).](http://paperpile.com/b/cCKYZi/OMo8u)

96. [R. J. P. van der Valk, E. Kreiner-Møller, M. N. Kooijman, M. Guxens, E. Stergiakouli, A. Sääf, J. P. Bradfield, F. Geller, M. G. Hayes, D. L. Cousminer, A. Körner, E. Thiering, J. A. Curtin, R. Myhre, V. Huikari, R. Joro, M. Kerkhof, N. M. Warrington, N. Pitkänen, I. Ntalla, M. Horikoshi, R. Veijola, R. M. Freathy, Y.-Y. Teo, S. J. Barton, D. M. Evans, J. P. Kemp, B. St Pourcain, S. M. Ring, G. Davey Smith, A. Bergström, I. Kull, H. Hakonarson, F. D. Mentch, H. Bisgaard, B. Chawes, J. Stokholm, J. Waage, P. Eriksen, A. Sevelsted, M. Melbye, Early Genetics and Lifecourse Epidemiology (EAGLE) Consortium, C. M. van Duijn, C. Medina-Gomez, A. Hofman, J. C. de Jongste, H. R. Taal, A. G. Uitterlinden, Genetic Investigation of ANthropometric Traits (GIANT) Consortium, L. L. Armstrong, J. Eriksson, A. Palotie, M. Bustamante, X. Estivill, J. R. Gonzalez, S. Llop, W. Kiess, A. Mahajan, C. Flexeder, C. M. T. Tiesler, C. S. Murray, A. Simpson, P. Magnus, V. Sengpiel, A.-L. Hartikainen, S. Keinanen-Kiukaanniemi, A. Lewin, A. Da Silva Couto Alves, A. I. Blakemore, J. L. Buxton, M. Kaakinen, A. Rodriguez, S. Sebert, M. Vaarasmaki, T. Lakka, V. Lindi, U. Gehring, D. S. Postma, W. Ang, J. P. Newnham, L.-P. Lyytikäinen, K. Pahkala, O. T. Raitakari, K. Panoutsopoulou, E. Zeggini, D. I. Boomsma, M. Groen-Blokhuis, J. Ilonen, L. Franke, J. N. Hirschhorn, T. H. Pers, L. Liang, J. Huang, B. Hocher, M. Knip, S.-M. Saw, J. W. Holloway, E. Melén, S. F. A. Grant, B. Feenstra, W. L. Lowe, E. Widén, E. Sergeyev, H. Grallert, A. Custovic, B. Jacobsson, M.-R. Jarvelin, M. Atalay, G. H. Koppelman, C. E. Pennell, H. Niinikoski, G. V. Dedoussis, M. I. Mccarthy, T. M. Frayling, J. Sunyer, N. J. Timpson, F. Rivadeneira, K. Bønnelykke, V. W. V. Jaddoe, Early Growth Genetics (EGG) Consortium, A novel common variant in DCST2 is associated with length in early life and height in adulthood. *Hum. Mol. Genet.* **24**, 1155–1168 (2015).](http://paperpile.com/b/cCKYZi/ozElW)

97. [M. Horikoshi, R. N. Beaumont, F. R. Day, N. M. Warrington, M. N. Kooijman, J. Fernandez-Tajes, B. Feenstra, N. R. van Zuydam, K. J. Gaulton, N. Grarup, J. P. Bradfield, D. P. Strachan, R. Li-Gao, T. S. Ahluwalia, E. Kreiner, R. Rueedi, L.-P. Lyytikäinen, D. L. Cousminer, Y. Wu, E. Thiering, C. A. Wang, C. T. Have, J.-J. Hottenga, N. Vilor-Tejedor, P. K. Joshi, E. T. H. Boh, I. Ntalla, N. Pitkänen, A. Mahajan, E. M. van Leeuwen, R. Joro, V. Lagou, M. Nodzenski, L. A. Diver, K. T. Zondervan, M. Bustamante, P. Marques-Vidal, J. M. Mercader, A. J. Bennett, N. Rahmioglu, D. R. Nyholt, R. C. W. Ma, C. H. T. Tam, W. H. Tam, CHARGE Consortium Hematology Working Group, S. K. Ganesh, F. J. van Rooij, S. E. Jones, P.-R. Loh, K. S. Ruth, M. A. Tuke, J. Tyrrell, A. R. Wood, H. Yaghootkar, D. M. Scholtens, L. Paternoster, I. Prokopenko, P. Kovacs, M. Atalay, S. M. Willems, K. Panoutsopoulou, X. Wang, L. Carstensen, F. Geller, K. E. Schraut, M. Murcia, C. E. van Beijsterveldt, G. Willemsen, E. V. R. Appel, C. E. Fonvig, C. Trier, C. M. Tiesler, M. Standl, Z. Kutalik, S. Bonas-Guarch, D. M. Hougaard, F. Sánchez, D. Torrents, J. Waage, M. V. Hollegaard, H. G. de Haan, F. R. Rosendaal, C. Medina-Gomez, S. M. Ring, G. Hemani, G. McMahon, N. R. Robertson, C. J. Groves, C. Langenberg, J. ’an Luan, R. A. Scott, J. H. Zhao, F. D. Mentch, S. M. MacKenzie, R. M. Reynolds, Early Growth Genetics (EGG) Consortium, W. L. Lowe Jr, A. Tönjes, M. Stumvoll, V. Lindi, T. A. Lakka, C. M. van Duijn, W. Kiess, A. Körner, T. I. Sørensen, H. Niinikoski, K. Pahkala, O. T. Raitakari, E. Zeggini, G. V. Dedoussis, Y.-Y. Teo, S.-M. Saw, M. Melbye, H. Campbell, J. F. Wilson, M. Vrijheid, E. J. de Geus, D. I. Boomsma, H. N. Kadarmideen, J.-C. Holm, T. Hansen, S. Sebert, A. T. Hattersley, L. J. Beilin, J. P. Newnham, C. E. Pennell, J. Heinrich, L. S. Adair, J. B. Borja, K. L. Mohlke, J. G. Eriksson, E. E. Widén, M. Kähönen, J. S. Viikari, T. Lehtimäki, P. Vollenweider, K. Bønnelykke, H. Bisgaard, D. O. Mook-Kanamori, A. Hofman, F. Rivadeneira, A. G. Uitterlinden, C. Pisinger, O. Pedersen, C. Power, E. Hyppönen, N. J. Wareham, H. Hakonarson, E. Davies, B. R. Walker, V. W. Jaddoe, M.-R. Jarvelin, S. F. Grant, A. A. Vaag, D. A. Lawlor, T. M. Frayling, G. Davey Smith, A. P. Morris, K. K. Ong, J. F. Felix, N. J. Timpson, J. R. Perry, D. M. Evans, M. I. McCarthy, R. M. Freathy, Genome-wide associations for birth weight and correlations with adult disease. *Nature*. **538**, 248–252 (2016).](http://paperpile.com/b/cCKYZi/Vs11F)

98. [1000 Genomes Project Consortium, A. Auton, L. D. Brooks, R. M. Durbin, E. P. Garrison, H. M. Kang, J. O. Korbel, J. L. Marchini, S. McCarthy, G. A. McVean, G. R. Abecasis, A global reference for human genetic variation. *Nature*. **526**, 68–74 (2015).](http://paperpile.com/b/cCKYZi/dsuF8)

99. [C. Y. McLean, D. Bristor, M. Hiller, S. L. Clarke, B. T. Schaar, C. B. Lowe, A. M. Wenger, G. Bejerano, GREAT improves functional interpretation of cis-regulatory regions. *Nat. Biotechnol.* **28**, 495–501 (2010).](http://paperpile.com/b/cCKYZi/sni9g)

100. [A. Heger, C. Webber, M. Goodson, C. P. Ponting, G. Lunter, GAT: a simulation framework for testing the association of genomic intervals. *Bioinformatics*. **29**, 2046–2048 (2013).](http://paperpile.com/b/cCKYZi/uaJFT)
